# Palladium Iodide Catalyzed Multicomponent Carbonylative Synthesis of 2-(4-Acylfuran-2-yl)acetamides

**DOI:** 10.3390/molecules28196764

**Published:** 2023-09-22

**Authors:** Ida Ziccarelli, Lucia Veltri, Tommaso Prestia, Roberta Amuso, Maria A. Chiacchio, Raffaella Mancuso, Bartolo Gabriele

**Affiliations:** 1 Laboratory of Industrial and Synthetic Organic Chemistry (LISOC), Department of Chemistry and Chemical Technologies, University of Calabria, Via Pietro Bucci 12/C, 87036 Arcavacata di Rende, Italy; ida.ziccarelli@unical.it (I.Z.); tommaso.prestia@gmail.com (T.P.); roberta.amuso@unical.it (R.A.); raffaella.mancuso@unical.it (R.M.); 2 Department of Drug Sciences, University of Catania, Viale A. Doria 6, 95125 Catania, Italy; ma.chiacchio@unict.it

**Keywords:** alkynes, amides, aminocarbonylation, carbonylation, C–H activation, conjugate addition, cyclization, furans, oxidative carbonylation, palladium

## Abstract

2-Propargyl-1,3-dicarbonyl compounds have been carbonylated under oxidative conditions and with the catalysis of the PdI_2_/KI catalytic system to selectively afford previously unreported 2-(4-acylfuran-2-yl)acetamides in fair to good yields (54–81%) over 19 examples. The process takes place under relatively mild conditions and occurs via a mechanistic pathway involving C*sp*-H activation by oxidative monoamincarbonylation of the terminal triple bond of the substrates with formation of 2-ynamide intermediates, followed by 5-*exo*-*dig O*-cyclization (via intramolecular conjugate addition of the in situ formed enolate to the 2-ynamide moiety) and aromative isomerization.

## 1. Introduction

Functionalized furans are a very important class of heterocyclic derivatives [1], known to possess important biological activities (see, for example, references [2,3]) and being useful precursors for further transformations (for a review, see reference [4]).

Among the synthetic methods available to prepare multisubstituted furans (for recent reviews, see references [5,6,7]), transition metal-catalyzed cyclization (TMCC) of suitable acyclic precursors is particularly attractive (for recent reviews, see references [8,9]; for a book, see reference [10]; for a recent example, see reference [11]). By this approach, the final compound with the desired substitution pattern can be obtained in one synthetic step starting from readily available substrates.

On the other hand, carbon monoxide is a very important C-1 building block in organic synthesis (for a recent book on carbonylation chemistry in organic synthesis, see reference [12]). In fact, CO can be installed in a large variety of organic substrates under different conditions, including transition-metal catalysis, to give high value–added carbonyl compounds (carbonylation reactions), including carbonylated heterocycles (for selected book chapters and reviews on metal-catalyzed carbonylation reactions, also leading to carbonylated heterocycles, see references [12,13,14,15,16,17,18,19,20,21,22,23]). Accordingly, the combination between TMCC and catalytic carbonylation with the appropriate acyclic precursor may represent an excellent entry to the direct synthesis of carbonyl-functionalized furan derivatives [12].

**Scheme 1:**
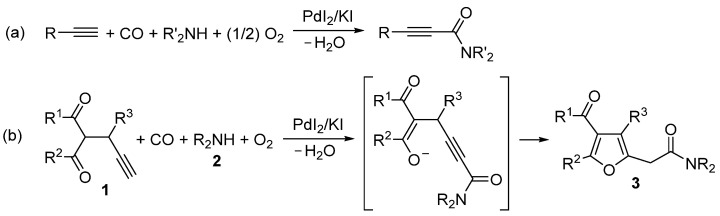
PdI_2_/KI-catalyzed oxidative monoaminocarbonylation of: (**a**) Simple alkyl- and arylacetylenes to give 2-ynamides [27,28] and (**b**) This work: 2-propargyl-1,3-dicarbonyl compounds **1** with amines **2** to 2-(4-acylfuran-2-yl)acetamides **3**.

In particular, the catalytic system based on PdI_2_ in conjunction with an excess of KI, developed by our research group [24,25] (for a recent review, see reference [26], has proved very valuable for the realization of several important carbonylative cyclization processes, particularly under oxidative conditions, with the one-step formation of carbonylated heterocyclic derivatives. Among the different routes that the PdI_2_/KI catalytic system is able to promote, the C*sp*-H activation by oxidative monoalkoxycarbonylation of the terminal triple bond, disclosed by our group in 2001 for the catalytic synthesis of 2-ynamides from simple alkyl- or arylacetylenes (Scheme 1a) [27,28], is particularly significant. In fact, this reactivity has been successfully employed for the synthesis of a plethora of heterocyclic derivatives, when applied to terminal alkyne substrates bearing a nucleophilic group in suitable position for the occurrence of an intramolecular conjugate addition to the initially formed 2-ynamide intermediate (Scheme 1b) (for a review, see reference [26]; for a very recent example, see reference [29]).

**Scheme 2:**
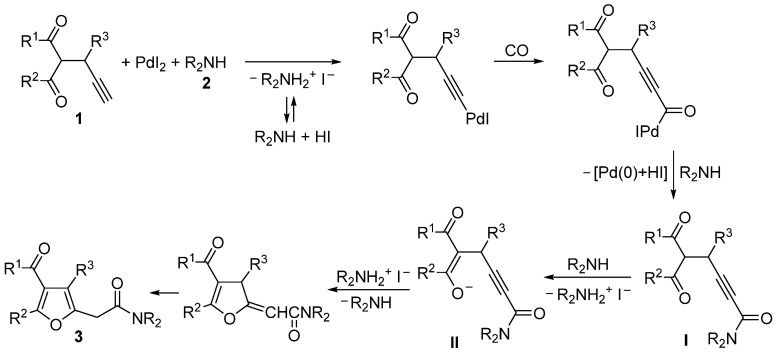
Mechanistic route leading to 2-(4-acylfuran-2-yl)acetamides **3** by PdI_2_/KI-catalyzed oxidative aminocarbonylation of 2-propargyl-1,3-dicarbonyl compounds **1** with secondary amines **2** through the formation of 2-ynamide intermediates **I** and **II**.

In this work, we report a new application of this concept, which allows the direct, multicomponent synthesis of previously unreported 2-(4-acylfuran-2-yl)acetamides **3** starting from readily available 2-propargyl-1,3-dicarbonyl compounds **1**, carbon monoxide, and a secondary amine **2**. In this case, the initially formed 2-ynamide intermediate **I** (formed by **2**-promoted C*sp*-H palladation of **1** followed by CO insertion and nucleophilic displacement by **2**) undergoes 5-*exo*-*dig* intramolecular conjugate addition from the oxygen of enolate moiety resulting from C-2 deprotonation by the basic amine, to give intermediate **II**, whose aromative isomerization eventually leads to 2-(4-acylfuran-2-yl)acetamides **3** (Scheme 2). It is worth noting that, as seen in the reaction of simple alkyl- and arylacetylenes (Scheme 1a) [27,28], the method is not applicable to the use of primary amines, as these compounds preferentially undergo oxidative carbonylation to ureas under our reaction conditions [30,31]. Moreover, as already seen for simple 1-alkynes [27,28], secondary amines of relatively low nucleophilicity (such as alkylarylamines of diarylamines) are not sufficiently reactive to give the reaction.

This approach, therefore, allows the direct synthesis of a new subclass of furan derivatives (2-(4-acylfuran-2-yl)acetamides **3**) by the catalytic assembly of very simple building blocks (2-propargyl-1,3-dicarbonyl compounds **1**, amines **2**, CO, and O_2_), with formation of water as benign coproduct.

## 2. Results and Discussion

We started our investigation using 3-(prop-2-yn-1-yl)pentane-2,4-dione **1a** as model substrate. The initial reaction was carried out in MeCN (0.10 mmol of **1a** per mL of MeCN) at 100 °C in the presence of PdI_2_ (1 mol%), KI (KI:PdI_2_ molar ratio = 100) and Et_2_NH **2a** (3 equiv) under 20 atm (at 25 °C) of a 4:1 mixture CO-air. After 15 h, **1a** conversion was complete, and, after column chromatography purification, 2-(4-acetyl-5-methylfuran-2-yl)-*N*,*N*-diethylacetamide **3aa** was recovered in 61% yield (based on starting **1a**), in perfect agreement with our work hypothesis (Table 1, entry 1).

After a brief optimization study, in which we varied some reaction parameters (such as solvent, amount of KI and amine, substrate concentration, and total pressure; Table 1), a 72% isolated yield of **3aa** was achieved under conditions similar to those of the first experiment, but with a higher substrate concentration (0.22 mmol of **1a** per mL of MeCN) and using 4 equiv of Et_2_NH **2a** (Table 2, entry 1). The reaction could also be performed with a lower catalyst loading (0.33 mol% PdI_2_, maintaining the KI:PdI_2_ molar ratio = 100), with an acceptable 55% isolated yield of **3aa** (Table 2, entry 2).

The reaction was then extended to other secondary amines **2b**–**f** (Table 2, entries 3–11) and different 2-propynyl-1,3-diketones **1b**–**g** (Table 2, entries 12–18). Similar results with respect to the parent reactions (Table 2, entries 1 and 2) were observed with other simple dialkylamines, such as Me_2_NH **2b** (Table 2, entries 3 and 4), Bu_2_NH **2c** (Table 2, entries 5 and 6), and even with sterically hindered *N*-ethylcyclohexylamine **2d** (Table 2, entries 7 and 8). Interestingly, more sterically demanding diisopropylamine **2e** was also reactive, and led to the formation of the corresponding furanacetamide **3ae** in 54% yield with 1 mol% catalyst (Table 2, entry 9). A cyclic amine like morpholine **2f** reacted well and delivered the corresponding product **3af** in either 74% yield (with 1 mol% PdI_2_; Table 2, entry 10) or 61% yield (with 0.33 mol% catalyst; Table 2, entry 11). With Et_2_NH **2a** or morpholine **2f** as the amine, extension of the protocol to other symmetrical 2-propargyl-1,3-diketones **1b**–**e** also led to good results, the corresponding furans being obtained in 66–81% yields with 1 mol% catalyst (Table 2, entries 12, 14, 16, 17) and 58–68% yields with 0.33 mol% catalyst (Table 2, entries 13, 15, 18). Only in the presence of a phenyl group α to the triple bond, as in 1,3-diphenyl-2-(1-phenylprop-2-yn-1-yl)propane-1,3-dione **1f**, a lower product yield was observed, probably for steric reasons (54%, Table 2, entry 19). Clearly, the use of an unsymmetrical diketonic substrate, such as 1-phenyl-2-(prop-2-yn-1-yl)butane-1,3-dione **1g**, afforded a mixture of isomeric products **3gf** and **3gf′**, which could be separated in 48% and 25% isolated yields, respectively (Table 2, entry 20). On the other hand, a selective process was observed with β-keto esters **1h**–**l**, owing to the higher nucleophilicity of the ketonic oxygen with respect to the estereal one [32], with selective formation of alkyl 5-(2-(dialkylamino)-2-oxoethyl)-2-alkylfuran-3-carboxylates (Table 2, entries 21–30). The method also worked nicely with 2-acetyl-*N*,*N*-diethylpent-4-ynamide **1m** and 1-phenyl-2-tosylpent-4-yn-1-one **1n**, with formation of 5-(2-(diethylamino)-2-oxoethyl)-*N*,*N*-diethyl-2-methylfuran-3-carboxamide **3ma** and *N*,*N*-dibutyl-2-(5-phenyl-4-tosylfuran-2-yl)acetamide **3nc**, respectively, in 57–72% yields (Table 2, entries 31–33).

## 3. Materials and Methods

### 3.1. General Experimental Methods

Melting points were measured with a Leitz Laborlux 12 POL polarizing optical microscope (Leitz Italia GmbH/Srl, Lana, BZ, Italy) and are uncorrected. ^1^H NMR and ^13^C NMR spectra were recorded at 25 °C in CDCl_3_ at 500 MHz and 125 MHz, respectively, with Me_4_Si as internal standard using Bruker DPX Avance 300 and Bruker DPX Avance 500 NMR Spectrometers (Bruker Italia s.r.l., Milano, Italy); chemical shifts (δ) and coupling constants (*J*) are given in ppm and in Hz, respectively. IR spectra were taken with a JASCO FT-IR 4200 spectrometer (Jasco Europe s.r.l., Cremella, Lecco, Italy). All reactions were analyzed by TLC on silica gel 60 F_254_ and by GC-MS analysis using a Shimadzu QP-2010 GC–MS apparatus (Shimadzu Italia s.r.l., Milano, Italy) at 70 eV ionization voltage equipped with a 95% methyl polysiloxane-5% phenyl polysiloxane capillary column (30 m × 0.25 mm, 0.25 μm). Column chromatography was performed on silica gel 60 (Merck, 70–230 mesh; Merck Life Science s.r.l., Milano, Italy). Evaporation refers to the removal of solvent under reduced pressure. The HRMS spectra were taken on an Agilent 1260 Infinity UHD accurate-mass Q-TOF-MS mass spectrometer (Agilent Technologies, Santa Clara, CA, USA), equipped with an electrospray ion source (ESI) operated in dual ion mode. A total of 10 μL of the sample solutions (CH_3_OH) were introduced by continuous infusion at a flow rate of 200 L min^−1^ with the aid of a syringe pump. Experimental conditions were performed as follows: capillary voltage, 4000 V; nebulizer pressure, 20 psi; flow rate of drying gas, 10 L/min; temperature of sheath gas, 325 °C; flow rate of sheath gas, 10 L/min; skimmer voltage, 60 V; OCT1 RF Vpp, 750 V; fragmentor voltage, 170 V. The spectra data were recorded in the *m*/*z* range of 100–1000 Da in a centroid pattern of full-scan MS analysis mode. The MS/MS data of the selected compounds were obtained by regulating diverse collision energy (18–45 eV).

### 3.2. Preparation of Substrates

Substrates **1** were prepared and characterized as described in Supplementary Materials. All other materials were commercially available and were used without further purification.

### 3.3. General Procedure for the Synthesis of 2-(4-Acylfuran-2-yl)acetamides **3**

A 35 mL stainless steel autoclave was charged in the presence of air with PdI_2_ (1.2 mg, 3.3 × 10^−3^ mmol, or 3.6 mg, 1.0 × 10^−2^ mmol; see Table 1), KI (55 mg, 0.33 mmol or 166 mg, 1.0 mmol), a solution of **1** [1.0 mmol; **1a**, 138 mg; **1b**, 166 mg; **1c**, 150 mg; **1d**, 152 mg; **1e**, 262 mg; **1f**, 338 mg; **1g**, 200 mg; **1h**, 154 mg; **1i**, 168 mg; **1j**, 196 mg; **1k**, 196 mg; **1l**, 230 mg; **1m**, 195 mg; **1n**, 312 mg] in anhydrous CH_3_CN (5.0 mL), and the amine **2** [4.0 mmol; **2a**, 292 mg; **2b**, 180 mg (2 mL of a 2 M solution in THF); **2c**, 516 mg; **2d**, 508 mg; **2e**, 404 mg; **2f**, 348 mg]. The autoclave was sealed and, while the mixture was stirred, the autoclave was pressurized with CO (16 atm) and air (up to 20 atm). After being stirred at 100 °C for 15 h, the autoclave was cooled, degassed, and opened. After evaporation of the solvent, products **3** were purified by column chromatography on silica gel using as eluent hexane-AcOEt from 8:2 to 6:4.

#### 3.3.1. 2-(4-Acetyl-5-methylfuran-2-yl)-*N*,*N*-diethylacetamide (**3aa**)

Yield: 170 mg, starting from 138 mg of **1a** (72%) (Table 1, entry 1). Yellow oil. IR (film): ν = 1667 (s), 1651 (s), 1566 (m), 1435 (m), 1404 (w), 1234 (m), 1134 (w), 949 (m), 795 (w) cm^−1^; ^1^H NMR (500 MHz, CDCl_3_): δ = 6.45 (s, 1 H), 3.67 (s, 2 H), 3.44–3.35 (m, 4 H), 2.55 (s, 3 H), 2.37 (s, 3 H), 1.20 (t, *J* = 7.1, 3 H), 1.15 (t, *J* = 7.1, 3 H); ^13^C NMR (125 MHz, CDCl_3_): δ = 194.2, 167.3, 157.7, 147.2, 122.2, 108.3, 42.5, 40.5, 33.2, 29.1, 14.34, 14.31, 12.9; GC-MS (EI): *m*/*z* = 237 (M^+^, 10), 137 (9), 100 (100), 72 (71); HRMS (ESI—TOF) *m*/*z*: [M + Na]^+^ Calcd. for C_13_H_19_NNaO_3_^+^ 260.1257; Found 260.1256.

#### 3.3.2. 2-(4-Acetyl-5-methylfuran-2-yl)-*N*,*N*-dimethylacetamide (**3ab**)

Yield: 140 mg, starting from 138 mg of **1a** (67%) (Table 1, entry 3). Yellow oil, IR (film): ν = 1686 (s), 1651 (s), 1557 (m), 1508 (w), 1396 (m), 1234 (m), 1138 (m), 1061 (w), 945 (m) cm^−1^; ^1^H NMR (500 MHz, CDCl_3_): δ = 6.44 (s, 1 H), 3.69 (s, 2 H), 3.10 (s, 3 H), 2.99 (m, 3 H), 2.55 (s, 3 H), 2.37 (s, 3 H); ^13^C NMR (125 MHz, CDCl_3_): δ = 194.2, 168.3, 157.8, 146.9, 122.3, 108.4, 37.7, 35.7, 33.4, 29.1, 14.3; GC-MS (EI): *m*/*z* = 209 (M^+^, 17), 137 (22), 72 (100); HRMS (ESI-TOF) *m*/*z*: [M + Na]^+^ Calcd. for C_11_H_15_NNaO_3_^+^ 232.0944; Found 232.0948.

#### 3.3.3. 2-(4-Acetyl-5-methylfuran-2-yl)-*N*,*N*-dibutylacetamide (**3ac**)

Yield: 219 mg, starting from 138 mg of **1a** (75%) (Table 1, entry 5). Yellow oil. IR (film): ν = 1678 (s), 1643 (s), 1570 (m), 1454 (m), 1431 (m), 1373 (w), 1223 (m), 1138 (w), 1114 (w), 945 (m) cm^−1^; ^1^H NMR (500 MHz, CDCl_3_): δ = 6.44 (s, 1 H), 3.67 (s, 2 H), 3.37–3.32 (m, 2 H), 3.31–3.26 (m, 2 H), 2.55 (s, 3 H), 2.37 (s, 3 H), 1.61–1.49 (m, 4 H), 1.40–1.27 (m, 4 H), 0.96 (t, *J* = 7.3, 3 H), 0.92 (t, *J* = 7.3, 3 H); ^13^C NMR (125 MHz, CDCl_3_): δ = 194.2, 167.7, 157.7, 147.3, 122.3, 108.3, 48.3, 46.1, 33.3, 33.2, 31.3, 29.8, 29.1, 20.3, 20.1, 14.4, 13.9; GC-MS (EI): *m*/*z* = 293 (M^+^, 4), 156 (38), 137 (11), 100 (27), 57 (100); HRMS (ESI-TOF) *m*/*z*: [M + Na]^+^ Calcd. for C_17_H_27_NNaO_3_^+^ 316.1883; Found 316.1898.

#### 3.3.4. 2-(4-Acetyl-5-methylfuran-2-yl)-*N*-cyclohexyl-*N*-ethylacetamide (Mixture of Rotamers A + B, Deriving from Hindered Rotation around the Amide Bond: A/B *ca* 1.2 by ^1^H NMR) (**3ad**)

Yield: 215 mg, starting from 138 mg of **1a** (74%) (Table 1, entry 7). Yellow oil. IR (film): ν = 1674 (m), 1643 (s), 1570 (w), 1427 (m), 1369 (w), 1234 (m), 1103 (w), 895 (w) cm^−1^; ^1^H NMR (500 MHz, CDCl_3_): δ = 6.45 [s, 1 H (A or B)], 6.44 [s, 1 H (B or A)], 4.31 [tt, *J* = 11.7, 3.5, 1 H (B)], 3.71 [s, 2 H (A)], 3.66 [s, 2 H (B)], 3.62 [tt, *J* = 11.7, 3.5, 1 H (A)], 3.37–3.27 [m, 2 H (A) + 2 H (B)], 2.56 [s, 3 H (A)], 2.55 [s, 3 H (B)], 2.37 [s, 3 H (A) + 3 H (B)], 1.90–1.60 [m, 4 H (A) + 4 H (B)], 1.56–1.22 [m, 6 H (A) + 6 H (B)], 1.23 [t, *J* = 7.2, 3 H (B)], 1.15 [t, *J* = 7.0, 3 H (A)]; ^13^C NMR (125 MHz, CDCl_3_): δ = 194.1 (A + B), 167.8 (B), 167.2 (A), 157.6 (A + B), 147.5 (A + B), 122.2 (A + B), 108.2 (A + B), 58.0 (A), 57.9 (B), 54.4 (A), 54.3 (B), 38.4 (B), 36.9 (A), 34.1 (A), 33.5 (B), 31.8 (A), 30.9 (B), 29.1 (A + B), 25.9 (A + B), 25.6 (B), 25.2 (A), 16.8 (A + B), 14.8 (A), 14.3 (B); GC-MS (EI): *m*/*z* = 291 (M^+^, 3), 154 (49), 137 (14), 123 (4), 95 (8), 83 (100); HRMS (ESI-TOF) *m*/*z*: [M + Na]^+^ Calcd. for C_17_H_25_NNaO_3_^+^ 314.1727; Found 314.1712.

#### 3.3.5. 2-(4-Acetyl-5-methylfuran-2-yl)-*N*,*N*-diisopropylacetamide (**3ae**)

Yield: 143 mg, starting from 138 mg of **1a** (54%) (Table 1, entry 9). Yellow oil. IR (film): ν = 1651 (s), 1643 (s), 1566 (m), 1454 (m), 1369 (m), 1339 (w), 1231 (w), 1211 (m), 1041 (w), 945 (w) cm^−1^; ^1^H NMR (500 MHz, CDCl_3_): δ = 6.43 (s, 1 H), 4.03 (heptuplet, *J* = 6.6, 1 H), 3.66 (s, 2 H), 3.57–3.44 (m, 1 H), 2.57 (s, 3 H), 2.37 (s, 3 H), 1.40 (d, *J* = 6.7), 1.21 (d, *J* = 6.6); ^13^C NMR (125 MHz, CDCl_3_): δ = 194.2, 166.9, 152.6, 147.6, 122.3, 108.2, 49.3, 46.1, 35.5, 29.1, 20.9, 20.6, 20.5, 14.4, 14.3; GC-MS (EI): *m*/*z* = 265 (M^+^, 2), 137 (19), 128 (53), 96 (7), 86 (94), 43 (100); HRMS (ESI-TOF) *m*/*z*: [M + Na]^+^ Calcd. for C_15_H_23_NNaO_3_^+^ 288.1570; Found 288.1561.

#### 3.3.6. 2-(4-Acetyl-5-methylfuran-2-yl)-1-morpholinoethan-1-one (**3af**)

Yield: 186 mg, starting from 138 mg of **1a** (74%) (Table 1, entry 10). Yellow oil. IR (film): ν = 1667 (s), 1651 (s), 1566 (m), 1435 (m), 1366 (w), 1273 (w), 1227 (m), 1111 (m), 1042 (w), 964 (w), 772 (m) cm^−1^; ^1^H NMR (500 MHz, CDCl_3_): δ = 6.45 (s, 1 H), 3.74–3.62 (m, 6 H), 3.70 (s, 2 H), 3.57–3.53 (m, 2 H), 2.56 (s, 3 H), 2.38 (s, 3 H); ^13^C NMR (125 MHz, CDCl_3_): δ = 194.0, 166.8, 157.8, 146.5, 122.3, 108.5, 66.7, 66.5, 46.6, 42.3, 33.1, 29.1, 14.3; GC-MS (EI): *m*/*z* = 251 (M^+^, 20), 137 (33), 114 (100), 70 (76); HRMS (ESI-TOF) *m*/*z*: [M + Na]^+^ Calcd. for C_13_H_17_NNaO_4_^+^ 274.1050; Found 274.1050.

#### 3.3.7. *N*,*N*-Diethyl-2-(5-ethyl-4-propionylfuran-2-yl)acetamide (**3ba**)

Yield: 214 mg, starting from 166 mg of **1b** (81%) (Table 1, entry 12). Colorless oil. IR (film): ν = 1674 (s), 1647 (s), 1562 (m), 1458 (m), 1431 (w), 1254 (w), 1219 (m), 1134 (w), 1011 (w), 926 (m) cm^−1^; ^1^H NMR (500 MHz, CDCl_3_): δ = 6.45 (s, br, 1 H), 3.68 (d, *J* = 0.7, 2 H), 3.42 (q, *J* = 7.1, 2 H), 3.39 (q, *J* = 7.1, 2 H), 2.99 (q, *J* = 7.5, 2 H), 2.72 (q, *J* = 7.3, 2 H), 1.22 (t, *J* = 7.5, 3 H), 1.19 (t, *J* = 7.1, 3 H), 1.15 (t, *J* = 7.1, 3 H), 1.13 (t, *J* = 7.3, 3 H); ^13^C NMR (125 MHz, CDCl_3_): δ = 197.2, 167.4, 162.5, 147.1, 120.8, 107.8, 42.5, 40.5, 34.4, 33.4, 21.6, 14.3, 12.9, 12.1, 7.9; GC-MS (EI): *m*/*z* = 265 (M^+^, 16), 165 (5), 100 (100), 72 (47); HRMS (ESI-TOF) *m*/*z*: [M + Na]^+^ Calcd. for C_15_H_23_NNaO_3_^+^ 288.1570; Found 288.1576.

#### 3.3.8. *N*,*N*-Diethyl-2-(4-oxo-4,5,6,7-tetrahydrobenzofuran-2-yl)acetamide (**3ca**)

Yield: 167 mg, starting from 150 mg of **1c** (67%) (Table 1, entry 14). Yellow oil. IR (film): ν = 1682 (s), 1667 (s), 1582 (w), 1442 (m), 1366 (w), 1219 (m), 1111 (m), 1003 (m), 772 (w) cm^−1^; ^1^H NMR (500 MHz, CDCl_3_): δ = 6.44 (s, 1 H), 3.70 (s, 2 H), 3.43–3.33 (m, 4 H), 2.86 (t, *J* = 6.3, 2 H), 2.47 (t, *J* = 6.3, 2 H), 2.16 (quintuplet, *J* = 6.3, 2 H), 1.20 (t, *J* = 7.1, 3 H), 1.14 (t, *J* = 7.1, 3 H); ^13^C NMR (125 MHz, CDCl_3_): δ = 194.4, 167.1, 166.8, 149.9, 122.1, 104.3, 42.5, 40.5, 37.6, 33.5, 23.4, 22.6, 14.3, 12.9; GC-MS (EI): *m*/*z* = 249 (M^+^, 10), 149 (6), 100 (100), 72 (50); HRMS (ESI-TOF) *m*/*z*: [M + Na]^+^ Calcd. for C_14_H_19_NNaO_3_^+^ 272.1257; Found 272.1261.

#### 3.3.9. 2-(4-Acetyl-3,5-dimethylfuran-2-yl)-1-morpholinoethan-1-one (**3df**)

Yield: 175 mg, starting from 152 mg of **1d** (66%) (Table 1, entry 16). Yellow solid, mp 90–91 °C. IR (film): ν = 1655 (s), 1558 (w), 1416 (m), 1354 (w), 1304 (w), 1231 (m), 1115 (m), 1072 (w), 1034 (w), 972 (m), 848 (m) cm^−1^; ^1^H NMR (500 MHz, CDCl_3_): δ = 3.69–3.62 (m, 6 H), 3.63 (s, 2 H), 3.57–3.53 (m, 2 H), 2.53 (s, 3 H), 2.42 (s, 3 H), 2.16 (s, 3 H); ^13^C NMR (125 MHz, CDCl_3_): δ = 194.8, 167.1, 157.3, 142.8, 123.3, 117.2, 66.8, 66.6, 46.5, 42.4, 31.3, 30.9, 15.3, 10.6; GC-MS (EI): *m*/*z* = 265 (M^+^, 26), 222 (3), 151 (100), 133 (6), 114 (40), 70 (30); HRMS (ESI-TOF) *m*/*z*: [M + Na]^+^ Calcd. for C_14_H_19_NNaO_4_^+^ 288.1206; Found 288.1216.

#### 3.3.10. 2-(4-Benzoyl-5-phenylfuran-2-yl)-*N*,*N*-diethylacetamide (**3ea**)

Yield: 245 mg, starting from 262 mg of **1e** (68%) (Table 1, entry 17). Colorless oil. IR (film): ν = 1647 (s), 1551 (w), 1485 (m), 1447 (m), 1381 (w), 1261 (m), 1227 (m), 1134 (w), 1072 (w), 887 (m), 729 (m), 694 (m) cm^−1^; ^1^H NMR (500 MHz, CDCl_3_): δ = 7.86–7.80 (m, 2 H), 7.68–7.63 (m, 2 H), 7.51–7.45 (m, 1 H), 7.38–7.33 (m, 2 H), 7.30–7.23 (m, 3 H), 6.53 (t, br, 1 H), 3.81 (d, *J* = 0.7, 2 H), 3.46–3.38 (m, 4 H), 1.22 (t, *J* = 7.1. 3 H), 1.16 (t, *J* = 7.1, 3 H); ^13^C NMR (125 MHz, CDCl_3_): δ = 191.7, 167.1, 155.3, 148.5, 138.1, 132.8, 129.7, 128.9, 128.3, 127.7, 127.5, 121.9, 111.6, 42.6, 40.6, 33.6, 14.5, 13.0; GC-MS (EI): *m*/*z* = 361 (M^+^, 17), 261 (14), 105 (21), 100 (100), 77 (18); HRMS (ESI-TOF) *m*/*z*: [M + Na]^+^ Calcd. for C_23_H_23_NNaO_3_^+^ 384.1570; Found 384.1580.

#### 3.3.11. 2-(4-Benzoyl-3,5-diphenylfuran-2-yl)-*N*,*N*-diethylacetamide (**3fa**)

Yield: 235 mg, starting from 338 mg of **1f** (54%) (Table 1, entry 19). Colorless oil. IR (film): ν = 1647 (s), 1597 (w), 1489 (w), 1447 (m), 1381 (w), 1335 (w), 1254 (m), 1126 (w), 1077 (w), 899 (m), 733 (m), 694 (m) cm^−1^; ^1^H NMR (500 MHz, CDCl_3_): δ = 7.85–7.77 (m, 2 H), 7.62–7.56 (m, 2 H), 7.41–7.10 (m, 11 H), 3.80 (s, 2 H), 3.46 (q, *J* = 7.1, 2 H), 3.35 (q, *J* = 7.1, 2 H), 1.18 (t, *J* = 7.1. 3 H), 1.15 (t, *J* = 7.1, 3 H); ^13^C NMR (125 MHz, CDCl_3_): δ = 193.6, 167.6, 151.9, 145.5, 137.3, 133.3, 131.6, 129.90, 129.71, 129.3, 128.43, 128.36, 128.25, 127.4, 126.5, 125.7, 121.8, 42.4, 40.7, 32.1, 14.3, 13.1; GC-MS (EI): *m*/*z* = 437 (M^+^, 40), 337 (67), 259 (2), 202 (3), 105 (100), 100 (77), 72 (31); HRMS (ESI-TOF) *m*/*z*: [M + Na]^+^ Calcd. for C_29_H_27_NNaO_3_^+^ 460.1883; Found 460.1901.

#### 3.3.12. 2-(4-Benzoyl-5-methylfuran-2-yl)-1-morpholinoethan-1-one (**3gf**)

Yield: 150 mg, starting from 200 mg of **1g** (48%) (Table 1, entry 20). Colorless oil. IR (film): ν = 1647 (s), 1566 (m), 1447 (m), 1273 (w), 1231 (m), 1115 (m), 1038 (w), 903 (m), 729 (m) cm^−1^; ^1^H NMR (500 MHz, CDCl_3_): δ = 7.81–7.75 (m, 2 H), 7.58–7.52 (m, 1 H), 7.49–7.43 (m, 2 H), 6.41 (s, 1 H), 3.72 (s, 2 H), 3.71–3.63 (m, 6 H), 3.57–3.53 (m, 2 H), 2.50 (s, 3 H); ^13^C NMR (125 MHz, CDCl_3_): δ = 191.1, 166.8, 159.0, 146.4, 139.1, 132.2, 128.9, 128.4, 121.4, 109.9, 66.8, 66.6, 46.7, 42.4, 33.3, 14.2; GC-MS (EI): *m*/*z* = 313 (M^+^, 35), 270 (2), 199 (42), 114 (100), 105 (11), 70 (55); HRMS (ESI-TOF) *m*/*z*: [M + Na]^+^ Calcd. for C_18_H_19_NNaO_4_^+^ 336.1206; Found 336.1209.

#### 3.3.13. 2-(4-Acetyl-5-phenylfuran-2-yl)-1-morpholinoethan-1-one (**3gf′**)

Yield: 78 mg, starting from 200 mg of **1g** (25%) (Table 1, entry 20). Colorless oil. IR (film): ν = 1651 (s), 1543 (w), 1447 (m), 1381 (w), 1273 (w), 1234 (m), 1115 (s) cm^−1^; ^1^H NMR (500 MHz, CDCl_3_): δ = 7.87–7.80 (m, 2 H), 7.48–7.40 (m, 3 H), 6.64 (t, br, *J* = 0.7, 1 H), 3.80 (d, *J* = 0.7, 2 H), 3.73–3.64 (m, 6 H), 3.60–3.56 (m, 2 H), 2.38 (s, 3 H); ^13^C NMR (125 MHz, CDCl_3_): δ = 193.8, 166.6, 156.1, 147.8, 129.8, 129.7, 128.5, 128.3, 123.2, 110.5, 66.8, 66.6, 46.7, 42.4, 33.3, 29.8; GC-MS (EI): *m*/*z* = 313 (M^+^, 45), 199 (100), 181 (6), 157 (5), 128 (14), 114 (98), 105 (13), 70 (61); HRMS (ESI-TOF) *m*/*z*: [M + Na]^+^ Calcd. for C_18_H_19_NNaO_4_^+^ 336.1206; Found 336.1217.

#### 3.3.14. Methyl 5-(2-(Diethylamino)-2-oxoethyl)-2-methylfuran-3-carboxylate (**3ha**)

Yield: 177 mg, starting from 154 mg of **1h** (70%) (Table 1, entry 21). Yellow oil. IR (film): ν = 1713 (s), 1651 (s), 1582 (w), 1451 (m), 1396 (w), 1219 (m), 1088 (m), 995 (w), 779 (m) cm^−1^; ^1^H NMR (500 MHz, CDCl_3_): δ = 6.43 (s, 1 H), 3.79 (s, 3 H), 3.65 (s, 2 H), 3.40 (q, *J* = 7.1, 2 H), 3.36 (q, *J* = 7.1, 2 H), 2.54 (s, 3 H), 1.18 (t, *J* = 7.1), 1.14 (t, *J* = 7.1, 3 H); ^13^C NMR (125 MHz, CDCl_3_): δ = 167.3, 164.5, 158.7, 147.3, 114.1, 108.4, 51.2, 42.5, 40.5, 33.5, 14.3, 13.7, 12.9; GC-MS (EI): *m*/*z* = 253 (M^+^, 9), 222 (5), 153 (4), 121 (12), 100 (100), 72 (55); HRMS (ESI-TOF) *m*/*z*: [M + Na]^+^ Calcd. for C_13_H_19_NNaO_4_^+^ 276.1206; Found 276.1218.

#### 3.3.15. Ethyl 5-(2-(Diethylamino)-2-oxoethyl)-2-methylfuran-3-carboxylate (**3ia**)

Yield: 184 mg, starting from 168 mg of **1i** (69%) (Table 1, entry 23). Colorless oil. IR (film): ν = 1713 (s), 1647 (s), 1578 (w), 1466 (m), 1381 (m), 1215 (s), 1099 (w), 1061 (m), 783 (m) cm^−1^; ^1^H NMR (500 MHz, CDCl_3_): δ = 6.43 (s, 1 H), 4.26 (q, *J* = 7.1), 3.64 (s, 2 H), 3.45–3.32 (m, 4 H), 2.53 (s, 3 H), 1.32 (t, *J* = 7.1. 3 H), 1.18 (t, *J* = 7.1, 3 H), 1.14 (t, *J* = 7.1, 3 H); ^13^C NMR (125 MHz, CDCl_3_): δ = 167.4, 164.1, 158.4, 147.3, 114.6, 108.5, 60.0, 42.5, 40.5, 33.5, 14.38, 14.36, 13.7, 12.9; GC-MS (EI): *m*/*z* = 267 (M^+^, 30), 222 (23), 167 (10), 139 (10), 121 (22), 100 (100), 72 (99); HRMS (ESI-TOF) *m*/*z*: [M + Na]^+^ Calcd. for C_14_H_21_NNaO_4_^+^ 290.1363; Found 290.1368.

#### 3.3.16. Ethyl 5-(2-(Diethylamino)-2-oxoethyl)-2-propylfuran-3-carboxylate (**3ja**)

Yield: 200 mg, starting from 196 mg of **1j** (68%) (Table 1, entry 25). Colorless oil. IR (film): ν = 1713 (s), 1647 (s), 1578 (w), 1462 (m), 1431 (m), 1381 (w), 1215 (m), 1096 (m), 1050 (m), 779 (m) cm^−1^; ^1^H NMR (500 MHz, CDCl_3_): δ = 6.45 (s, br, 1 H), 4.26 (q, *J* = 7.1, 2 H), 3.66 (d, *J* = 0.6, 2 H), 3.42–3.34 (m, 4 H), 2.93 (t, *J* = 7.4, 2 H), 1.68 (sextuplet, *J* = 7.4, 2 H), 1.32 (t, *J* = 7.1, 3 H), 1.17 (t, *J* = 7.1, 3 H), 1.13 (t, *J* = 7.1, 3 H), 0.94 (t, *J* = 7.4, 3 H); ^13^C NMR (125 MHz, CDCl_3_): δ = 167.3, 164.0, 162.4, 147.2, 114.2, 108.3, 60.0, 42.5, 40.4, 33.6, 29.5, 21.6, 14.3, 13.8, 13.7, 12.9; GC-MS (EI): *m*/*z* = 295 (M^+^, 6), 250 (3), 195 (3), 100 (100), 72 (37); HRMS (ESI-TOF) *m*/*z*: [M + Na]^+^ Calcd. for C_16_H_25_NNaO_4_^+^ 318.1676; Found 318.1683.

#### 3.3.17. Ethyl 5-(2-(Diethylamino)-2-oxoethyl)-2-isopropylfuran-3-carboxylate (**3ka**)

Yield: 209 mg, starting from 196 mg of **1k** (71%) (Table 1, entry 27). Colorless oil. IR (film): ν = 1713 (s), 1647 (s), 1578 (w), 1381 (m), 1458 (m), 1365 (w), 1215 (m), 1130 (w), 1099 (w), 1061 (m), 783 (m) cm^−1^; ^1^H NMR (500 MHz, CDCl_3_): δ = 6.44 (s, 1 H), 4.26 (q, *J* = 7.1, 2 H), 3.73 (heptuplet, *J* = 7.0, 1 H), 3.67 (s, 2H), 3.45–3.35 (m, 4 H), 1.32 (t, *J* = 7.1. 3 H), 1.25 (d, *J* = 7.0, 6 H), 1.17 (t, *J* = 7.1, 3 H), 1.14 (t, *J* = 7.1, 3 H); ^13^C NMR (125 MHz, CDCl_3_): δ = 167.3, 166.4, 164.0, 146.9, 112.5, 108.2, 59.9, 42.5, 40.4, 33.7, 27.2, 20.78, 20.75, 14.3, 12.9; GC-MS (EI): *m*/*z* = 295 (M^+^, 7), 250 (4), 195 (3), 125 (2), 100 (100), 72 (38); HRMS (ESI-TOF) *m*/*z*: [M + Na]^+^ Calcd. for C_16_H_25_NNaO_4_^+^ 318.1676; Found 318.1675.

#### 3.3.18. Benzyl 5-(2-(Diethylamino)-2-oxoethyl)-2-methylfuran-3-carboxylate (**3la**)

Yield: 220 mg, starting from 230 mg of **1l** (67%) (Table 1, entry 29). Colorless oil. IR (film): ν = 1713 (s), 1647 (s), 1585 (w), 1454 (m), 1431 (m), 1381 (w), 1365 (w), 1219 (m), 1076 (m) cm^−1^; ^1^H NMR (500 MHz, CDCl_3_): δ = 7.41–7.29 (m, 5 H), 6.47 (s, 1 H), 5.25 (s, 2 H), 3.64 (s, 2 H), 3.39 (q, *J* = 7.1, 2 H), 3.35 (q, *J* = 7.1, 2 H), 2.55 (s, 3 H), 1.17 (t, *J* = 7.1, 3 H), 1.13 (t, *J* = 7.1, 3 H); ^13^C NMR (125 MHz, CDCl_3_): δ = 167.4, 163.9, 159.0, 147.4, 136.4, 128.6, 128.2, 128.1, 114.2, 108.6, 65.9, 42.5, 40.5, 33.5, 14.4, 14.0, 13.0; GC-MS (EI): *m*/*z* = 329 (M^+^, 5), 212 (6), 194 (2), 100 (100), 91 (18), 72 (35); HRMS (ESI-TOF) *m*/*z*: [M + Na]^+^ Calcd. for C_19_H_23_NNaO_4_^+^ 352.1519; Found 352.1543.

#### 3.3.19. 5-(2-(Diethylamino)-2-oxoethyl)-*N*,*N*-diethyl-2-methylfuran-3-carboxamide (**3ma**)

Yield: 168 mg, starting from 195 mg of **1m** (57%) (Table 1, entry 31). Colorless oil, IR (film): ν = 1632 (s), 1454 (m), 1381 (w), 1362 (w), 1269 (w), 1072 (w), cm^−1^; ^1^H NMR (300 MHz, CDCl_3_): δ = 6.13 (s, 1 H), 3.66 (s, 2 H), 3.48–3.32 (m, 8 H), 2.34 (s, 3 H), 1.23–1.10 (m, 12 H); ^13^C NMR (125 MHz, CDCl_3_): δ = 167.5, 165.8, 152.0, 146.9, 117.4, 107.4, 42.5, 40.3, 39.2, 33.7, 14.3, 12.9; GC-MS (EI): *m*/*z* = 294 (M^+^, 10), 222 (5), 194 (6), 152 (7), 123 (12), 100 (100), 72 (56); HRMS (ESI-TOF) *m*/*z*: [M + Na]^+^ Calcd. for C_16_H_26_N_2_NaO_3_^+^ 317.1836; Found 317.1837.

#### 3.3.20. *N*,*N*-Dibutyl-2-(5-phenyl-4-tosylfuran-2-yl)acetamide (**3nc**)

Yield: 336 mg, starting from 312 mg of **1n** (72%) (Table 1, entry 32). Yellow oil. IR (film): ν = 1643 (s), 1597 (w), 1551 (w), 1485 (m), 1454 (m), 1319 (s), 1254 (w), 1215 (w), 1153 (s), 1103 (m), 930 (w), 814 (w), 698 (m) cm^−1^; ^1^H NMR (500 MHz, CDCl_3_): δ = 7.85–7.81 (m, 2 H), 7.69–7.65 (m, 2 H), 7.42–7.38 (m, 3 H), 7.20–7.16 (m, 2 H), 6.64 (t,* J* = 0.8, 1 H), 3.73 (d, *J* = 0.8, 2 H), 3.36–3.31 (m, 2 H), 3.28–3.24 (m, 2 H), 2.35 (s, 3 H), 1.59–1.48 (m, 4 H), 1.36–1.22 (m, 4 H), 0.93 (t, *J* = 7.4, 3 H), 0.91 (t, *J* = 7.4, 3 H); ^13^C NMR (125 MHz, CDCl_3_): δ = 166.8, 154.1, 148.9, 144.1, 139.0, 129.8, 129.6, 128.7, 128.4, 128.2, 127.2, 110.3, 48.3, 46.1, 33.4, 31.3, 29.7, 21.5, 20.2, 20.1, 13.8; GC-MS (EI): *m*/*z* = 467 (M^+^, 10), 311 (9), 253 (2), 207 (8), 156 (100), 128 (11), 100 (39), 57 (68); HRMS (ESI-TOF) *m*/*z*: [M + Na]^+^ Calcd. for C_27_H_33_NNaO_4_S^+^ 490.2023; Found 490.2037.

### 3.4. Synthesis of 2-(4-Benzoyl-5-phenylfuran-2-yl)-N,N-diethylacetamide (**3ea**) in Larger Scale

A 250 mL stainless steel autoclave was charged in the presence of air with PdI_2_ (8.6 mg, 2.4 × 10^−2^ mmol), KI (398 mg, 2.4 mmol), a solution of 1,3-diphenyl-2-(prop-2-yn-1-yl)propane-1,3-dione **1e** (627 mg, 2.4 mmol) in anhydrous CH_3_CN (12 mL), and diethylamine **2a** (700 mg, 9.6 mmol). The autoclave was sealed and, while the mixture was stirred, the autoclave was pressurized with CO (16 atm) and air (up to 20 atm). After being stirred at 100 °C for 15 h, the autoclave was cooled, degassed, and opened. After evaporation of the solvent, product **3ea** were purified by column chromatography on silica gel using as eluent hexane-AcOEt from 8:2 to 6:4 (yield: 586 mg, 68%).

## 4. Conclusions

In conclusion, we have reported the synthesis of a previously unreported subclass of furan derivatives (2-(4-acylfuran-2-yl)acetamides) by a direct catalytic carbonylative approach starting from readily available building blocks (2-propargyl-1,3-dicarbonyl compounds, secondary amines, and oxygen). The process is catalyzed by the simple PdI_2_/KI catalytic system and takes place through an ordered sequence of steps, involving: PdI_2_/KI-catalyzed oxidative monaminocarbonylation of the terminal triple bond of the substrate to give the corresponding 2-ynamide intermediate; base-induced enolization and 5-*exo*-*dig O*-cyclization via intramolecular conjugate addition to the 2-ynamide moiety; double bond shift with aromative isomerization.

## Figures and Tables

**Table 1:** PdI_2_-catalyzed oxidative aminocarbonylation of 3-(prop-2-yn-1-yl)pentane-2,4-dione **1a** under different conditions ^a^.

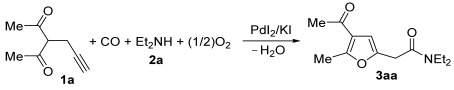								
Entry	Solvent	KI (Equiv)	2a (Equiv)	Substrate Concn. ^b^	T (°C)	P_CO_ (atm)	P_air_ (atm)	Yield of 3aa (%) ^c^
1	MeCN	1	3	0.10	100	16	4	61
2	dioxane	1	3	0.10	100	16	4	56
3	DMA ^d^	1	3	0.10	100	16	4	41
4	MeCN	0.5	3	0.10	100	16	4	45
5	MeCN	1	2	0.10	100	16	4	29
6	MeCN	1	4	0.10	100	16	4	68
7	MeCN	1	5	0.10	100	16	4	66
8	MeCN	1	3	0.20	100	16	4	63
9	MeCN	1	3	0.05	100	16	4	51
10	MeCN	1	3	0.10	100	32	8	53
11 ^e^	MeCN	1	3	0.10	80	16	4	54
12	MeCN	1	4	0.20	100	16	4	72
13 ^f^	MeCN	1	4	0.20	100	16	4	55

^a^ Unless otherwise noted, all reactions were carried out in 15 h, using 1 mol% of PdI_2_. Substrate conversion was quantitative in all cases. ^b^ Mmol of starting **1a** per mL of solvent. ^c^ Isolated yield based on starting **1a**. ^d^ DMA = *N*,*N*-dimethylacetamide. ^e^ The GLC analysis showed the presence of trace of substrate. ^f^ The reaction was carried out using 0.33 mol% of PdI_2_.

**Table 2:** Synthesis of 2-(4-acylfuran-2-yl)acetamides **3** by PdI_2_/KI-catalyzed oxidative aminocarbonylation of 2-propargyl-1,3-dicarbonyl compounds **1** with secondary amines **2** ^a^.

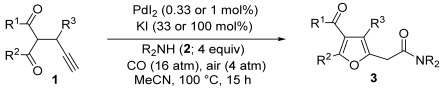					
Entry	PdI_2_ (mol%)	1	2	3	Yield of 3 (%) ^b^
1	1	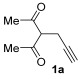	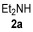	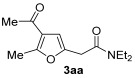	72
2	0.33	**1a**	**2a**	**3aa**	55
3	1	**1a**	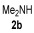	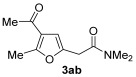	67
4	0.33	**1a**	**2b**	**3ab**	55
5	1	**1a**	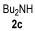	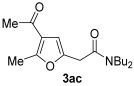	75
6	0.33	**1a**	**2c**	**3ac**	58
7	1	**1a**	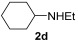	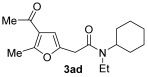	74
8	0.33	**1a**	**2d**	**3ad**	59
9	1	**1a**	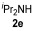	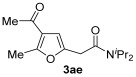	54
10	1	**1a**	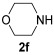	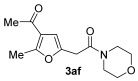	74
11	0.33	**1a**	**2f**	**3af**	61
12	1	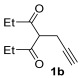	**2a**	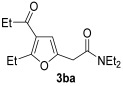	81
13	0.33	**1b**	**2a**	**3ba**	68
14	1	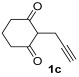	**2a**	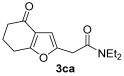	67
15	0.33	**1c**	**2a**	**3ca**	58
16	1	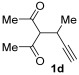	**2f**	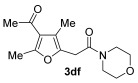	66
17	1	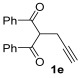	**2a**	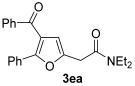	68
18	0.33	**1e**	**2a**	**3ea**	62
19	1	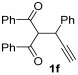	**2a**	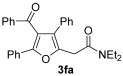	54
20	1	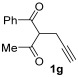	**2f**	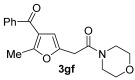	48
				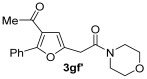	25
21	1	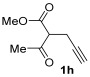	**2a**	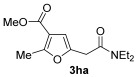	70
22	0.33	**1h**	**2a**	**3ha**	58
23	1	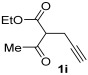	**2a**	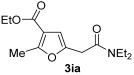	69
24	0.33	**1i**	**2a**	**3ia**	58
25	1	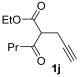	**2a**	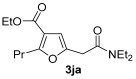	68
26	0.33	**1j**	**2a**	**3ja**	63
27	1	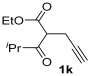	**2a**	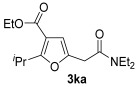	71
28	0.33	**1k**	**2a**	**3ka**	60
29	1	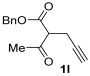	**2a**	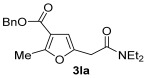	67
30	0.33	**1l**	**2a**	**3la**	56
31	1	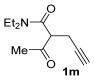	**2a**	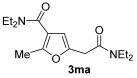	57
32	1	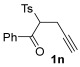	**2c**	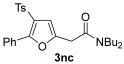	72
33	0.33	**1n**	**2c**	**3nc**	67

^a^ All reactions were carried out in MeCN as the solvent (0.20 mmol of **1** per mL of MeCN), in the presence of PdI_2_ (1 mol% or 0.33 mol%), KI (KI:PdI_2_ molar ratio = 100), and amine **2** (4 equiv) for 15 h, under 20 atm of a 4:1 mixture of CO–air. Substrate conversion was quantitative in all cases. ^b^ Isolated yield based on starting **1**.

## Data Availability

The data presented in this study are available on request from the corresponding authors.

## Supplementary Materials

The following supporting information can be downloaded at: https://www.mdpi.com/article/10.3390/molecules28196764/s1, Preparation and characterization of substrates, Copies of HRMS, ^1^H NMR, and ^13^C NMR spectra for substrates **1b**, **1d**, **1k**, **1m** and products **3aa**, **3ab**, **3ac**, **3ad**, **3ae**, **3af**, **3ba**, **3ca**, **3df**, **3ea**, **3fa**, **3gf**, **3gf′**, **3ha**, **3ia**, **3ja**, **3ka**, **3la**, **3ma**, **3nc**. References [33,34,35,36,37,38,39,40,41] are cited in Supplementary Materials.

## Sample Availability

Samples of the compounds **3aa**, **3ab**, **3ac**, **3ad**, **3ae**, **3af**, **3ba**, **3ca**, **3df**, **3ea**, **3fa**, **3gf**, **3gf′**, **3ha**, **3ia**, **3ja**, **3ka**, **3la**, **3ma**, and **3nc** are available from the authors.
