# Supplementary material for: Palladium Iodide Catalyzed Multicomponent Carbonylative Synthesis of 2-(4-Acylfuran-2-yl)acetamides
Source: Molecules. 2023 Sep 22;28(19):6764. doi: 10.3390/molecules28196764 (PMC10574086; doi:10.3390/molecules28196764)
Supplement: Supplementary file 1 [file molecules-28-06764-s001.zip › molecules-2612560-supplementary.pdf]

Supporting Information for

Palladium Iodide Catalyzed Multicomponent Carbonylative Synthesis of 2-(4-Acylfuran-2-yl)acetamides

Ida Ziccarelli [1], Lucia Veltri [1,*], Tommaso Prestia [1], Roberta Amuso [1], Maria A. Chiacchio [2], Raffaella Mancuso [1] and Bartolo Gabriele [1,*]

[1] Laboratory of Industrial and Synthetic Organic Chemistry (LISOC), Department of Chemistry and Chemical Technologies, University of Calabria, Via Pietro Bucci 12/C, 87036 Arcavacata di Rende (CS), Italy;

[2] Department of Drug Sciences, University of Catania, Viale A. Doria 6, Catania, 95125, Italy;

* Correspondence: lucia.veltri@unical.it (L.V.); bartolo.gabriele@unical.it (B.G.)

Table of Contents

Page S2 –S3 **Preparation and Characterization of Substrates**

Page S4–S15 **Copies of HRMS Spectra**

Pages S16−S63 **Copies of ^{1}H NMR and ^{13}C NMR Spectra**

Page S64 **References**

Preparation and Characterization of Substrates

Substrates **1a** [33], **1c** [34], **1e** [35], **1f** [36], **1g** [37], **1h** [38], **1i** [38], **1j** [39], **1l** [40], and **1n** [34] were prepared as reported in the literature. A similar procedure was employed for the preparation substrates **1b**, **1d**, **1k** and **1m** as described below.

General Procedure for the Preparation of 2-Propynyl-1,3-dicarbonyl substrates 1b, 1k and 1m

To a cooled (0°C), stirred solution of NaH (95% purity, 220 mg, 8.7 mmol) in anhydrous THF (20 mL) was slowly added the 1,3-dicarbonyls derivative under nitrogen (8.7 mmol; heptane-3,5-dione, 1.12 g; ethyl 4-methyl-3-oxopentanoate, 1.38 g; *N,N*-diethyl-3-oxobutanamide [41], 1.37 g). The reaction mixture was allowed to stir under nitrogen at 0°C for 0.5 h. Propargyl bromide (80 wt % solution in toluene, 1.0 mL, 9.3 mmol) was then added dropwise at 0 °C. The resulting mixture was allowed to warm up to room temperature and then stirred for 6 d (for the synthesis of **1b**), 15 h (for **1k**), or 24 h (for **1m**). The reaction mixture was quenched with water (40 mL) and extracted with AcOEt (3 × 50 mL). The combined organic phases were washed with brine (40 mL), dried over anhydrous Na_2SO_4, filtered and concentrated by rotary evaporation. The crude products were purified by column chromatography on silica gel using as eluent 9:1 hexane-AcOEt (**1b**), 7:3 hexane-AcOEt (**1k**), or 1:1 hexane-AcOEt for substrate (**1m**).

3-(Prop-2-yn-1-yl)pentane-2,4-dione (1b): Yield: 850 mg, starting from 1.12 g of heptane-3,5-dione (59%). Colorless oil, IR (KBr): ν = 3287 (m), 2122 (vw), 1728 (s), 1706 (s), 1600 (w), 1458 (w), 1404 (w), 1381 (m), 1234 (w), 1200 (w), 1107 (m), 648 (m) cm^{-1}; ^1H NMR (500 MHz, $CDCl_3$): δ = 3.89 (t, *J* = 7.6, 1 H), 2.70 (dd, *J* = 7.6, 2.7, 2 H), 2.65-2.49 (m, 4 H), 2.02 (t, *J* = 2.7, 1 H), 1.06 (t, *J* = 7.2, 6 H); ^{13}C NMR (125 MHz, $CDCl_3$): δ = 205.0, 80.6, 70.6, 64.9, 35.8, 17.6, 7.5; GC-MS (EI): *m/z* = 166 (M^+, 0.5), 151 (10), 137 (5), 110 (11), 81 (13), 57 (100); HRMS (ESI - TOF) *m/z*: $[M + Na]^+$ Calcd for $C_{10}H_{14}N_2NaO_2^+$ 189.0886; Found: 189.0890.

Ethyl 2-isobutyrylpent-4-ynoate (1k): Yield: 1.04 g, starting from 1.38 g of ethyl 4-methyl-3-oxopentanoate (61%). Colorless oil, IR (KBr): ν = 3287 (m), 2122 (vw), 1744 (s), 1713 (s), 1466 (m), 1369 (w), 1258 (m), 1180 (m), 1099 (m), 1034 (w), 1007 (w), 856 (w), 648 (m) cm^{-1}. ^1H NMR (500 MHz, $CDCl_3$): δ = 4.25-4.16 (m, 2 H), 3.90 (dd, *J* = 7.8, 7.2, 1 H), 2.89 (heptuplet, *J* = 6.9, 1 H), 2.77-2.67 (m, 2 H), 1.99 (t, *J* = 2.6, 1 H), 1.27 (t, *J* = 7.2, 3 H), 1.15 (d, *J* = 6.9, 3H), 1.14 (d, *J* = 6.9, 3H); ^{13}C NMR (125 MHz, $CDCl_3$): δ = 207.3, 168.1, 80.8, 70.2, 61.7, 55.6, 41.0, 18.1, 17.7, 14.1; GC-MS (EI): *m/z* = 196 (M^+, absent), 181 (1), 167 (5), 153 (9), 123 (5), 97 (9), 79 (7), 71 (100); HRMS (ESI - TOF) *m/z*: $[M-H]^-$ Calcd for $C_{11}H_{15}O_3^-$ 195.1027; Found: 195.1023.

2-Acetyl-*N,N*-diethylpent-4-ynamide (1m): Yield: 1.12 g, starting from 1.37 g of *N,N*-diethyl-3-oxobutanamide (66%). Colorless oil, IR (KBr): ν = 3279 (m), 2122 (vw), 1724 (s), 1632 (s), 1454 (m), 1358 (m), 1262 (m), 1215 (m), 1165 (w), 1138 (w), 620 (m) cm^{-1}. ^1H NMR (500 MHz, $CDCl_3$): δ = 3.81-3.76 (m, 1 H), 3.54-3.34 (m, 4 H, 2 CH_2CH_3), 2.85 (ddd, *J* = 17.2, 7.1, 2.7, 1 H), 2.72 (ddd, *J* = 17.2, 7.9, 2.7, 1 H), 2.20 (s, 3 H), 2.02 (t, *J* = 2.7, 1 H), 1.26 (t, *J* = 7.1, 3 H), 1.15 (t, *J* = 7.1, 3 H); ^{13}C NMR (125 MHz, $CDCl_3$): δ = 202.6, 167.2, 80.9, 70.5, 56.0, 42.6, 40.9, 27.1, 18.7, 14.6, 12.8; GC-MS (EI): *m/z* = 195 (M^+, 1), 180 (2), 152 (41), 124 (100), 100 (15), 79 (14), 72 (73), 58 (92); HRMS (ESI - TOF) *m/z*: $[M + Na]^+$ Calcd for $C_{11}H_{17}NNaO_2^+$ 218.1151; Found: 218.1154.

Preparation of 3-(but-3-yn-2-yl)pentane-2,4-dione 1d

To a stirred solution of pentane-2,4-dione (8.10 g, 80.9 mmol) in acetone (150 mL), under nitrogen atmosphere, was added K_2CO_3 (5.6 g, 40.5 mmol) followed by but-3-yn-2-yl methanesulfonate (5.0 g, 33.7 mmol). The mixture was stirred for 2.5 d at 60 °C. After cooling, the mixture was filtered and the solvent

removed by rotary evaporation. The crude product was purified by column chromatography using as eluent 95:5 hexane-AcOEt) to give 3-(but-3-yn-2-yl)pentane-2,4-dione as a colorless liquid (yield: 1.8 g, 35%). IR (KBr): ν = 3306 (m), 2114 (vw), 1732 (s), 1699 (s), 1558 (w), 1423 (w), 1360 (m), 1196 (w), 1157 (m), 752 (m) cm^{-1}; ^1H NMR (500 MHz, $CDCl_3$): δ = 3.74 (d, *J* = 10.1, 1 H), 3.35-3.27 (m, 1 H), 2.25 (s, 3 H), 2.22 (s, 3 H), 2.17-2.13 (m, 1 H), 1.16 (d, *J* = 6.8, 3 H); ^{13}C NMR (125 MHz, $CDCl_3$): δ = 202.2, 201.9, 85.0, 74.3, 70.9, 30.3, 29.0, 25.4, 18.9; GC-MS (EI): *m/z* = 152 (M^+, 1), 137 (6), 109 (75), 95 (100), 79 (11), 67 (23); HRMS (ESI - TOF) *m/z*: $[M + Na]^+$ Calcd for $C_9H_{12}NaO_2^+$ 175.0730; Found: 175.0735.

Copies of HRMS spectra

3-(Prop-2-yn-1-yl)pentane-2,4-dione (1b)

Calcd for $C_{10}H_{14}N_2NaO_2^+$ 189.0886; Found: 189.0890.

**Sample Name**	See data file	**Position**		**Instrument Name**	Instrument 1	**User Name**	QTOF-HP\admin
**Inj Vol**	-1	**InjPosition**		**SampleType**	Sample	**IRM Calibration Status**	Success
**Data Filename**	1B.d	**ACQ Method**	infusione-10uL-min.m	**Comment**		**Acquired Time**	12/6/2022 9:30:57 AM

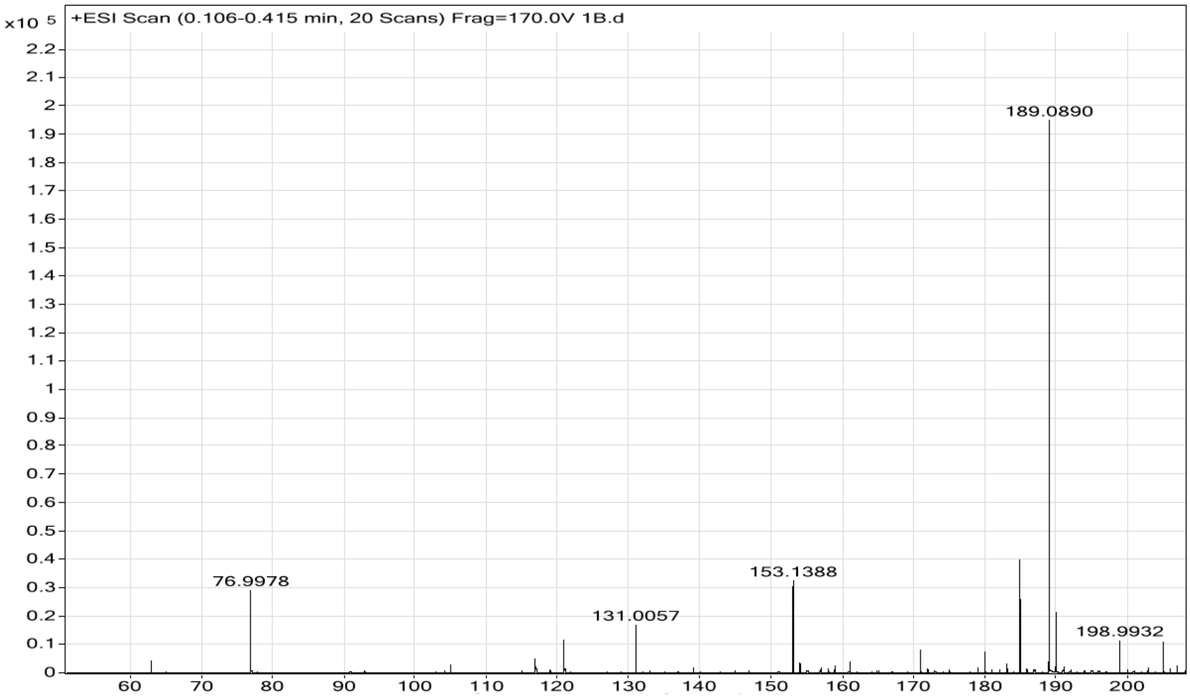

3-(But-3-yn-2-yl)pentane-2,4-dione (1d)

Calcd for $C_9H_{12}NaO_2^+$ 175.0730; Found: 175.0735.

**Sample Name**	See data file	**Position**		**Instrument Name**	Instrument 1	**User Name**	QTOF-HP\admin
**Inj Vol**	-1	**InjPosition**		**SampleType**	Sample	**IRM Calibration Status**	Success
**Data Filename**	1F.d	**ACQ Method**	infusione-10uL-min.m	**Comment**		**Acquired Time**	3/31/2023 9:58:59 AM

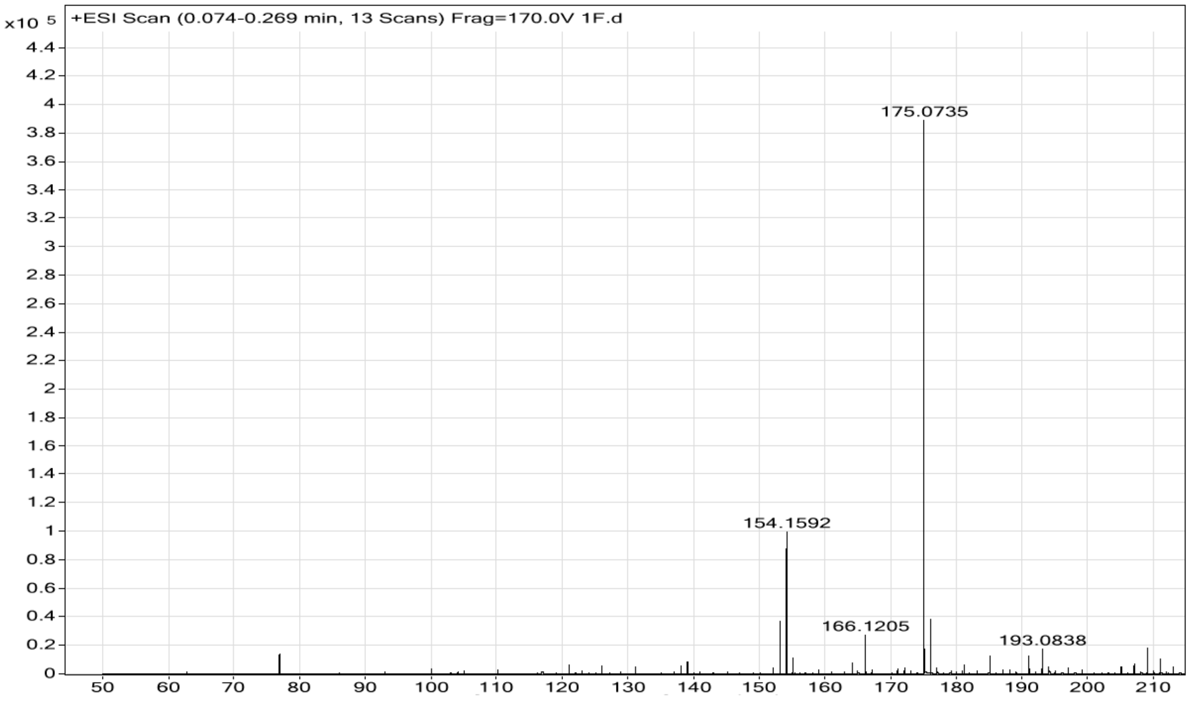

Ethyl 2-isobutyrylpent-4-ynoate (1k)

Calcd for $C_{11}H_{15}O_3^-$ 195.1027; Found: 195.1023.

**Sample Name**	See data file	**Position**		**Instrument Name**	Instrument 1	**User Name**	QTOF-HP\admin
**Inj Vol**	-1	**InjPosition**		**SampleType**	Sample	**IRM Calibration Status**	Success
**Data Filename**	3D-N.d	**ACQ Method**	infusione-10uL-min.m	**Comment**		**Acquired Time**	2/28/2022 10:16:28 AM

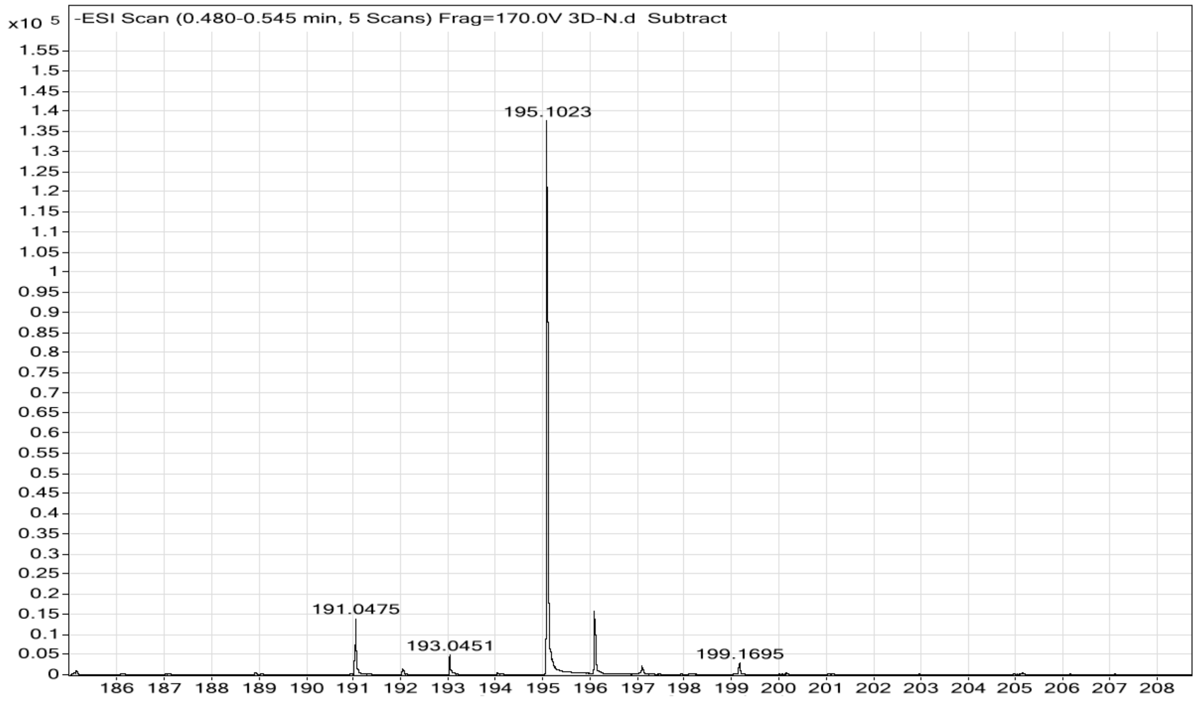

2-Acetyl-*N*,*N*-diethylpent-4-ynamide (1m)

Calcd for $C_{11}H_{17}NNaO_2^+$ 218.1151; Found: 218.1154.

**Sample Name**	See data file	**Position**		**Instrument Name**	Instrument 1	**User Name**	QTOF-HP\admin
**Inj Vol**	-1	**InjPosition**		**SampleType**	Sample	**IRM Calibration Status**	Success
**Data Filename**	3G.d	**ACQ Method**	infusione-10uL-min.m	**Comment**		**Acquired Time**	2/28/2022 10:31:06 AM

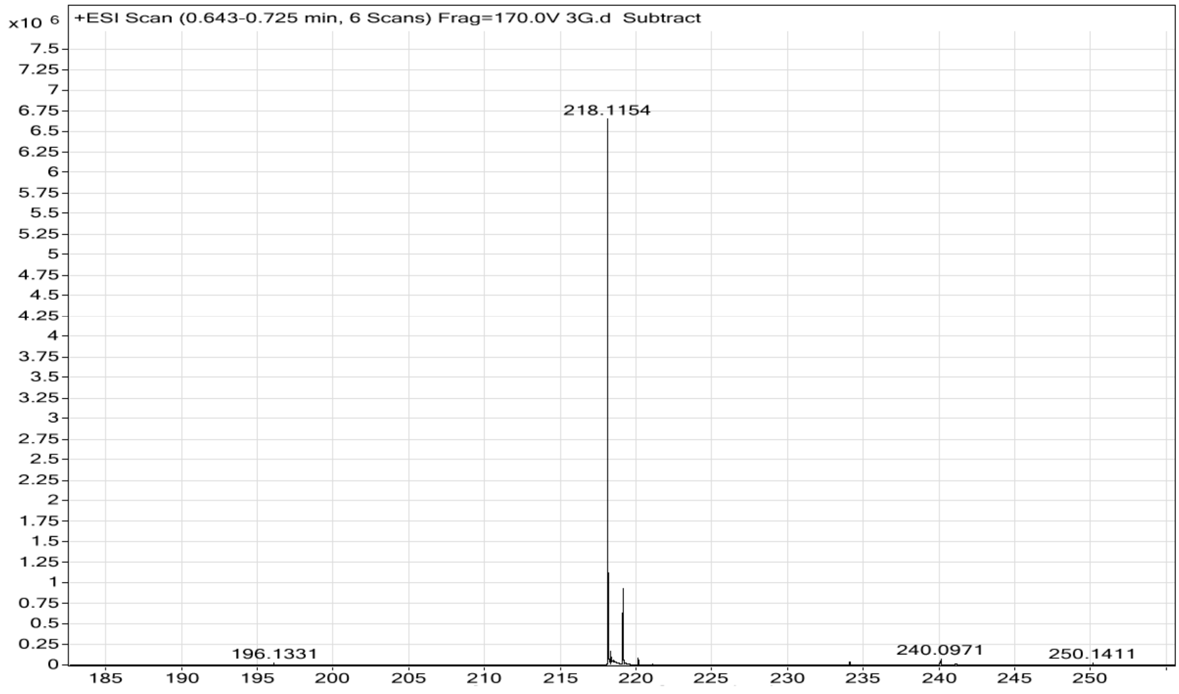

2-(4-Acetyl-5-methylfuran-2-yl)-*N,N*-diethylacetamide (3aa)

Calcd. for $C_{13}H_{19}NNaO_3^+$ 260.1257; Found 260.1256

**Sample Name**	See data file	**Position**		**Instrument Name**	Instrument 1	**User Name**	QTOF-HP\admin
**Inj Vol**	-1	**InjPosition**		**SampleType**	Sample	**IRM Calibration Status**	Success
**Data Filename**	4AA.d	**ACQ Method**	infusione-10uL-min.m	**Comment**		**Acquired Time**	2/28/2022 10:43:28 AM

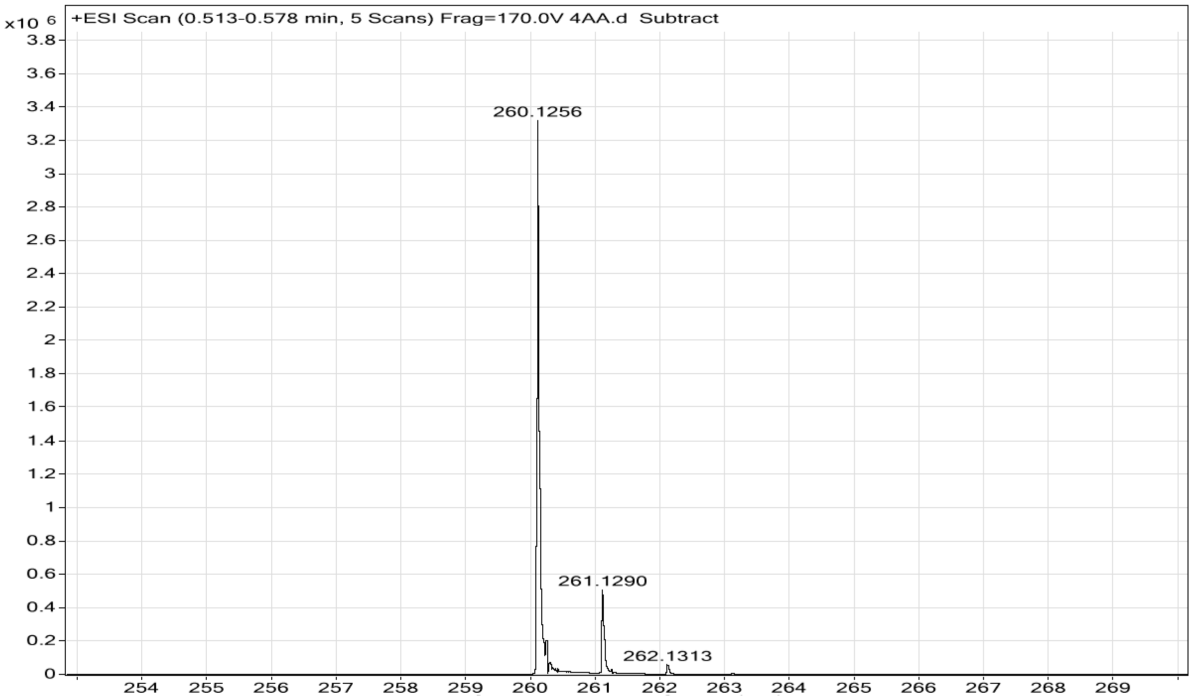

2-(4-Acetyl-5-methylfuran-2-yl)-*N,N*-dimethylacetamide(3ab)

Calcd. for $C_{11}H_{15}NNaO_3{}^+$ 232.0944; Found 232.0948

**Sample Name**	See data file	**Position**		**Instrument Name**	Instrument 1	**User Name**	QTOF-HP\admin
**Inj Vol**	-1	**InjPosition**		**SampleType**	Sample	**IRM Calibration Status**	Success
**Data Filename**	4AD.d	**ACQ Method**	infusione-10uL-min.m	**Comment**		**Acquired Time**	2/28/2022 11:41:50 AM

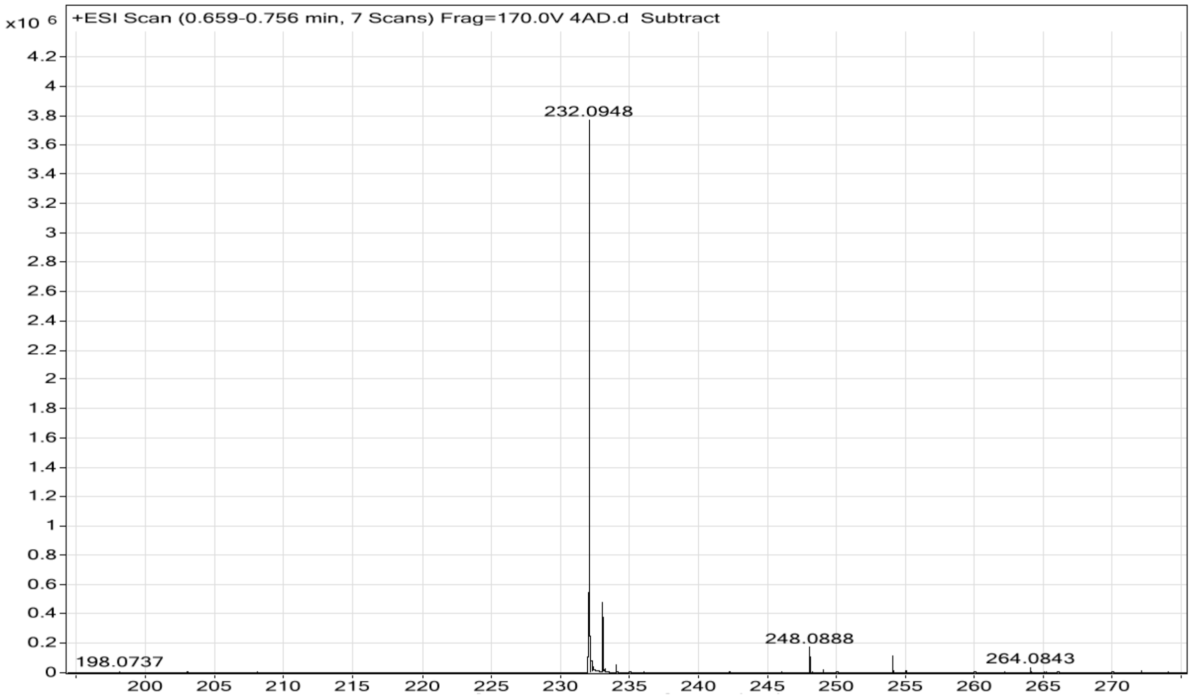

2-(4-Acetyl-5-methylfuran-2-yl)-*N,N*-dibutylacetamide (3ac)

Calcd. for $C_{17}H_{27}NNaO_3^+$ 316.1883; Found 316.1898

**Sample Name**	See data file	**Position**		**Instrument Name**	Instrument 1	**User Name**	QTOF-HP\admin
**Inj Vol**	-1	**InjPosition**		**SampleType**	Sample	**IRM Calibration Status**	Success
**Data Filename**	AAC.d	**ACQ Method**	infusione-10uL-min.m	**Comment**		**Acquired Time**	2/28/2022 11:29:34 AM

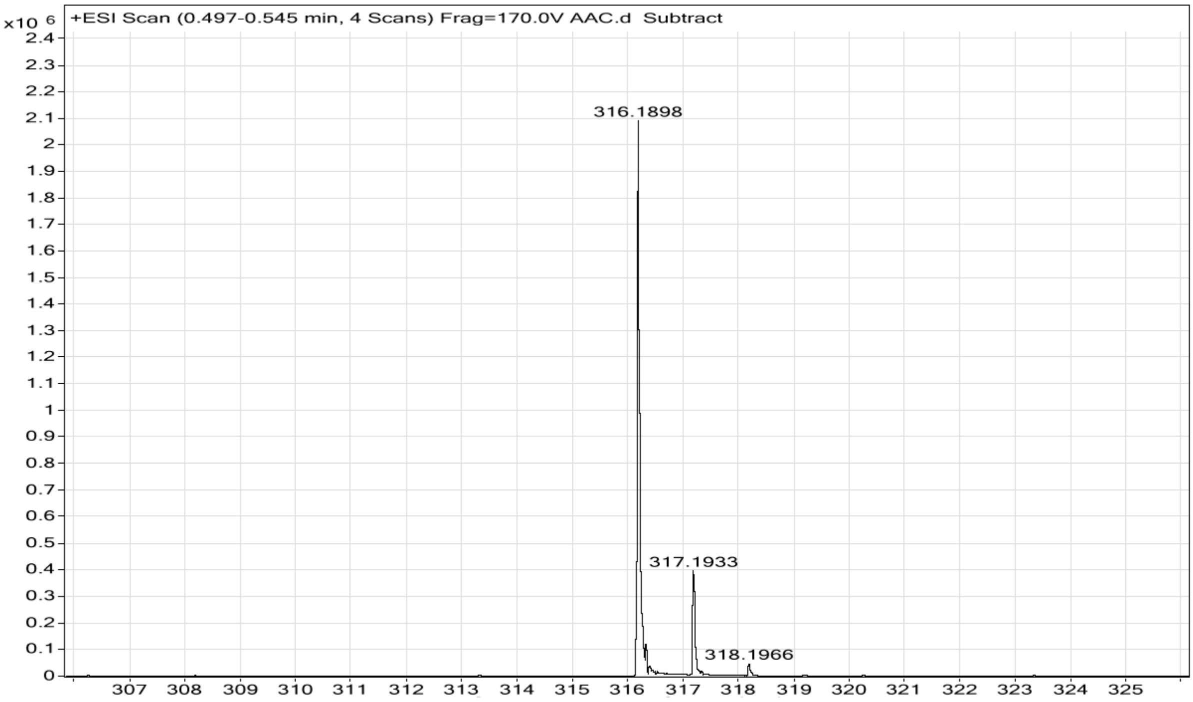

2-(4-Acetyl-5-methylfuran-2-yl)-*N*-*c*yclohexyl-*N*-ethylacetamide (3ad)

Mixture of rotamers A + B, A/B ca 1.2

Calcd. for $C_{17}H_{25}NNaO_3^+$ 314.1727; Found 314.1712

**Sample Name**	See data file	**Position**		**Instrument Name**	Instrument 1	**User Name**	QTOF-HP\admin
**Inj Vol**	-1	**InjPosition**		**SampleType**	Sample	**IRM Calibration Status**	Success
**Data Filename**	4AF.d	**ACQ Method**	infusione-10uL-min.m	**Comment**		**Acquired Time**	2/28/2022 12:06:19 PM

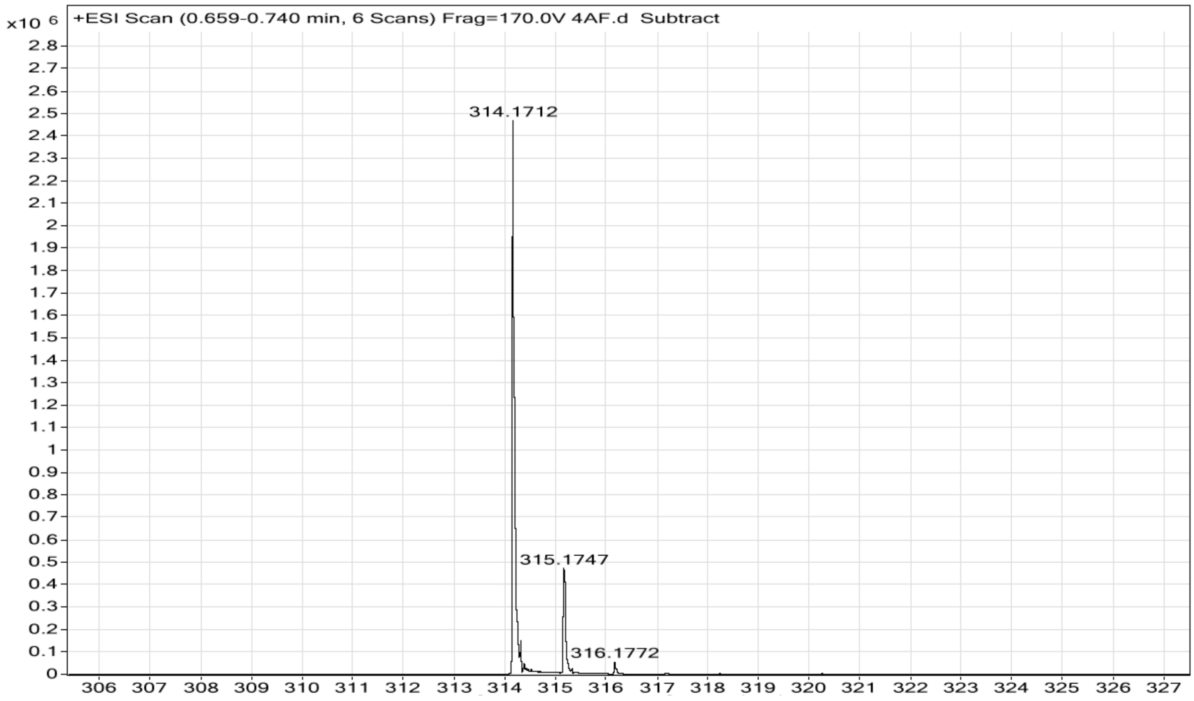

2-(4-Acetyl-5-methylfuran-2-yl)-*N,N*-diisopropylacetamide (3ae)

Calcd. for $C_{15}H_{23}NNaO_3^+$ 288.1570; Found 288.1561

**Sample Name**	See data file	**Position**		**Instrument Name**	Instrument 1	**User Name**	QTOF-HP\admin
**Inj Vol**	-1	**InjPosition**		**SampleType**	Sample	**IRM Calibration Status**	Success
**Data Filename**	4AE.d	**ACQ Method**	infusione-10uL-min.m	**Comment**		**Acquired Time**	2/28/2022 11:55:20 AM

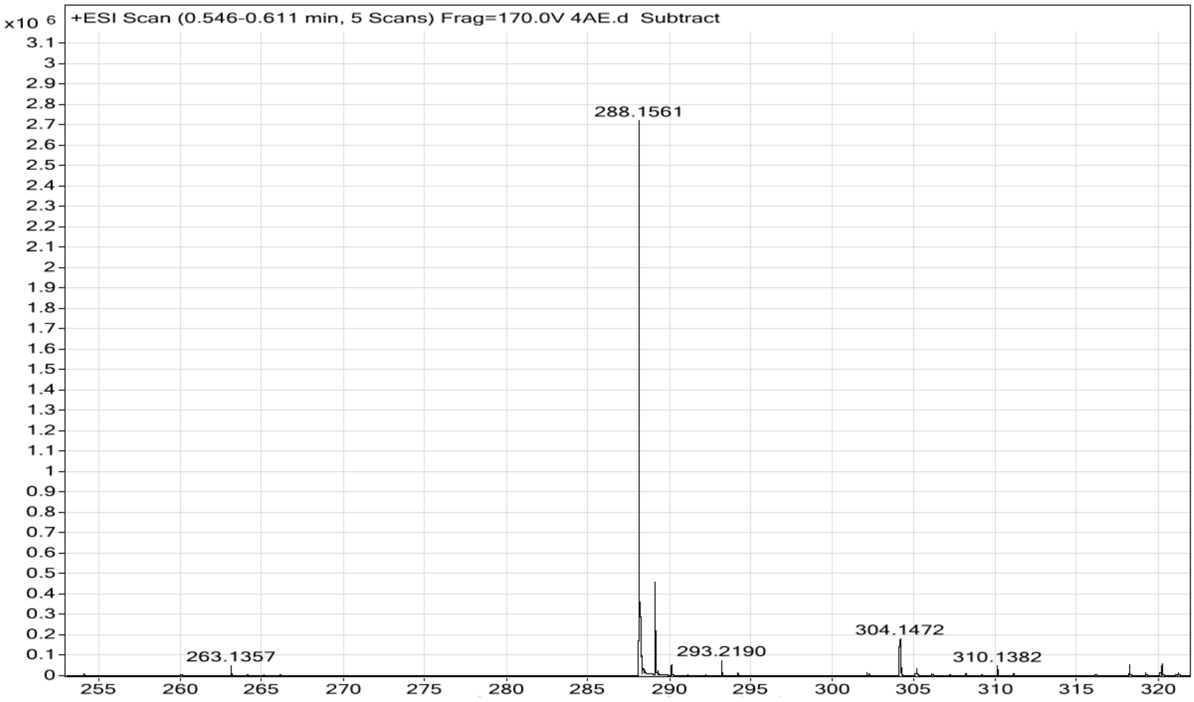

2-(4-Acetyl-5-methylfuran-2-yl)-1-morpholinoethan-1-one (3af)

Calcd. for $C_{13}H_{17}NNaO_4^+$ 274.1050; Found 274.1050.

**Sample Name**	See data file	**Position**		**Instrument Name**	Instrument 1	**User Name**	QTOF-HP\admin
**Inj Vol**	-1	**InjPosition**		**SampleType**	Sample	**IRM Calibration Status**	Success
**Data Filename**	4AB.d	**ACQ Method**	infusione-10uL-min.m	**Comment**		**Acquired Time**	2/28/2022 11:00:37 AM

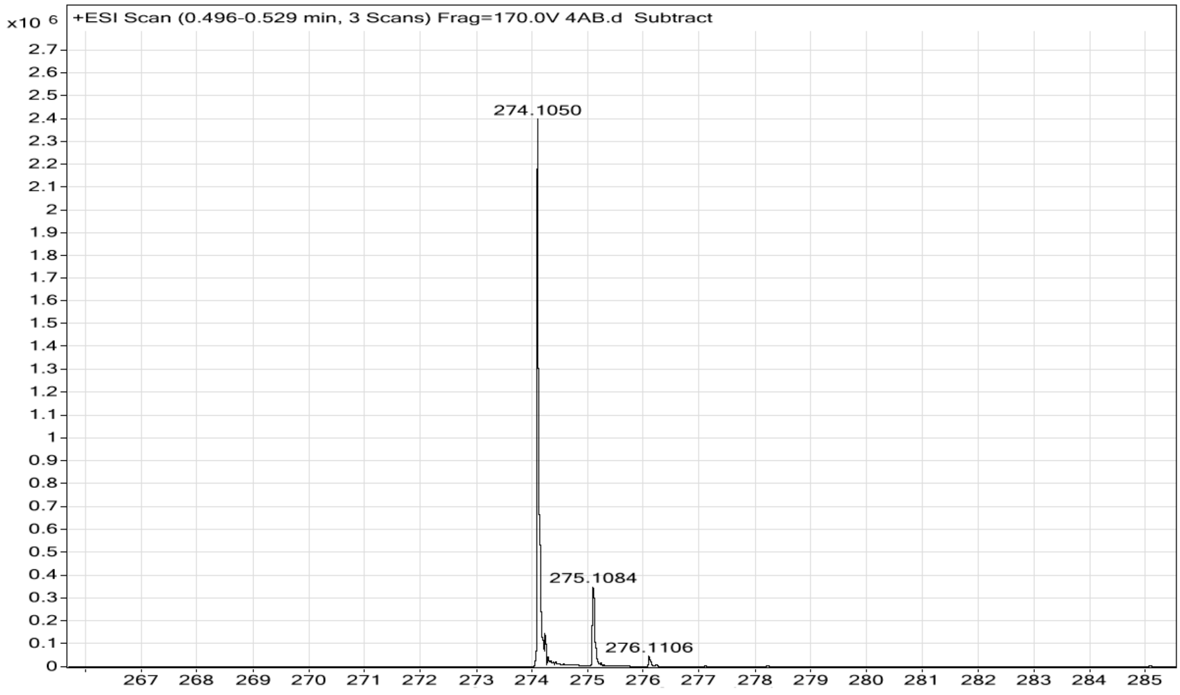

_N,N_-Diethyl-2-(5-ethyl-4-propionylfuran-2-yl)acetamide (3ba)

Calcd. for $C_{15}H_{23}NNaO_3^+$ 288.1570; Found 288.1576

**Sample Name**	See data file	**Position**		**Instrument Name**	Instrument 1	**User Name**	QTOF-HP\admin
**Inj Vol**	-1	**InjPosition**		**SampleType**	Sample	**IRM Calibration Status**	Success
**Data Filename**	3BA.d	**ACQ Method**	infusione-10uL-min.m	**Comment**		**Acquired Time**	12/6/2022 8:48:31 AM

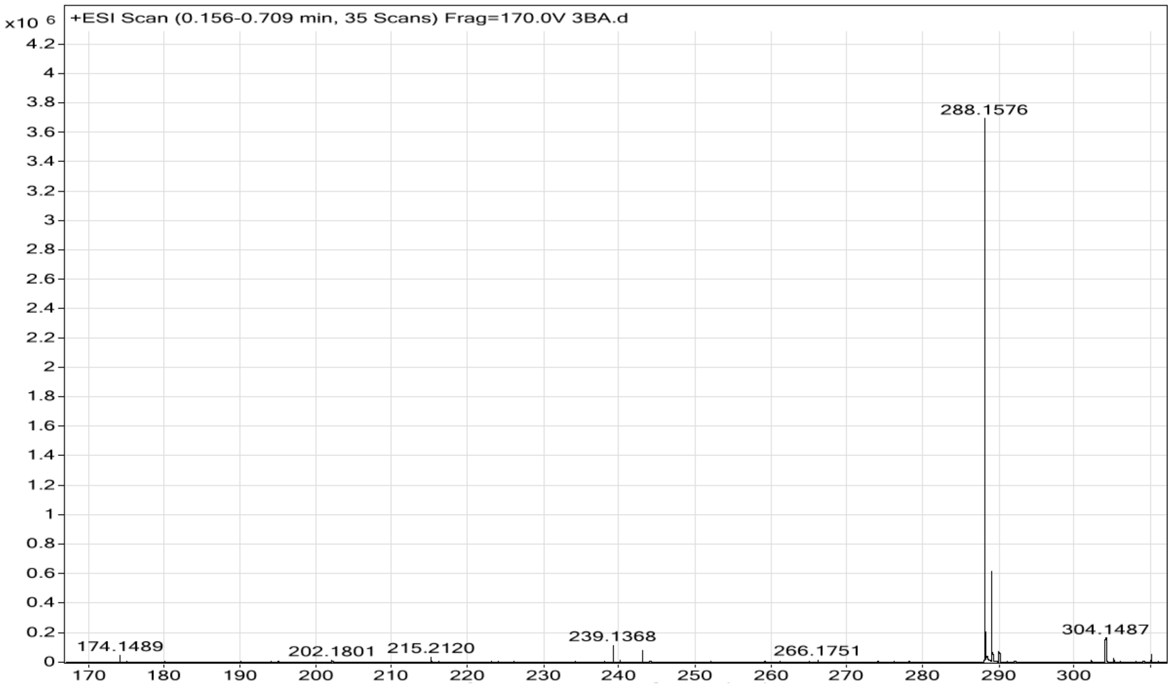

_N,N_-Diethyl-2-(4-oxo-4,5,6,7-tetrahydrobenzofuran-2-yl)acetamide (3ca)

Calcd. for $C_{14}H_{19}NNaO_3^+$ 272.1257; Found 272.1261

**Sample Name**	See data file	**Position**		**Instrument Name**	Instrument 1	**User Name**	QTOF-HP\admin
**Inj Vol**	-1	**InjPosition**		**SampleType**	Sample	**IRM Calibration Status**	Success
**Data Filename**	3DA.d	**ACQ Method**	infusione-10uL-min.m	**Comment**		**Acquired Time**	3/31/2023 8:24:01 AM

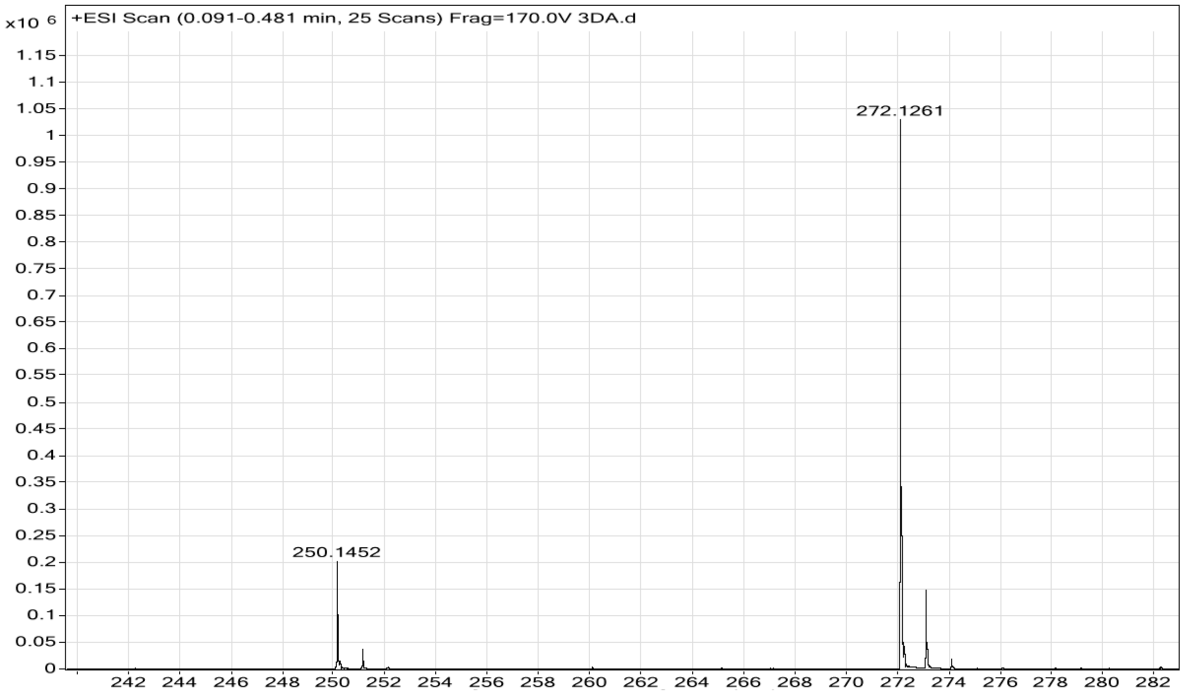

2-(4-Acetyl-3,5-dimethylfuran-2-yl)-1-morpholinoethan-1-one (3df)

Calcd. for $C_{14}H_{19}NNaO_4^+$ 288.1206; Found 288.1216

**Sample Name**	See data file	**Position**		**Instrument Name**	Instrument 1	**User Name**	QTOF-HP\admin
**Inj Vol**	-1	**InjPosition**		**SampleType**	Sample	**IRM Calibration Status**	Success
**Data Filename**	3FB.d	**ACQ Method**	infusione-10uL-min.m	**Comment**		**Acquired Time**	3/31/2023 8:49:54 AM

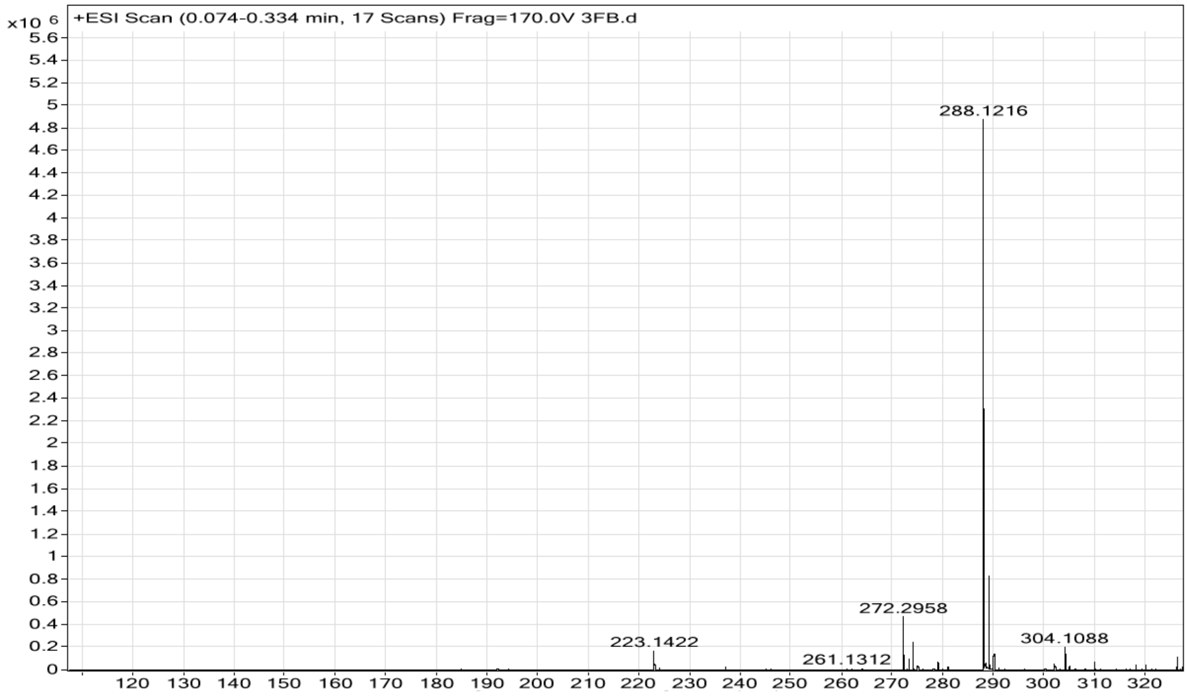

2-(4-Benzoyl-5-phenylfuran-2-yl)-*N,N*-diethylacetamide (3ea)

Calcd. for $C_{23}H_{23}NNaO_3^+$ 384.1570; Found 384.1580

**Sample Name**	See data file	**Position**		**Instrument Name**	Instrument 1	**User Name**	QTOF-HP\admin
**Inj Vol**	-1	**InjPosition**		**SampleType**	Sample	**IRM Calibration Status**	Success
**Data Filename**	4HA.d	**ACQ Method**	infusione-10uL-min.m	**Comment**		**Acquired Time**	2/28/2022 1:03:21 PM

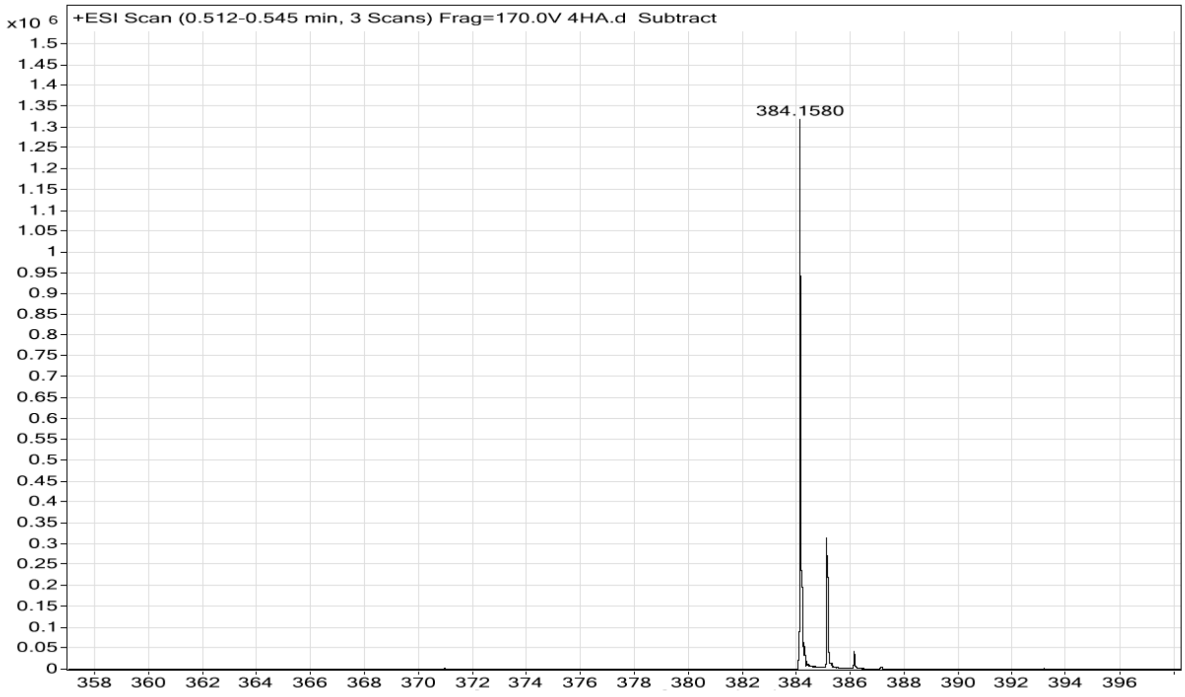

2-(4-Benzoyl-3,5-diphenylfuran-2-yl)-*N,N*-diethylacetamide (3fa)

Calcd. for $C_{29}H_{27}NNaO_3^+$ 460.1883; Found 460.1901

**Sample Name**	See data file	**Position**		**Instrument Name**	Instrument 1	**User Name**	QTOF-HP\admin
**Inj Vol**	-1	**InjPosition**		**SampleType**	Sample	**IRM Calibration Status**	Success
**Data Filename**	3GA.d	**ACQ Method**	infusione-10uL-min.m	**Comment**		**Acquired Time**	3/31/2023 9:10:30 AM

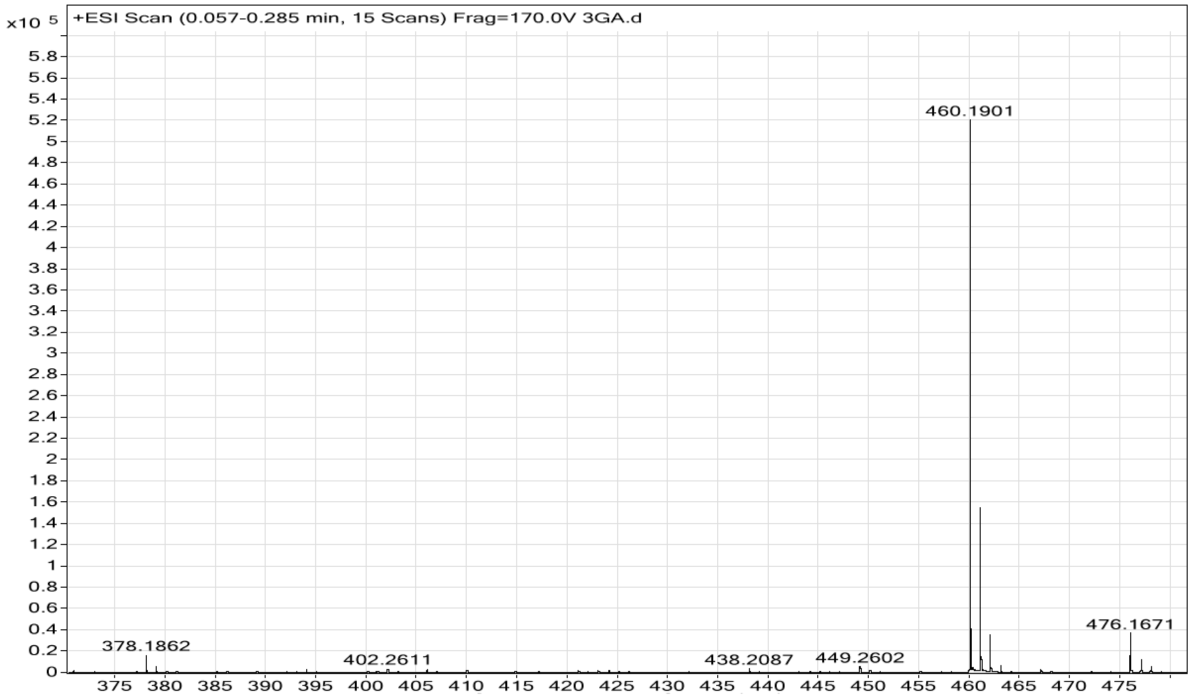

2-(4-Benzoyl-5-methylfuran-2-yl)-1-morpholinoethan-1-one (3gf)

Calcd. for $C_{18}H_{19}NNaO_4^+$ 336.1206; Found 336.1209

**Sample Name**	See data file	**Position**		**Instrument Name**	Instrument 1	**User Name**	QTOF-HP\admin
**Inj Vol**	-1	**InjPosition**		**SampleType**	Sample	**IRM Calibration Status**	Success
**Data Filename**	3EB.d	**ACQ Method**	infusione-10uL-min.m	**Comment**		**Acquired Time**	3/31/2023 8:34:55 AM

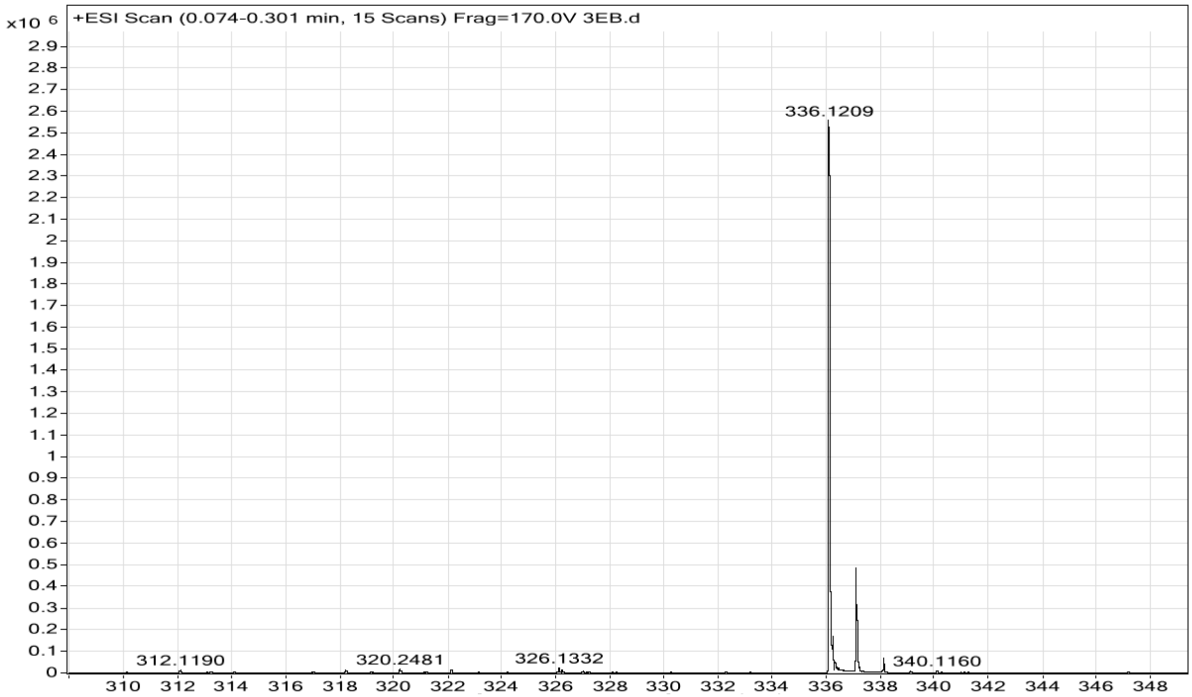

2-(4-Acetyl-5-phenylfuran-2-yl)-1-morpholinoethan-1-one (3gf’)

Calcd. for $C_{18}H_{19}NNaO_4^+$ 336.1206; Found 336.1217

**Sample Name**	See data file	**Position**		**Instrument Name**	Instrument 1	**User Name**	QTOF-HP\admin
**Inj Vol**	-1	**InjPosition**		**SampleType**	Sample	**IRM Calibration Status**	Success
**Data Filename**	3EB-PRIMO.d	**ACQ Method**	infusione-10uL-min.m	**Comment**		**Acquired Time**	3/31/2023 9:00:35 AM

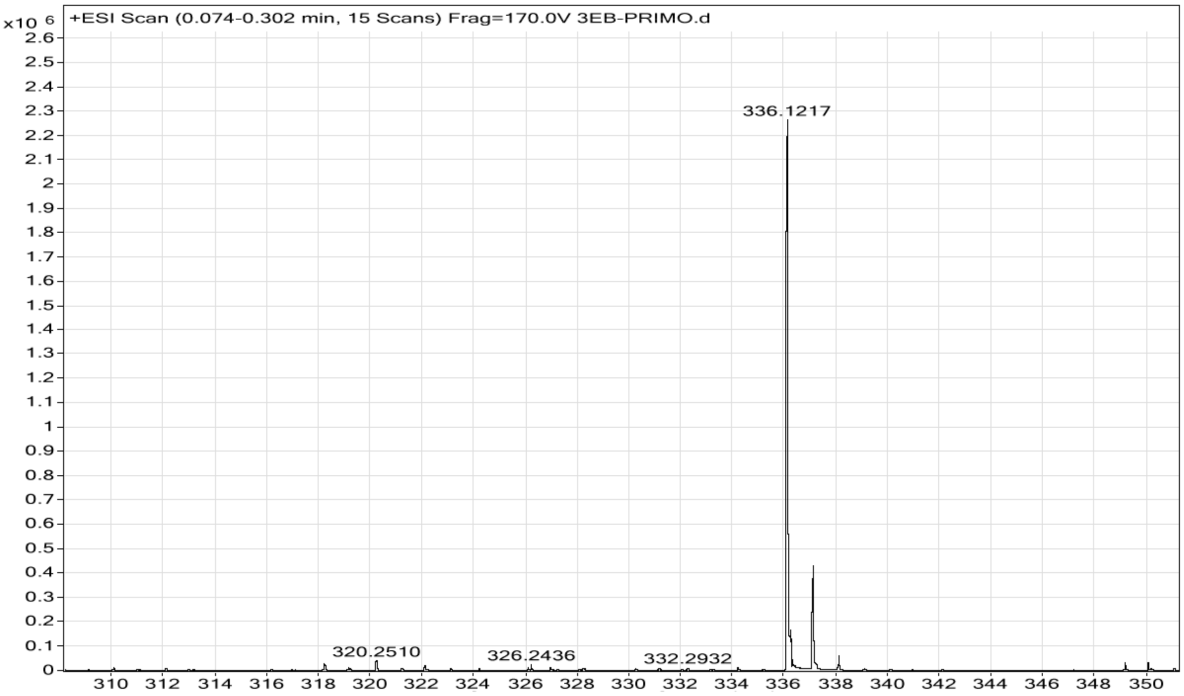

Methyl 5-(2-(diethylamino)-2-oxoethyl)-2-methylfuran-3-carboxylate (3ha)

Calcd. for $C_{13}H_{19}NNaO_4^+$ 276.1206; Found 276.1218.

**Sample Name**	See data file	**Position**		**Instrument Name**	Instrument 1	**User Name**	QTOF-HP\admin
**Inj Vol**	-1	**InjPosition**		**SampleType**	Sample	**IRM Calibration Status**	Success
**Data Filename**	3HA.d	**ACQ Method**	infusione-10uL-min.m	**Comment**		**Acquired Time**	12/6/2022 9:13:33 AM

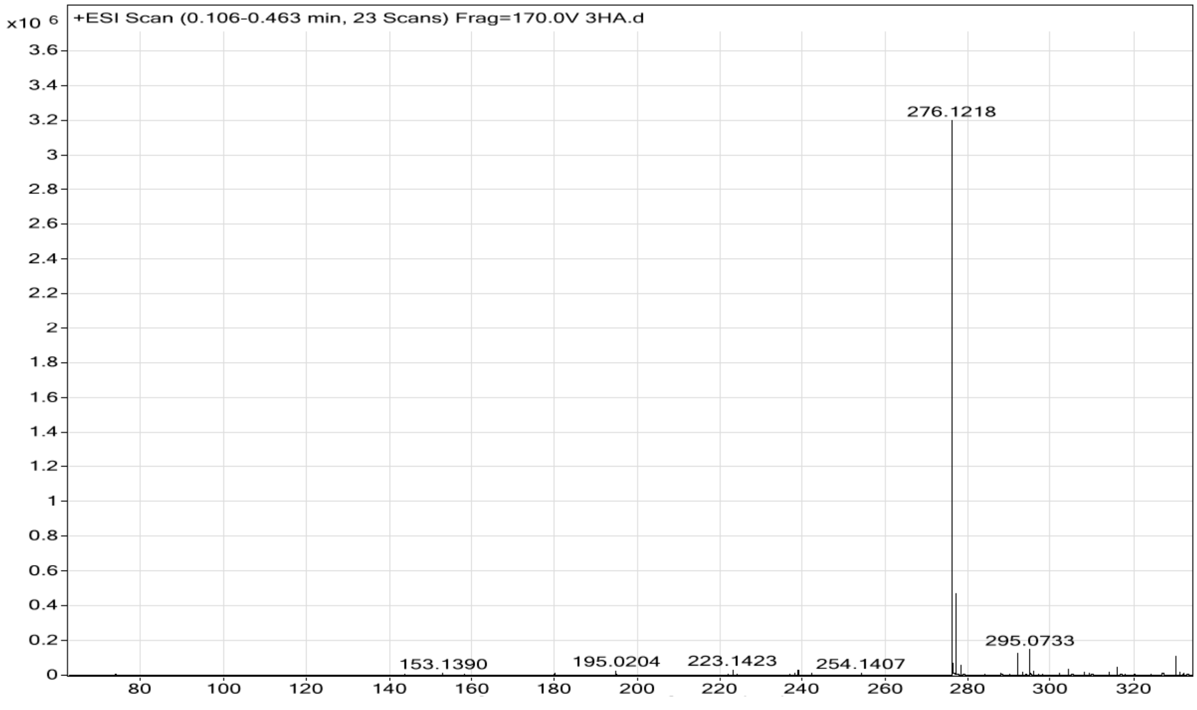

Ethyl 5-(2-(diethylamino)-2-oxoethyl)-2-methylfuran-3-carboxylate (3ia)

Calcd. for $C_{14}H_{21}NNaO_4^+$ 290.1363; Found 290.1368

**Sample Name**	See data file	**Position**		**Instrument Name**	Instrument 1	**User Name**	QTOF-HP\admin
**Inj Vol**	-1	**InjPosition**		**SampleType**	Sample	**IRM Calibration Status**	Success
**Data Filename**	4BA.d	**ACQ Method**	infusione-10uL-min.m	**Comment**		**Acquired Time**	2/28/2022 12:19:56 PM

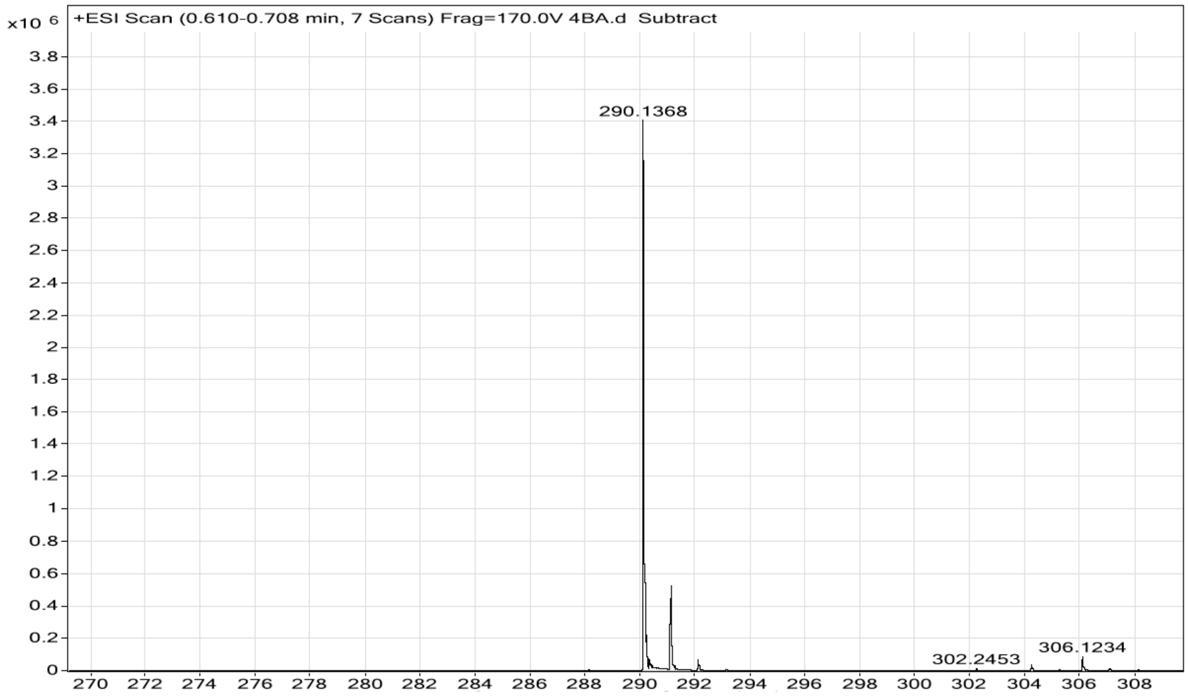

Ethyl 5-(2-(diethylamino)-2-oxoethyl)-2-propylfuran-3-carboxylate (3ja)

Calcd. for $C_{16}H_{25}NNaO_4^+$ 318.1676; Found 318.1683.

**Sample Name**	See data file	**Position**		**Instrument Name**	Instrument 1	**User Name**	QTOF-HP\admin
**Inj Vol**	-1	**InjPosition**		**SampleType**	Sample	**IRM Calibration Status**	Success
**Data Filename**	4CA.d	**ACQ Method**	infusione-10uL-min.m	**Comment**		**Acquired Time**	2/28/2022 12:42:42 PM

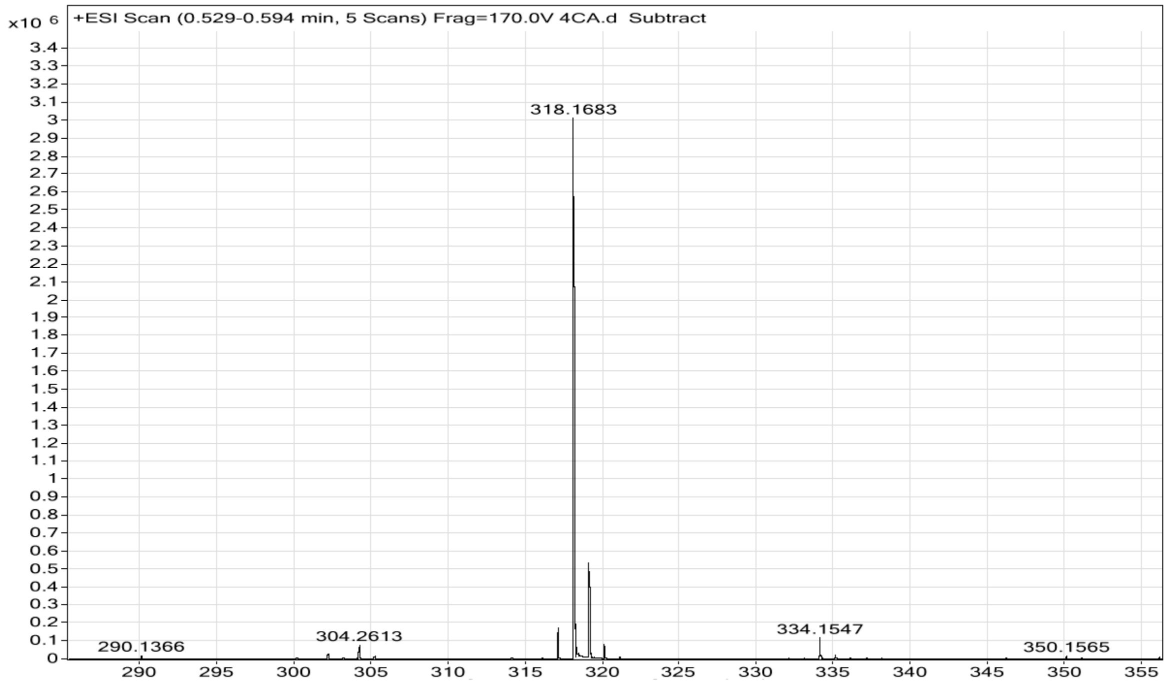

Ethyl 5-(2-(diethylamino)-2-oxoethyl)-2-isopropylfuran-3-carboxylate (3ka)

Calcd. for $C_{16}H_{25}NNaO_4^+$ 318.1676; Found 318.1675

**Sample Name**	See data file	**Position**		**Instrument Name**	Instrument 1	**User Name**	QTOF-HP\admin
**Inj Vol**	-1	**InjPosition**		**SampleType**	Sample	**IRM Calibration Status**	Success
**Data Filename**	4DA.d	**ACQ Method**	infusione-10uL-min.m	**Comment**		**Acquired Time**	2/28/2022 12:52:28 PM

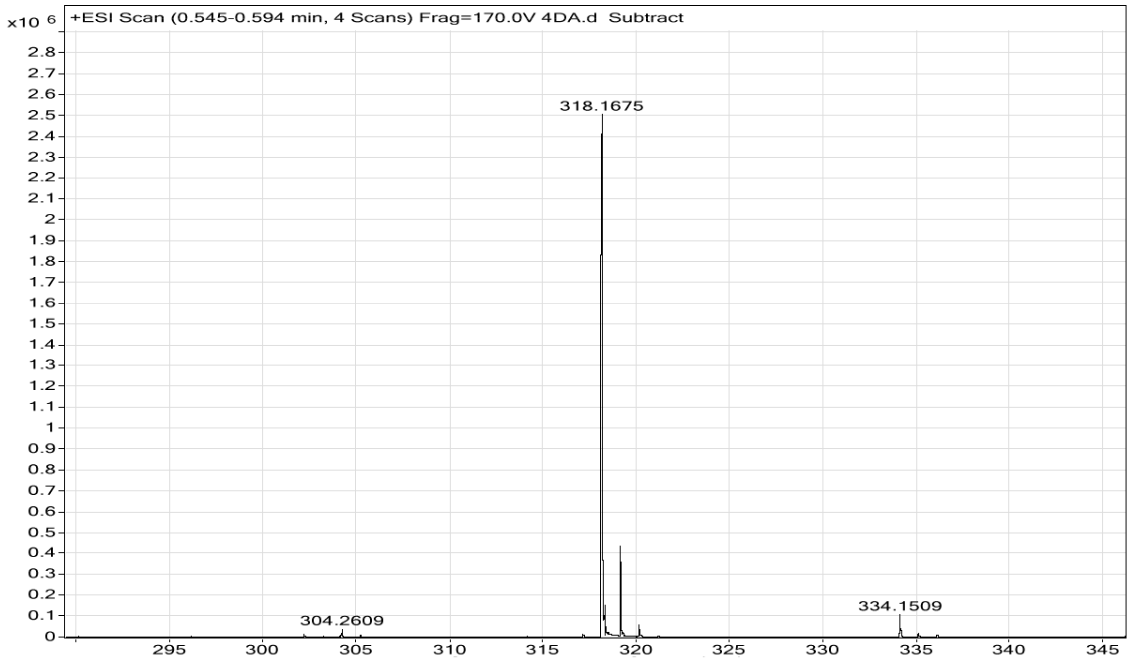

Benzyl 5-(2-(diethylamino)-2-oxoethyl)-2-methylfuran-3-carboxylate (3la).

Calcd. for $C_{19}H_{23}NNaO_4^+$ 352.1519; Found 352.1543.

**Sample Name**	See data file	**Position**		**Instrument Name**	Instrument 1	**User Name**	QTOF-HP\admin
**Inj Vol**	-1	**InjPosition**		**SampleType**	Sample	**IRM Calibration Status**	Success
**Data Filename**	3GA.d	**ACQ Method**	infusione-10uL-min.m	**Comment**		**Acquired Time**	12/6/2022 9:00:07 AM

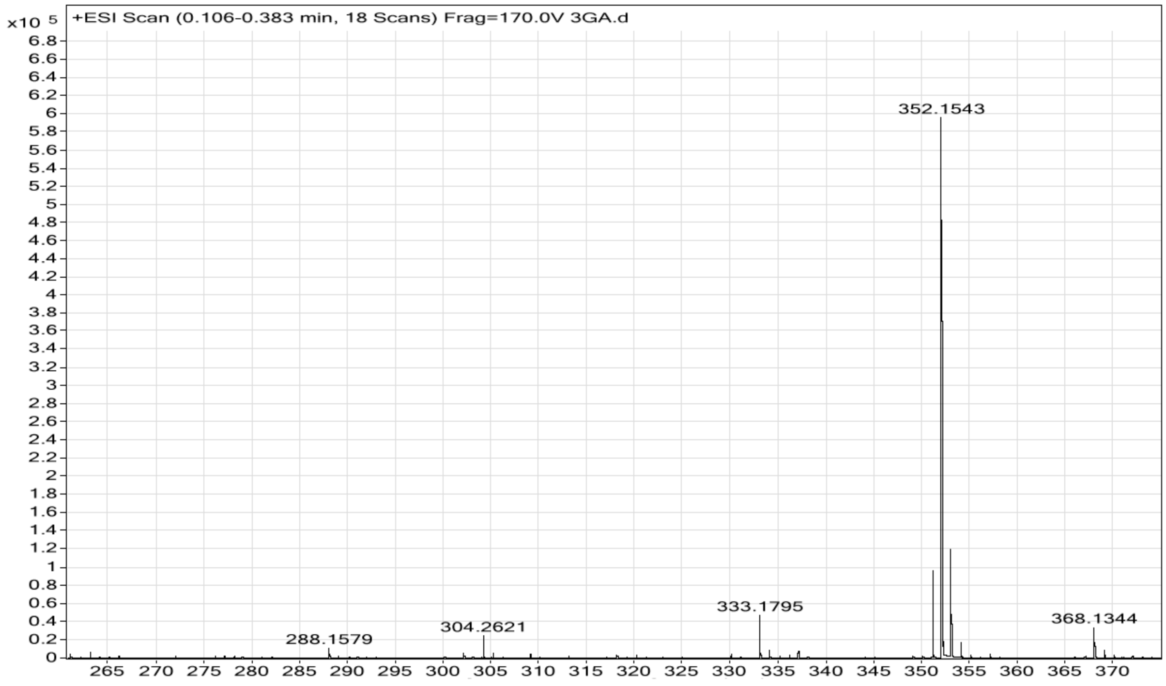

5-(2-(Diethylamino)-2-oxoethyl)-*N*,*N*-diethyl-2-methylfuran-3-carboxamide (3ma)

Calcd. for $C_{16}H_{26}N_2NaO_3^+$ 317.1836; Found 317.1837

**Sample Name**	See data file	**Position**		**Instrument Name**	Instrument 1	**User Name**	QTOF-HP\admin
**Inj Vol**	-1	**InjPosition**		**SampleType**	Sample	**IRM Calibration Status**	Success
**Data Filename**	4GA.d	**ACQ Method**	infusione-10uL-min.m	**Comment**		**Acquired Time**	2/28/2022 12:32:07 PM

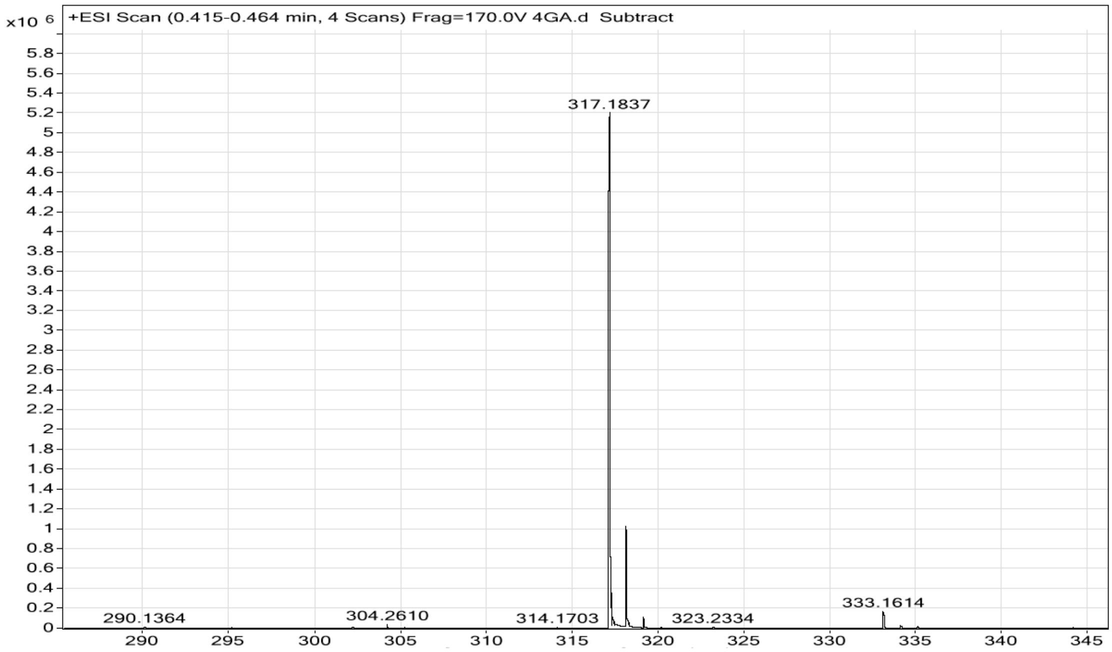

***N,N*-Dibutyl-2-(5-phenyl-4-tosylfuran-2-yl)acetamide (3nc)**

Calcd. for $C_{27}H_{33}NNaO_4S^+$ 490.2023 ; Found 490.2037

**Sample Name**	See data file	**Position**		**Instrument Name**	Instrument 1	**User Name**	QTOF-HP\admin
**Inj Vol**	-1	**InjPosition**		**SampleType**	Sample	**IRM Calibration Status**	Success
**Data Filename**	3NA.d	**ACQ Method**	infusione-10uL-min.m	**Comment**		**Acquired Time**	3/31/2023 9:45:26 AM

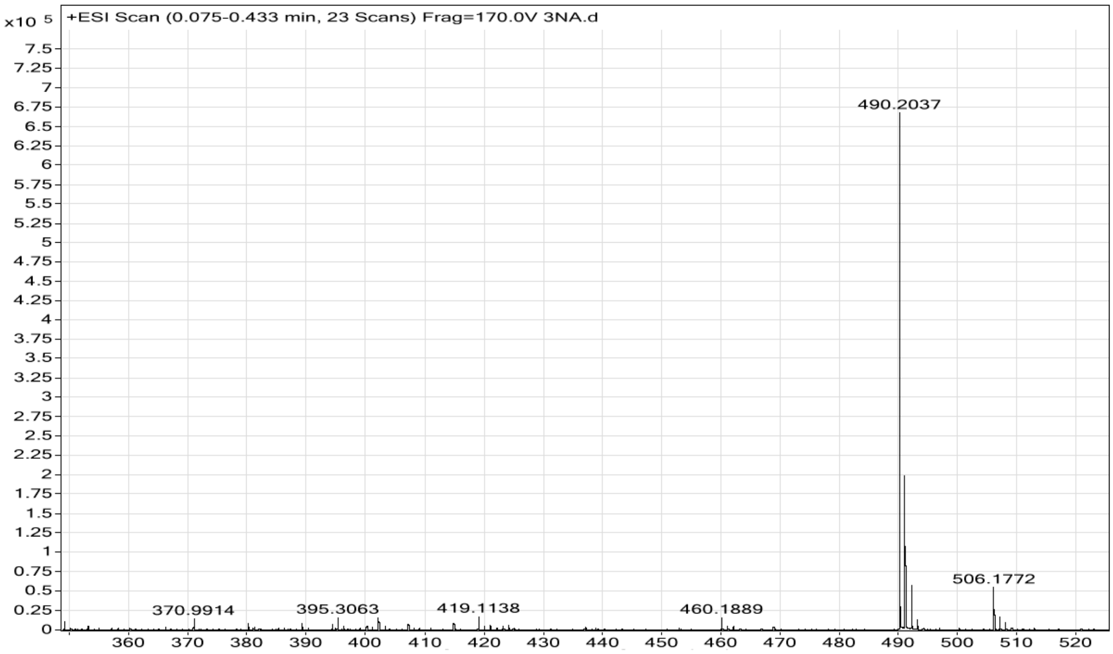

Copies of ^{1}H and ^{13}C NMR Spectra

3-(Prop-2-yn-1-yl)pentane-2,4-dione (1b)

^{1}H NMR (500 MHz CDCl$_3$)

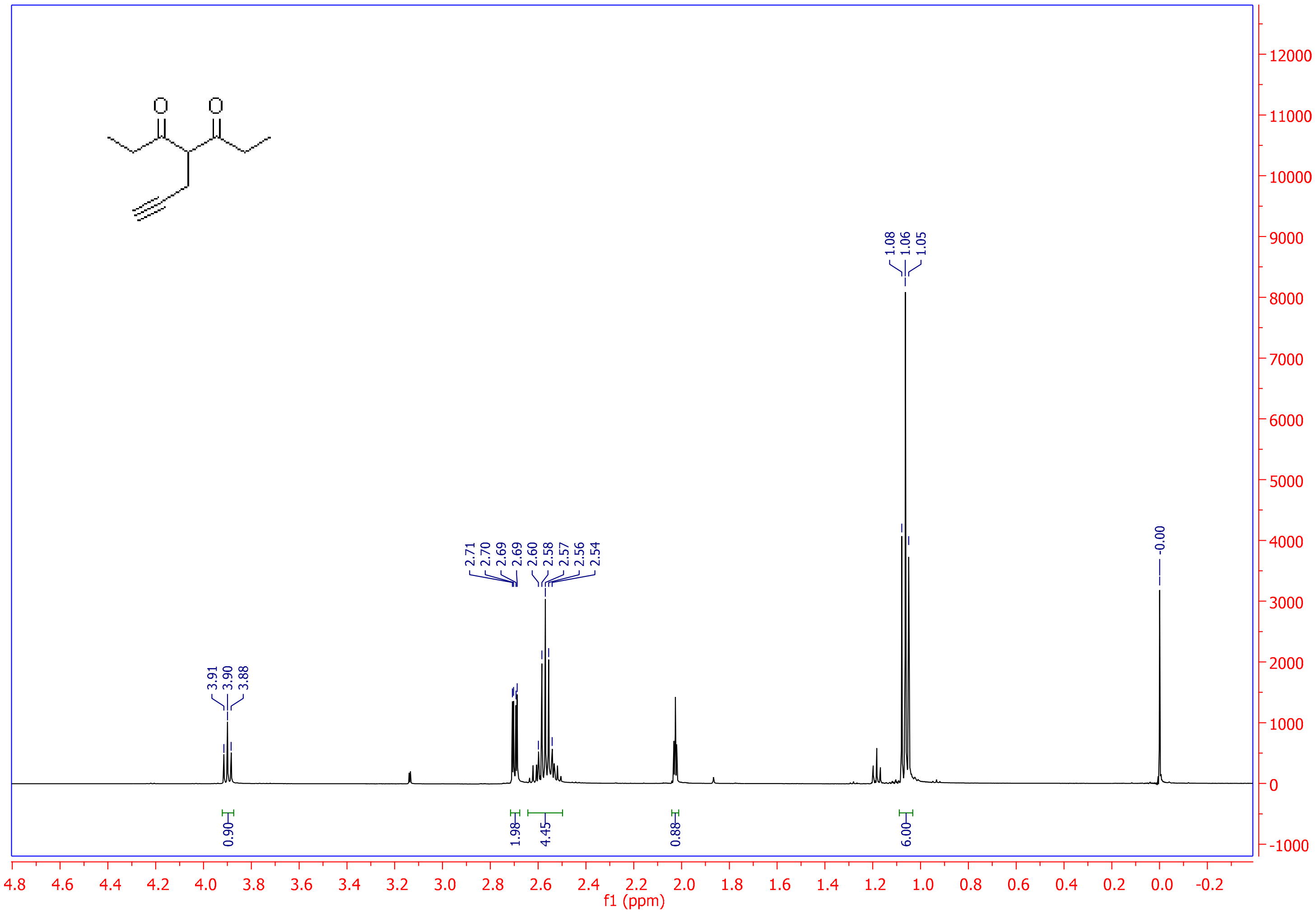

3-(Prop-2-yn-1-yl)pentane-2,4-dione (1b)

^{13}C NMR (125 MHz $CDCl_3$)

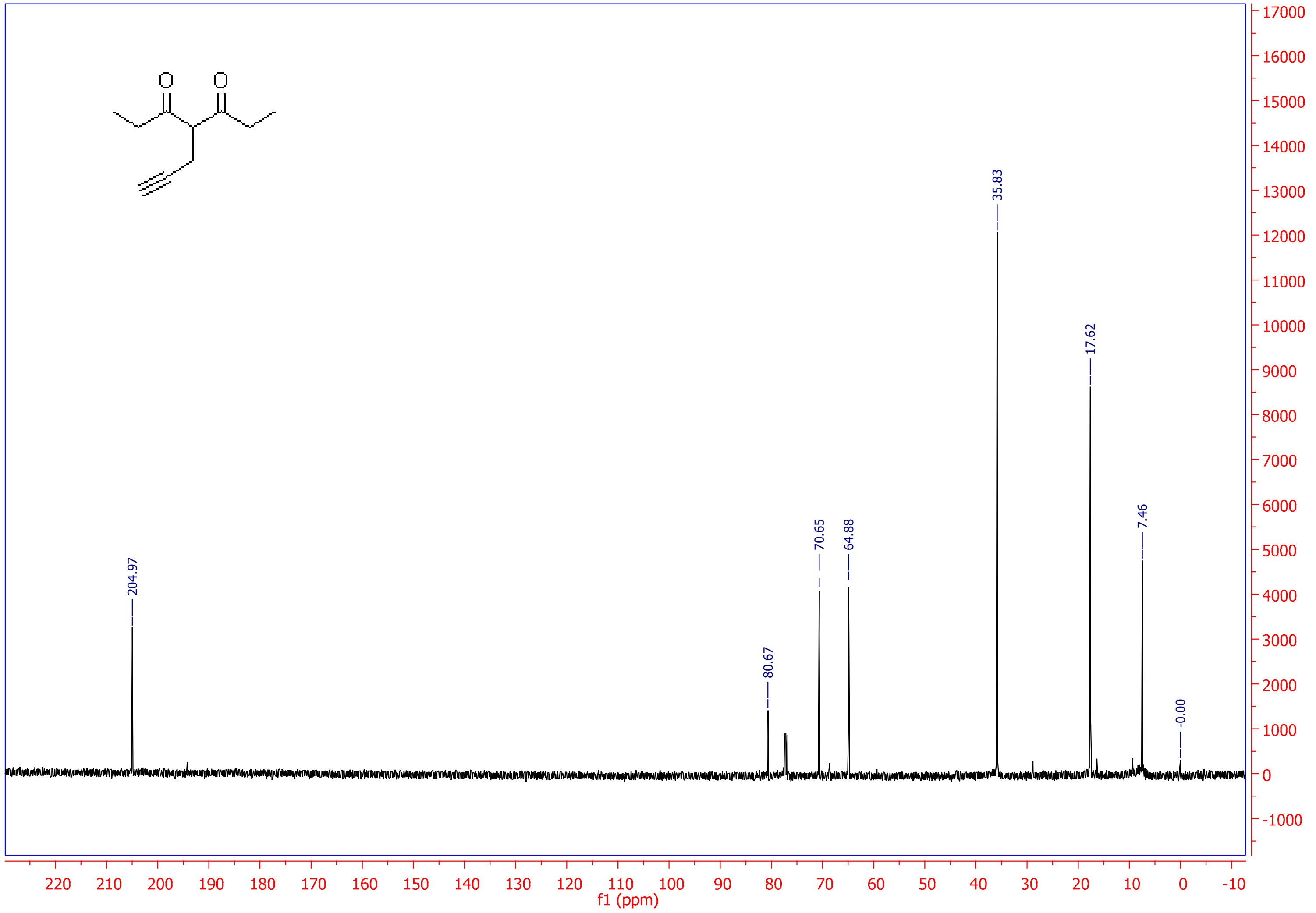

3-(But-3-yn-2-yl)pentane-2,4-dione (1d)

^{1}H NMR (300 MHz $CDCl_3$)

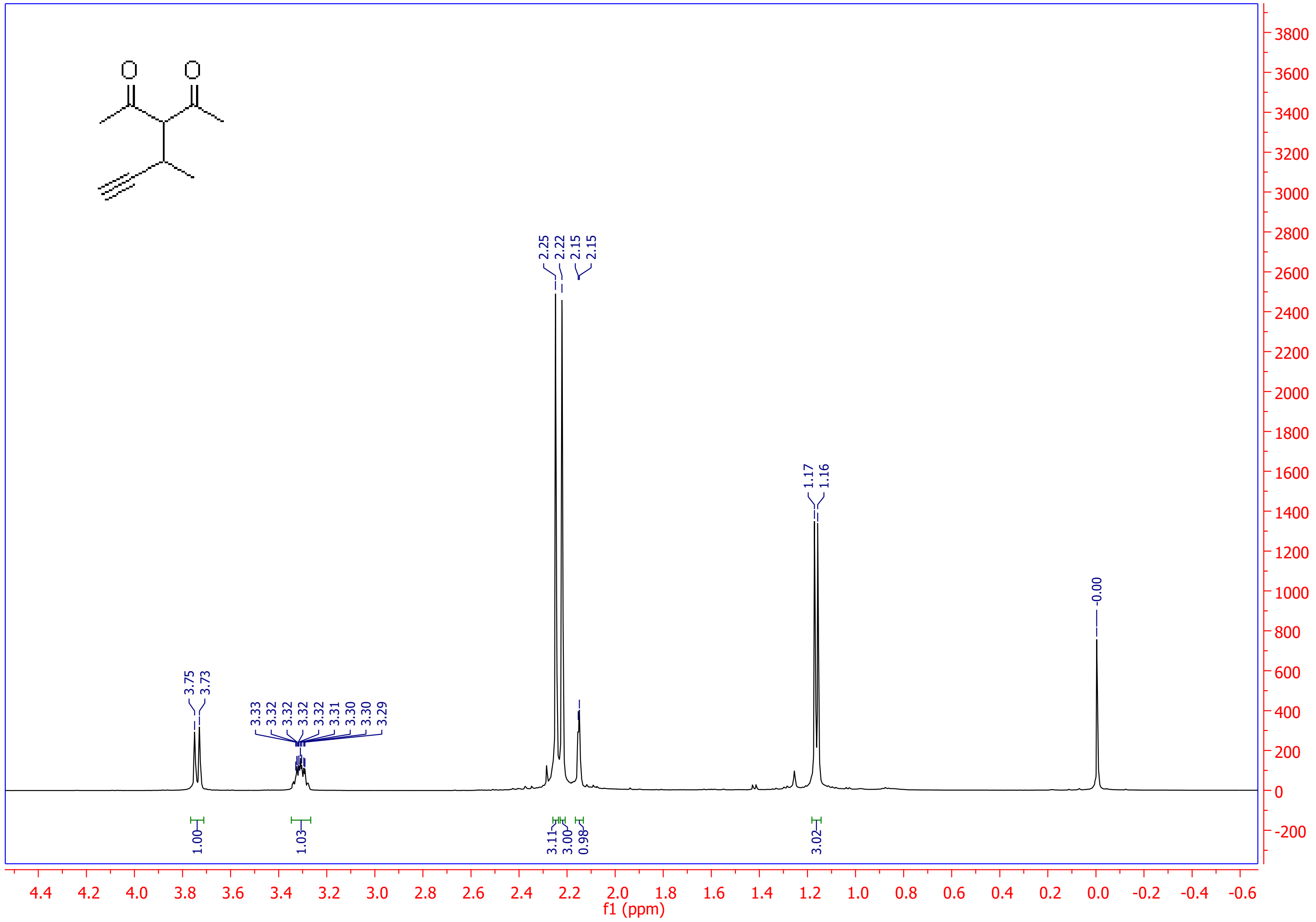

3-(But-3-yn-2-yl)pentane-2,4-dione (1d)

^{13}C NMR (125 MHz $CDCl_3$)

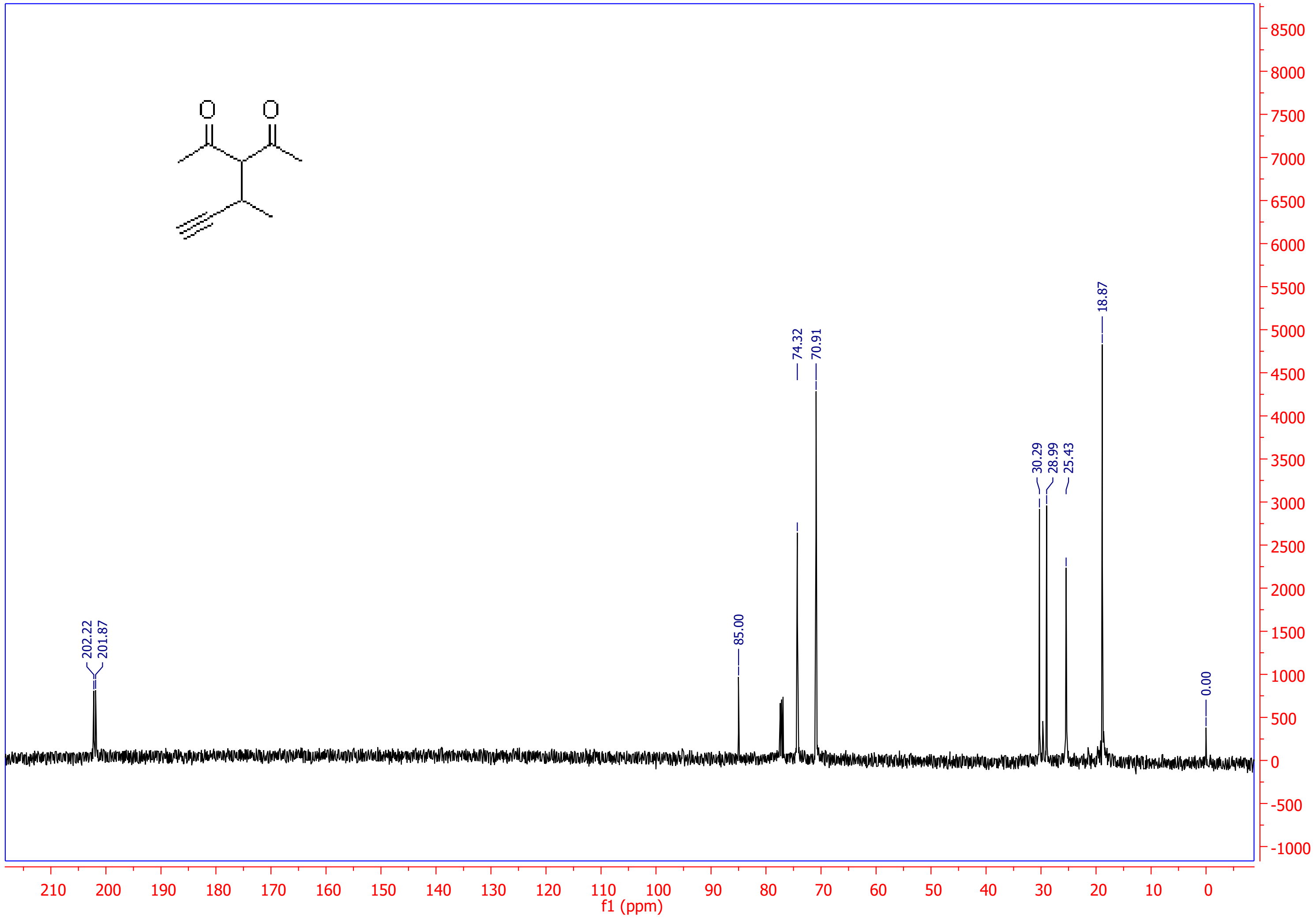

Ethyl 2-isobutyrylpent-4-ynoate (1k)

^{1}H NMR (500 MHz $CDCl_3$)

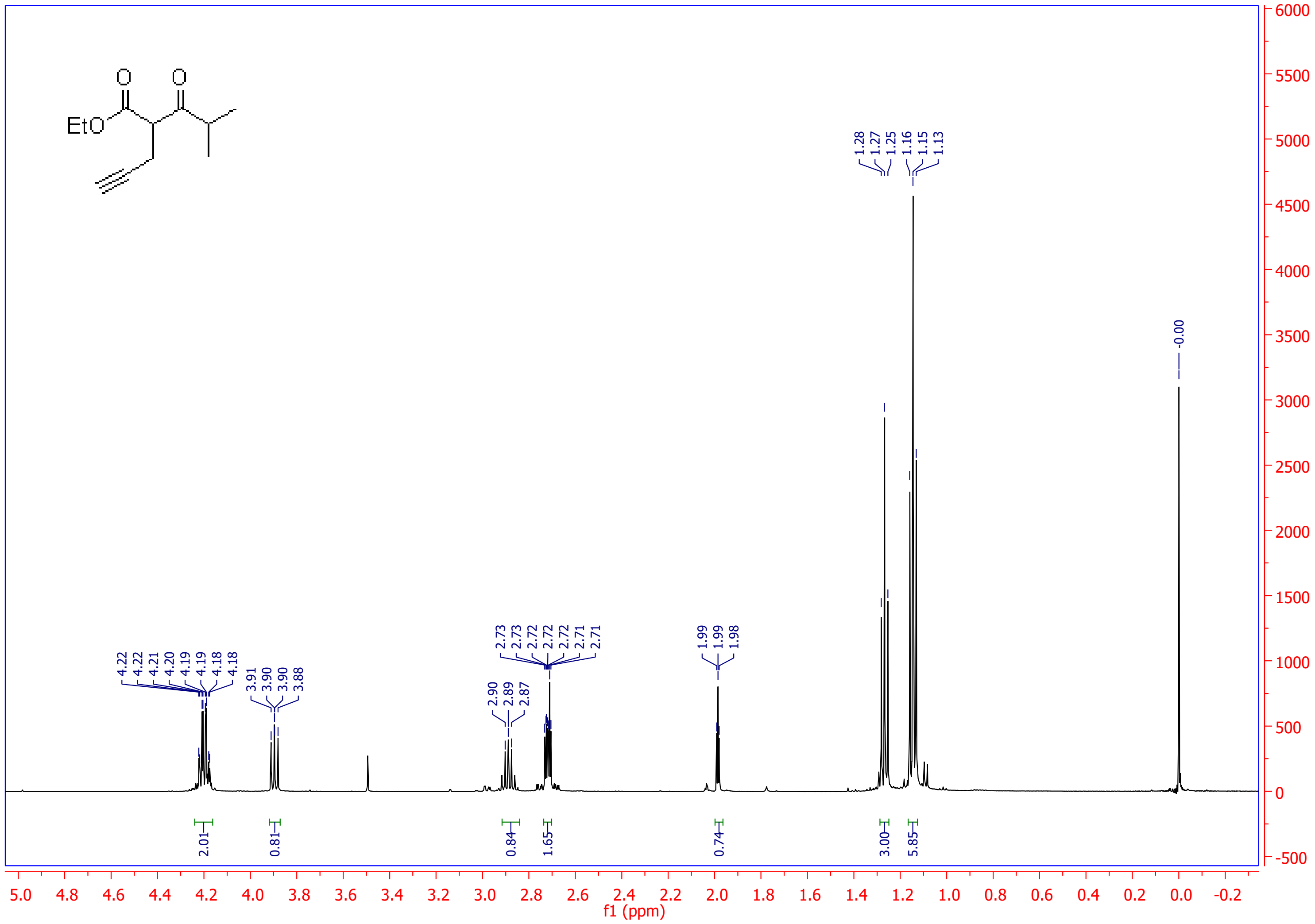

Ethyl 2-isobutyrylpent-4-ynoate (1k)

^{13}C NMR (125 MHz $CDCl_3$)

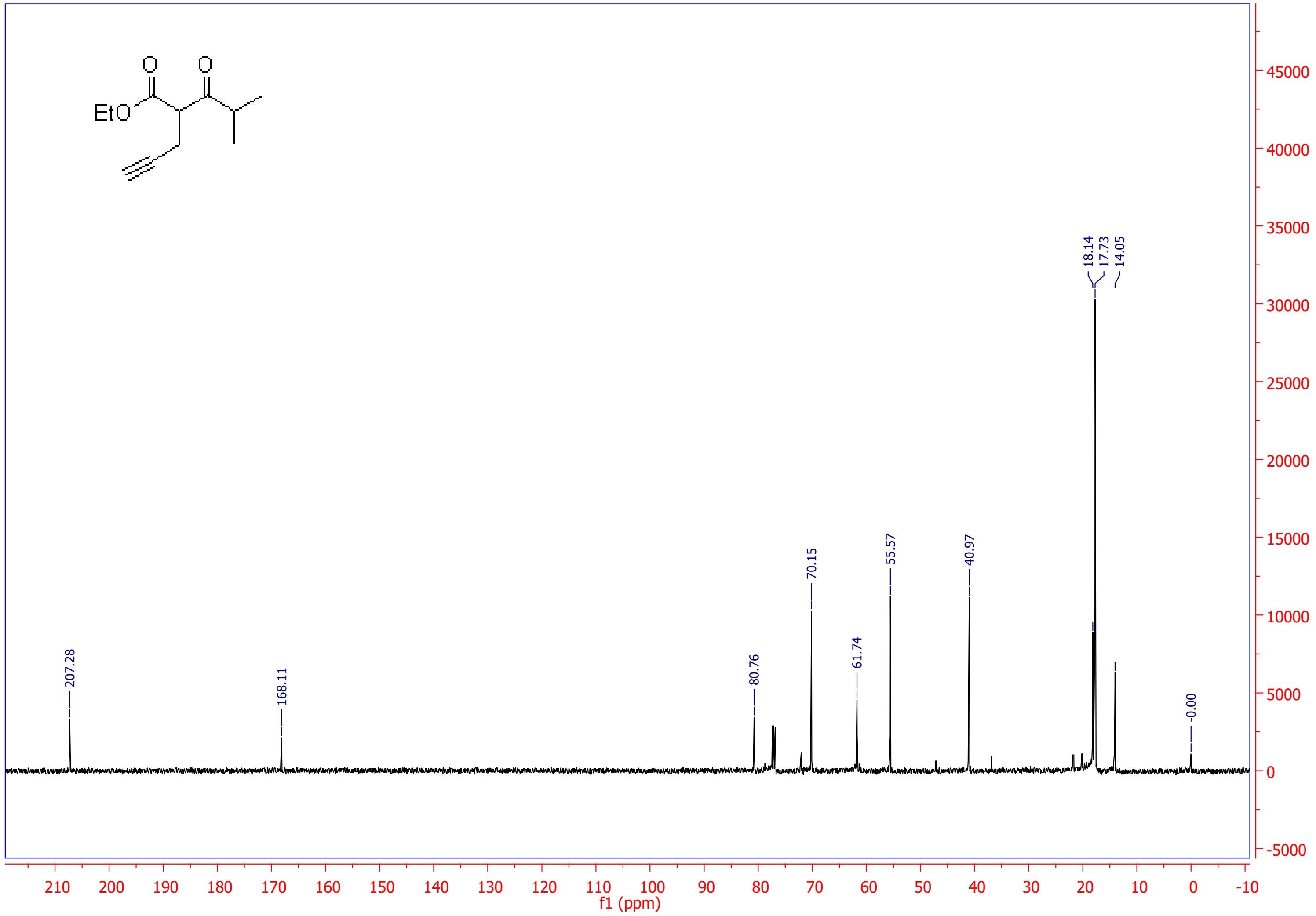

2-Acetyl-*N*,*N*-diethylpent-4-ynamide (1m)

^{1}H NMR (500 MHz $CDCl_3$)

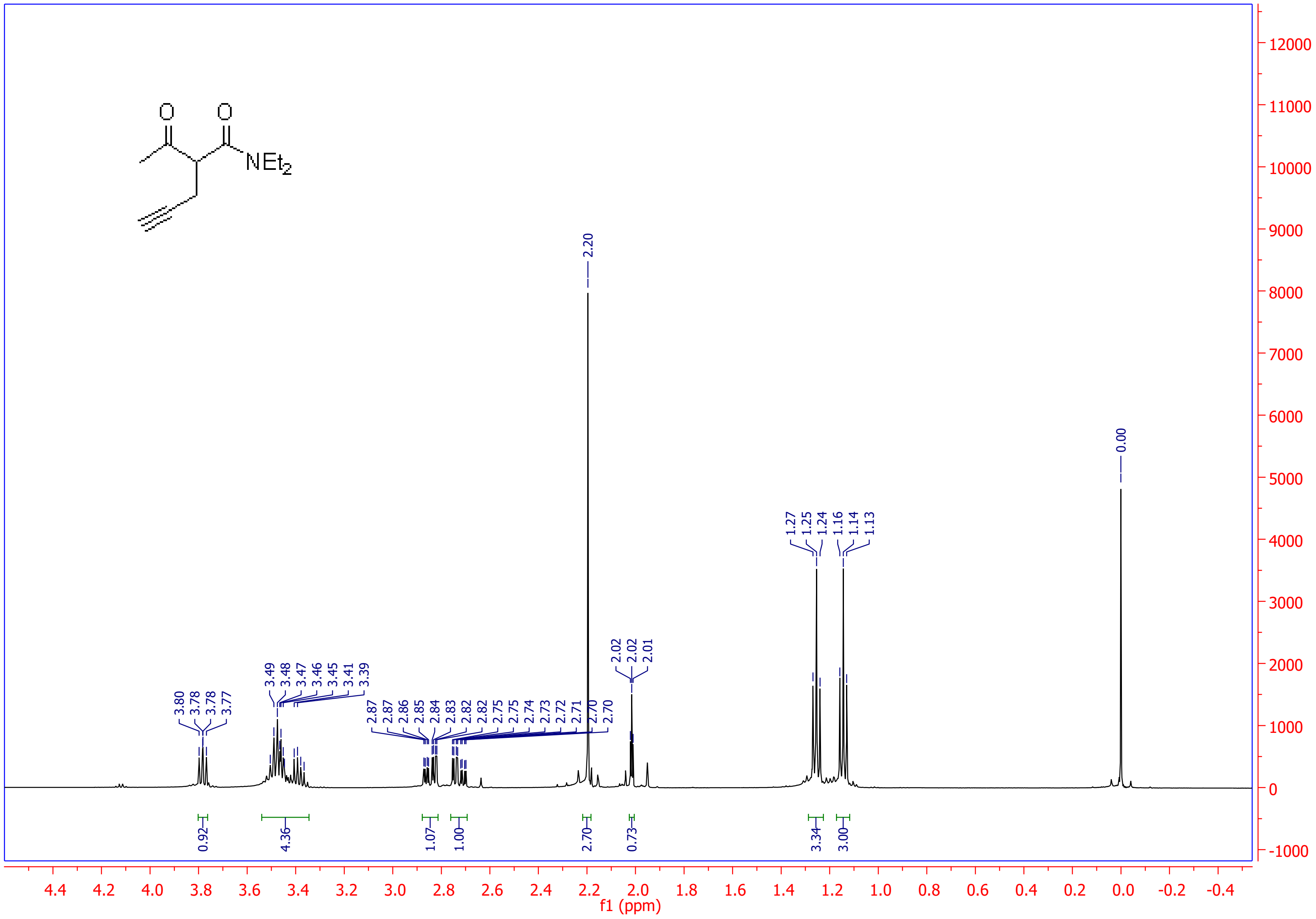

2-Acetyl-*N*,*N*-diethylpent-4-ynamide (1m)

^{13}C NMR (125 MHz $CDCl_3$)

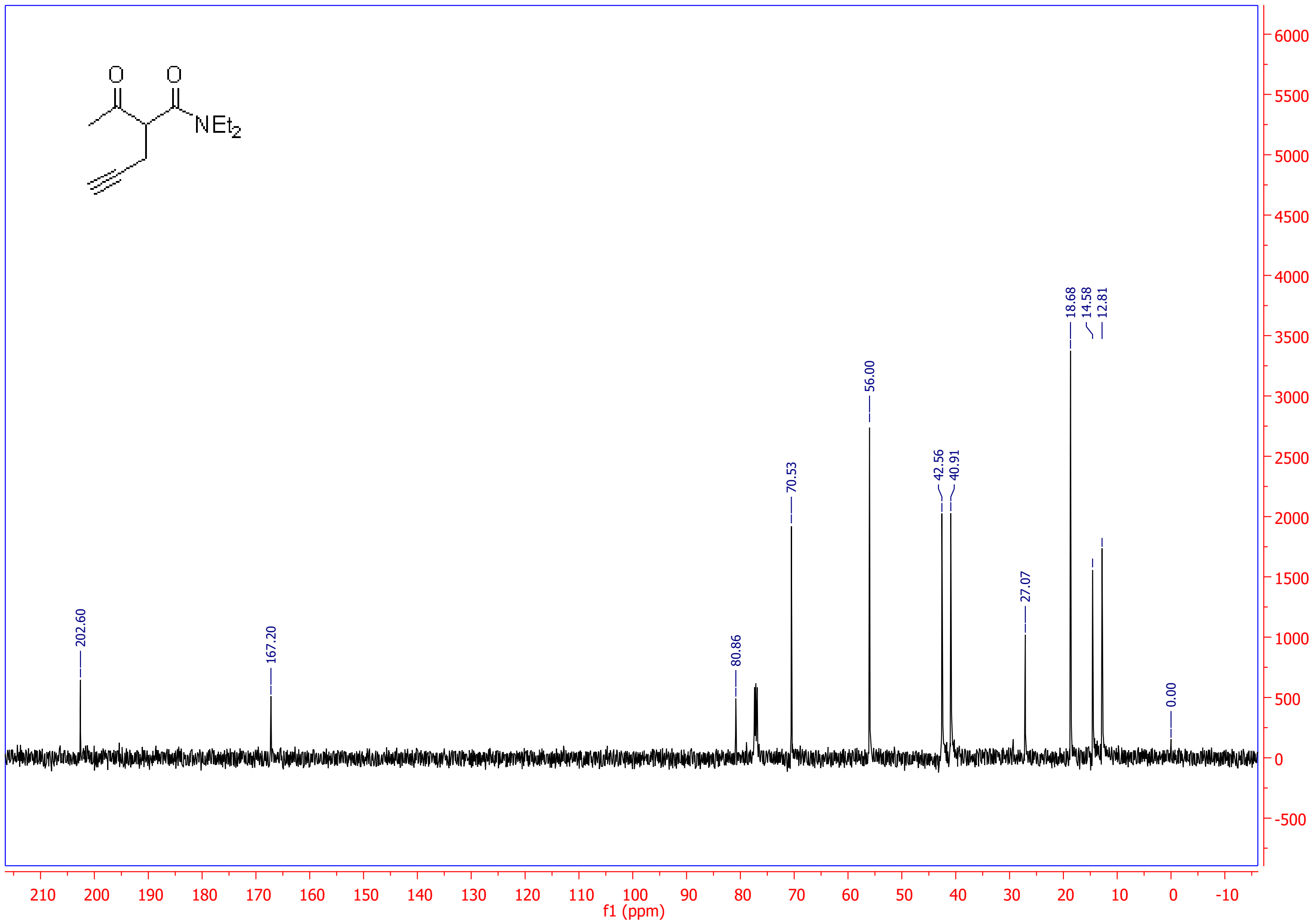

2-(4-Acetyl-5-methylfuran-2-yl)-*N,N*-diethylacetamide (3aa)

^{1}H NMR (500 MHz $CDCl_3$)

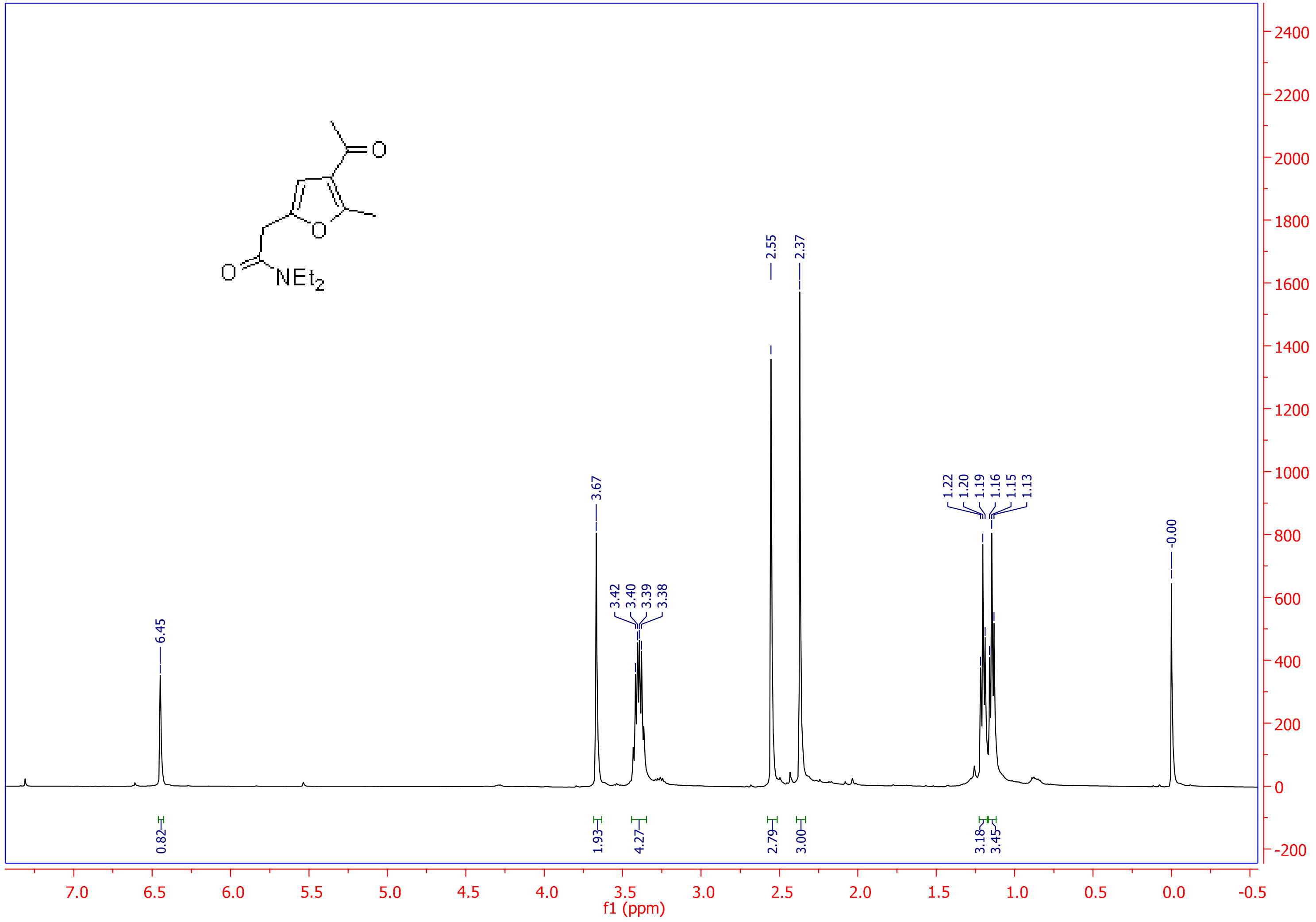

2-(4-Acetyl-5-methylfuran-2-yl)-*N,N*-diethylacetamide (3aa)

^{13}C NMR (125 MHz DMSO-d_6)

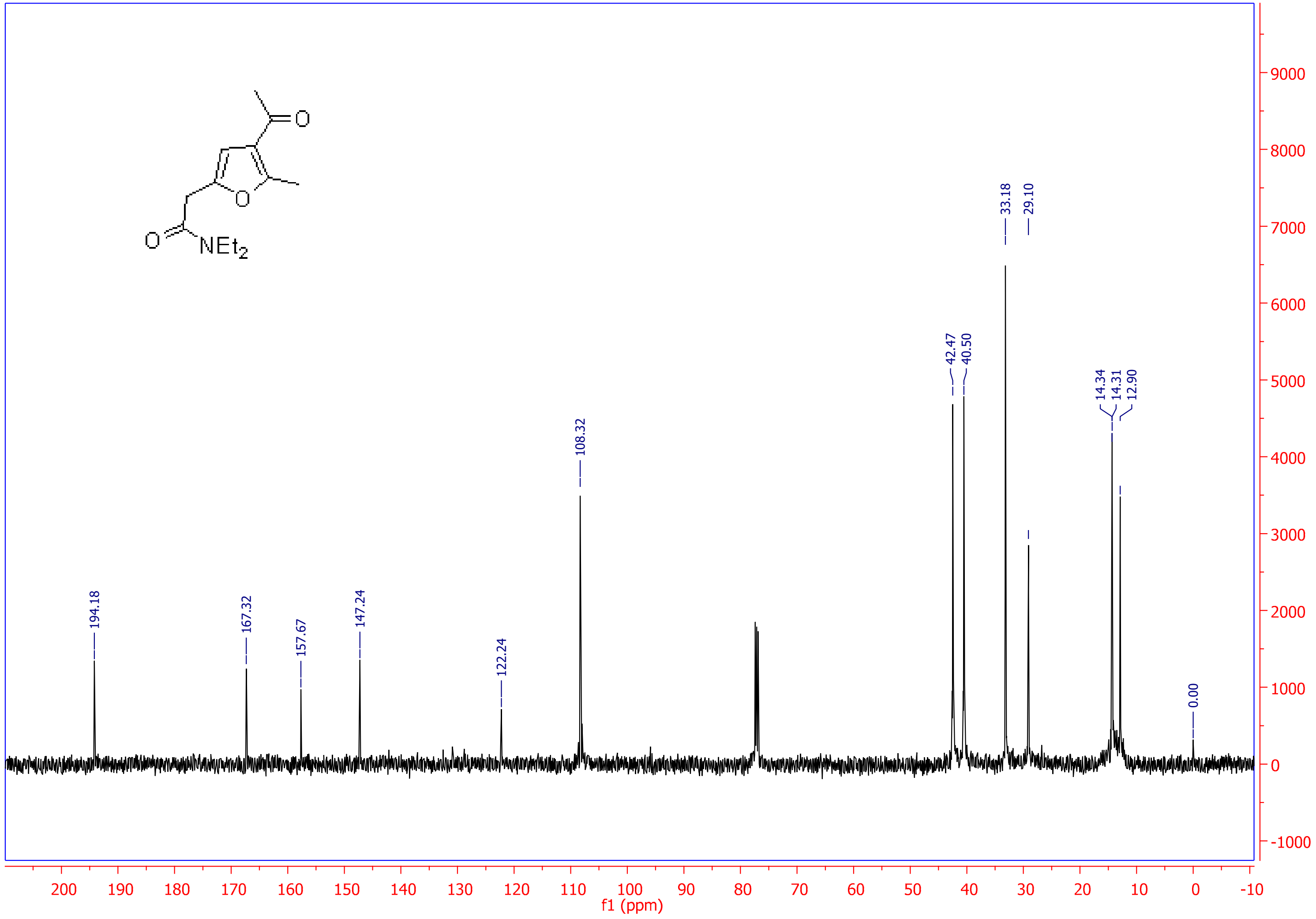

2-(4-Acetyl-5-methylfuran-2-yl)-*N,N*-dimethylacetamide (3ab)

^{1}H NMR (500 MHz $CDCl_3$)

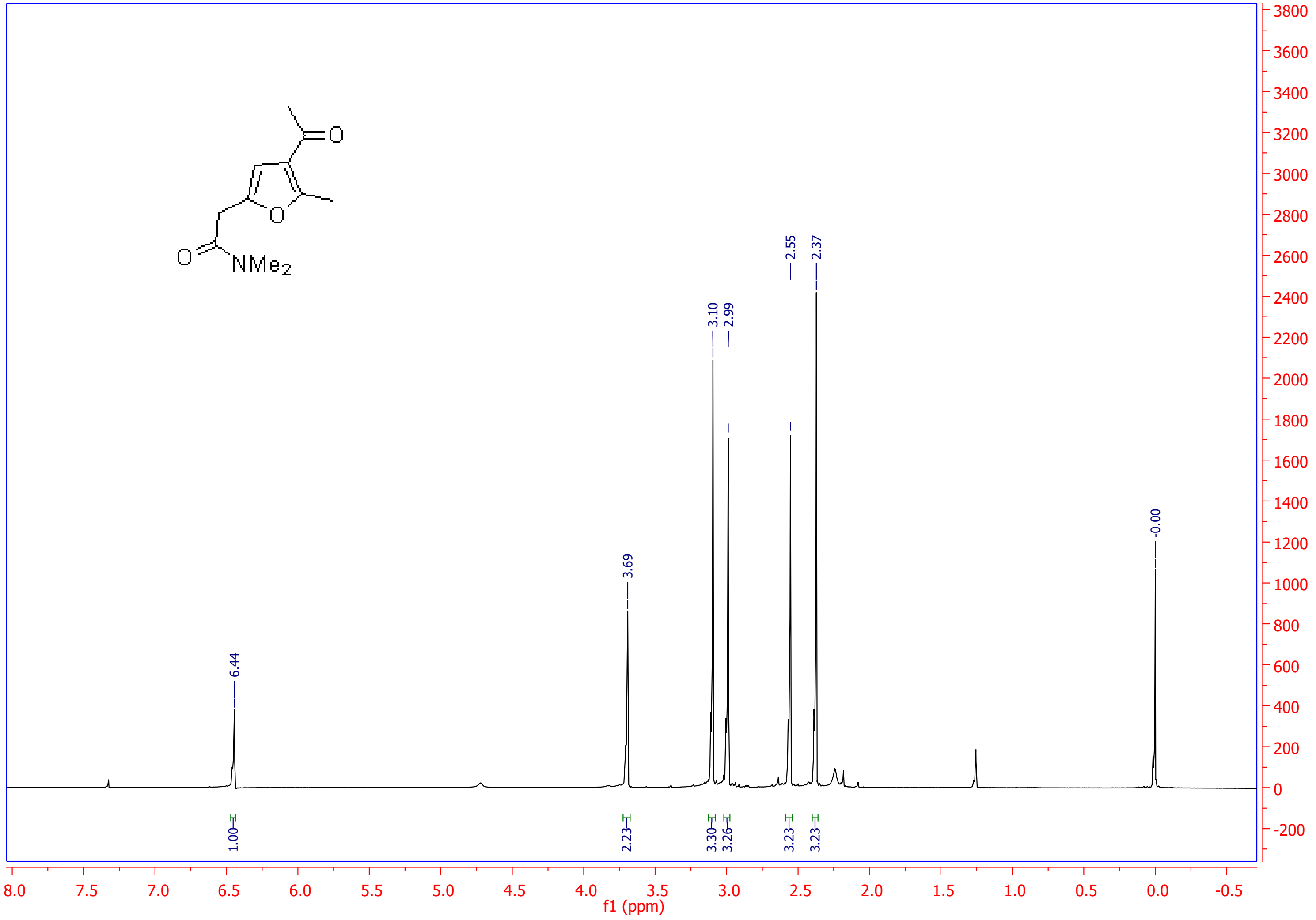

2-(4-Acetyl-5-methylfuran-2-yl)-*N,N*-dimethylacetamide (3ab)

^{13}C NMR (125 MHz $CDCl_3$)

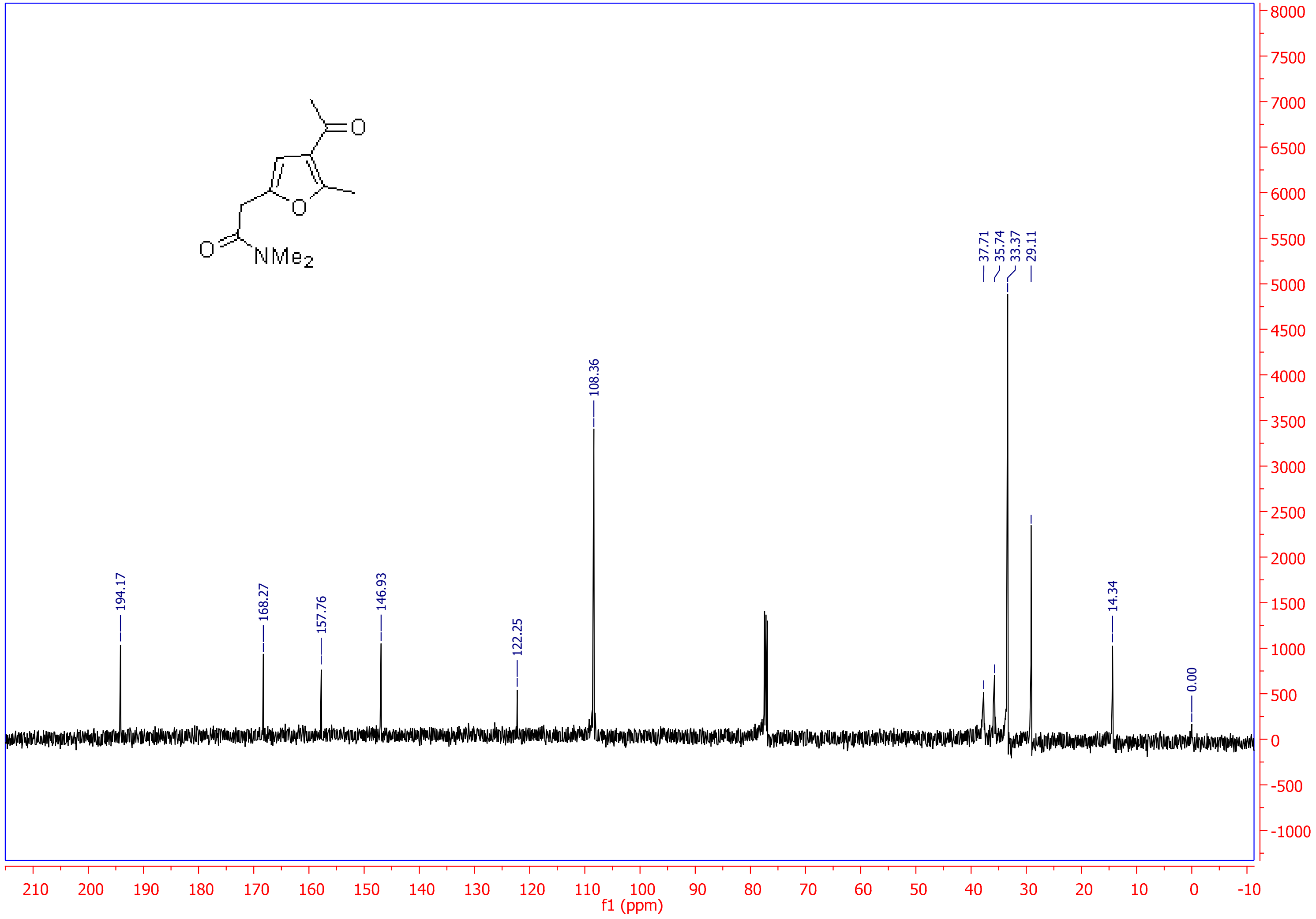

2-(4-Acetyl-5-methylfuran-2-yl)-*N,N*-dibutylacetamide (3ac)

^{1}H NMR (500 MHz $CDCl_3$)

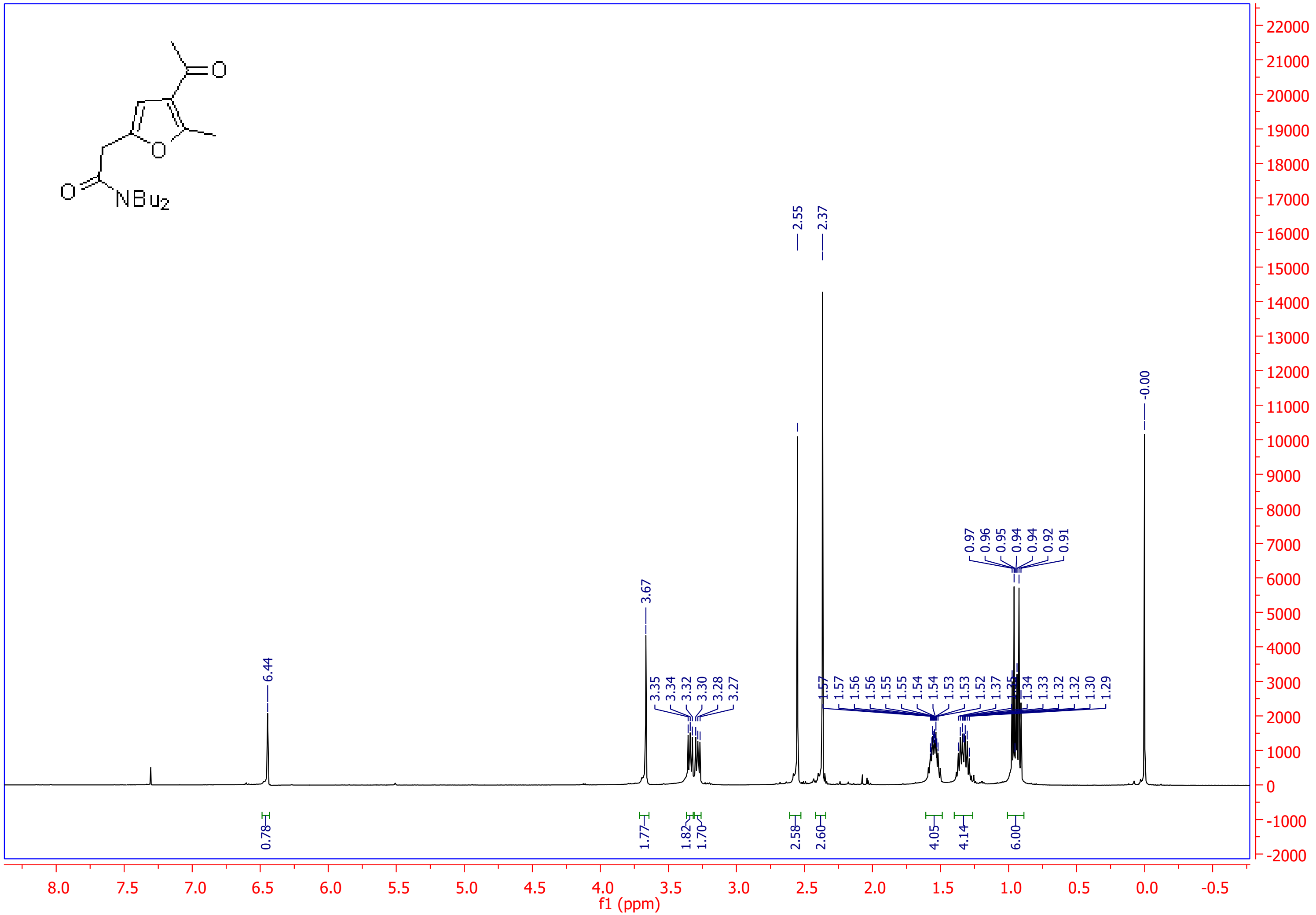

2-(4-Acetyl-5-methylfuran-2-yl)-*N,N*-dibutylacetamide (3ac)

^{13}C NMR (125 MHz $CDCl_3$)

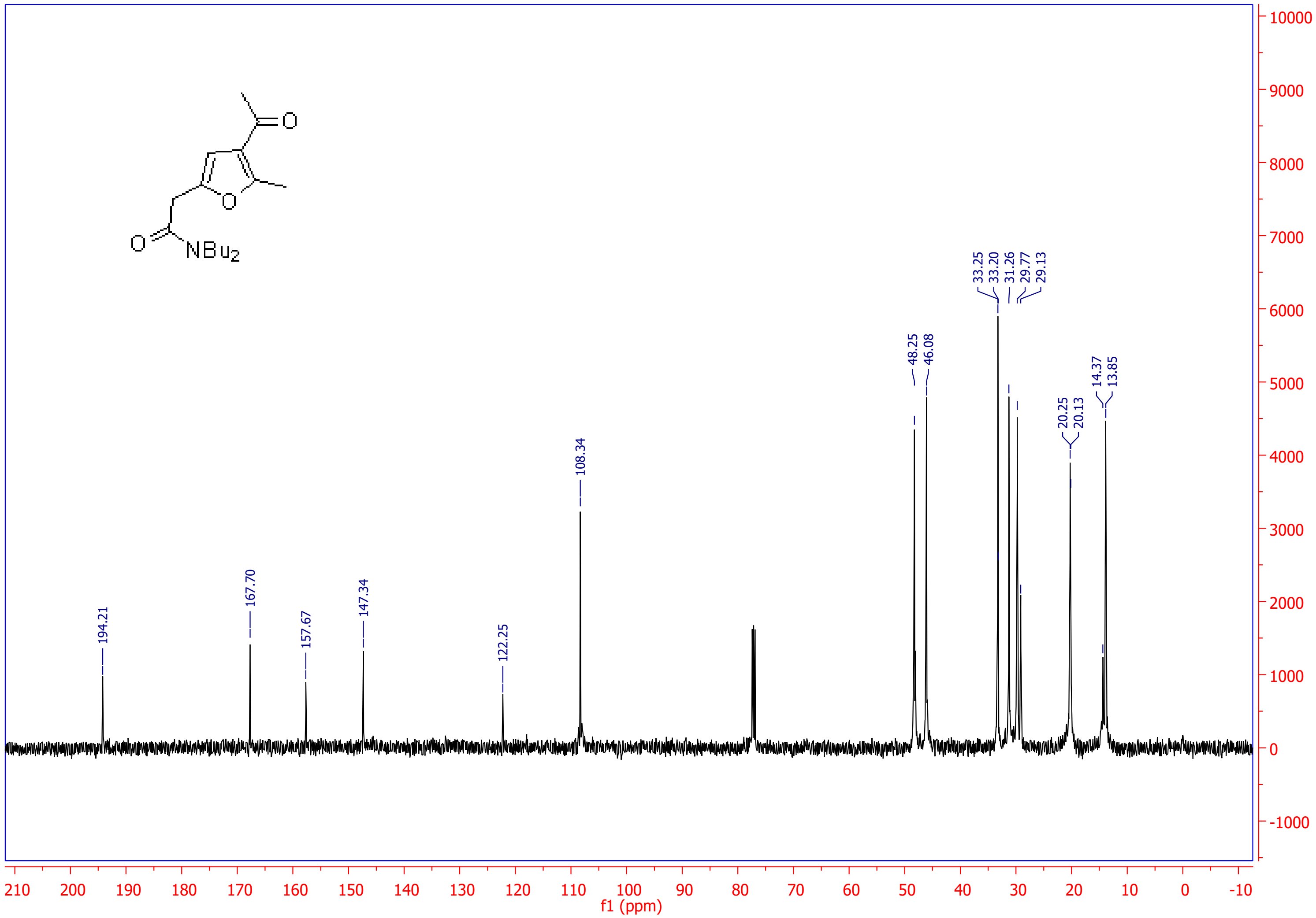

2-(4-Acetyl-5-methylfuran-2-yl)-*N*-cyclohexyl-*N*-ethylacetamide (3ad)

(Mixture of rotamers A + B, A/B ca 1.2 by ^{1}H NMR)

^{1}H NMR (500 MHz $CDCl_3$)

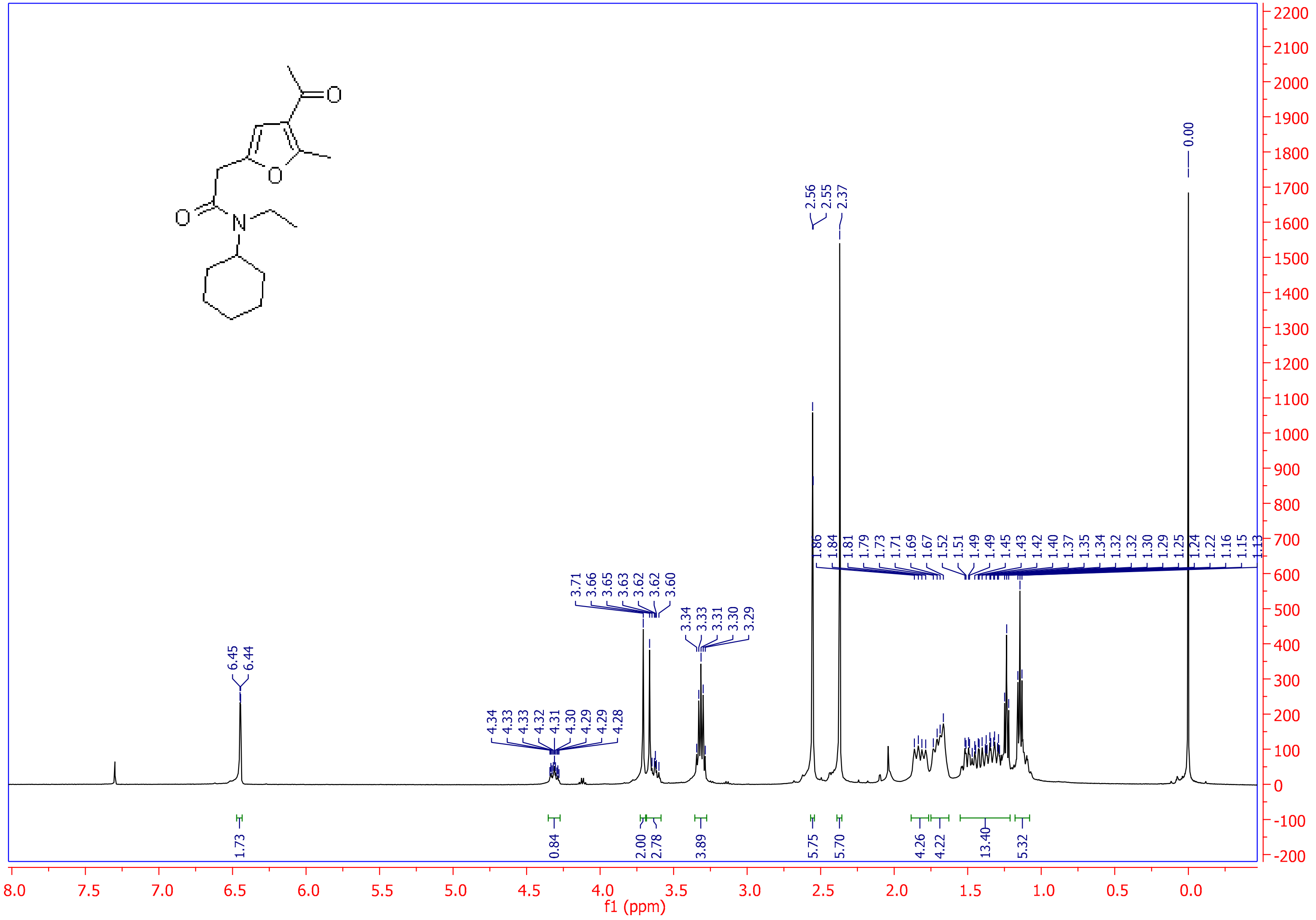

2-(4-Acetyl-5-methylfuran-2-yl)-*N*-cyclohexyl-*N*-ethylacetamide (3ad)

(Mixture of rotamers A + B, A/B ca 1.2 by ^{1}H NMR)

^{13}C NMR (125 MHz $CDCl_3$)

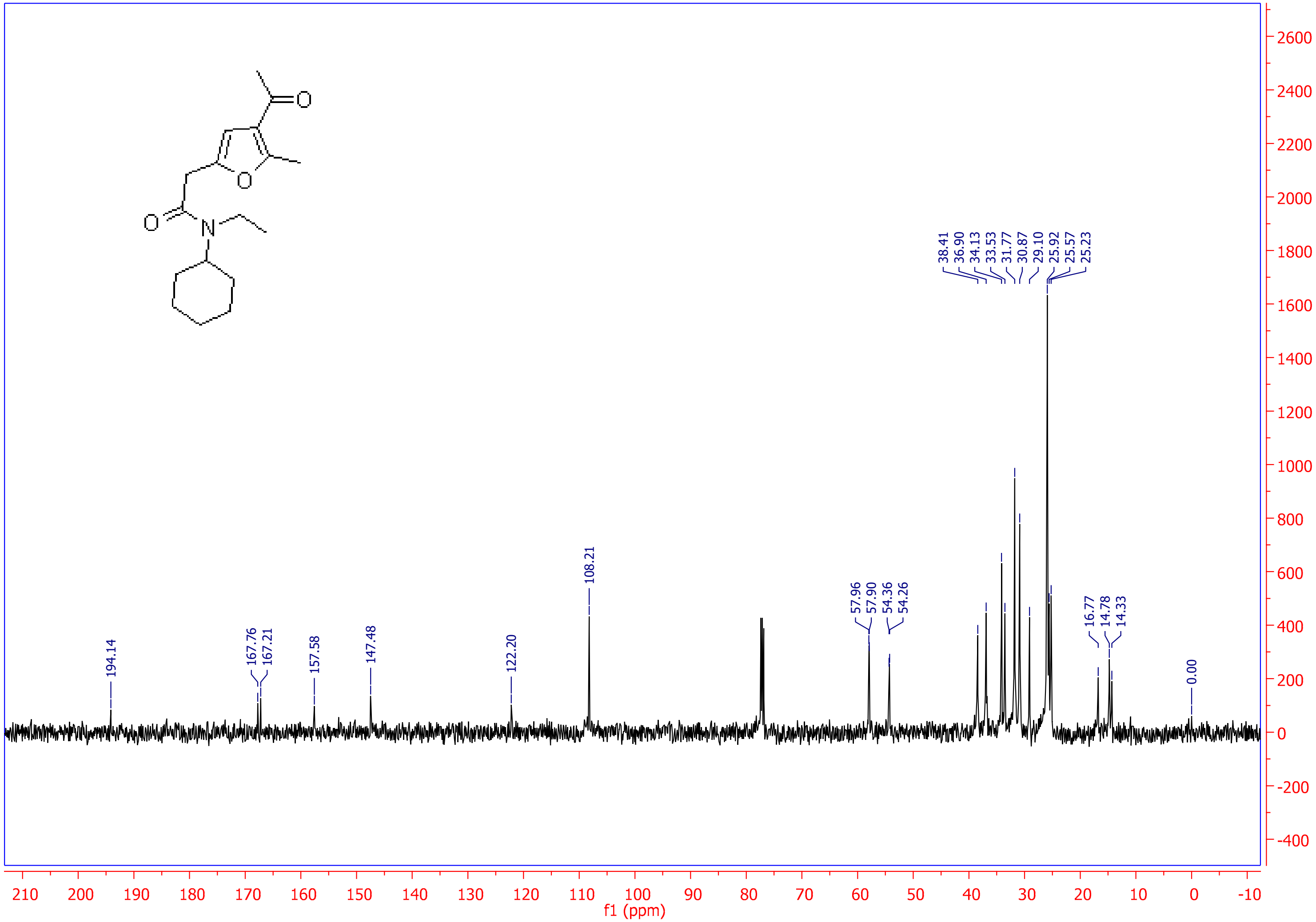

2-(4-Acetyl-5-methylfuran-2-yl)-*N,N*-diisopropylacetamide (3ae)

^{1}H NMR (500 MHz $CDCl_3$)

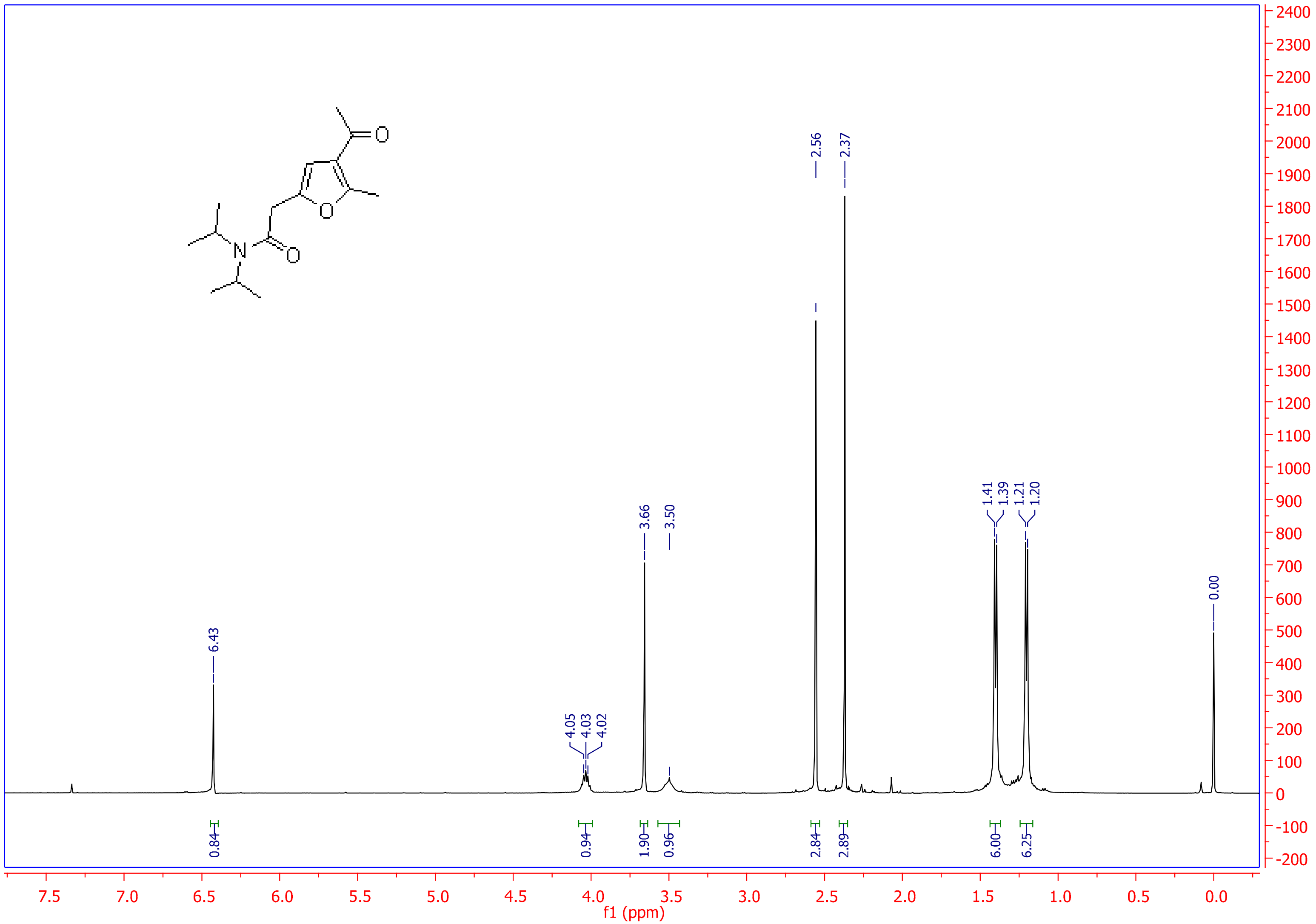

2-(4-Acetyl-5-methylfuran-2-yl)-*N,N*-diisopropylacetamide (3ae).

^{13}C NMR (125 MHz $CDCl_3$)

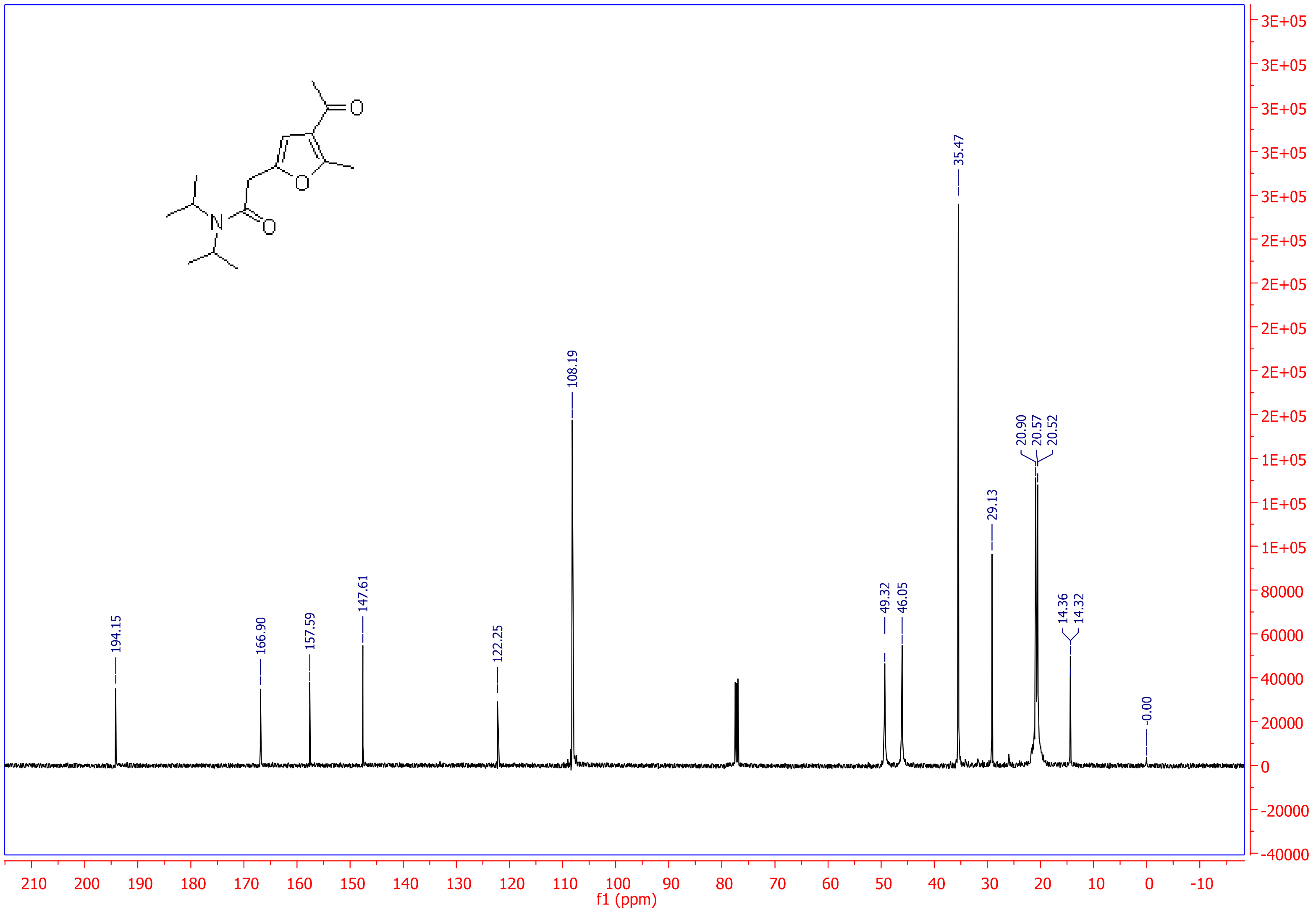

2-(4-Acetyl-5-methylfuran-2-yl)-1-morpholinoethan-1-one (3af)

^{1}H NMR (500 MHz $CDCl_3$)

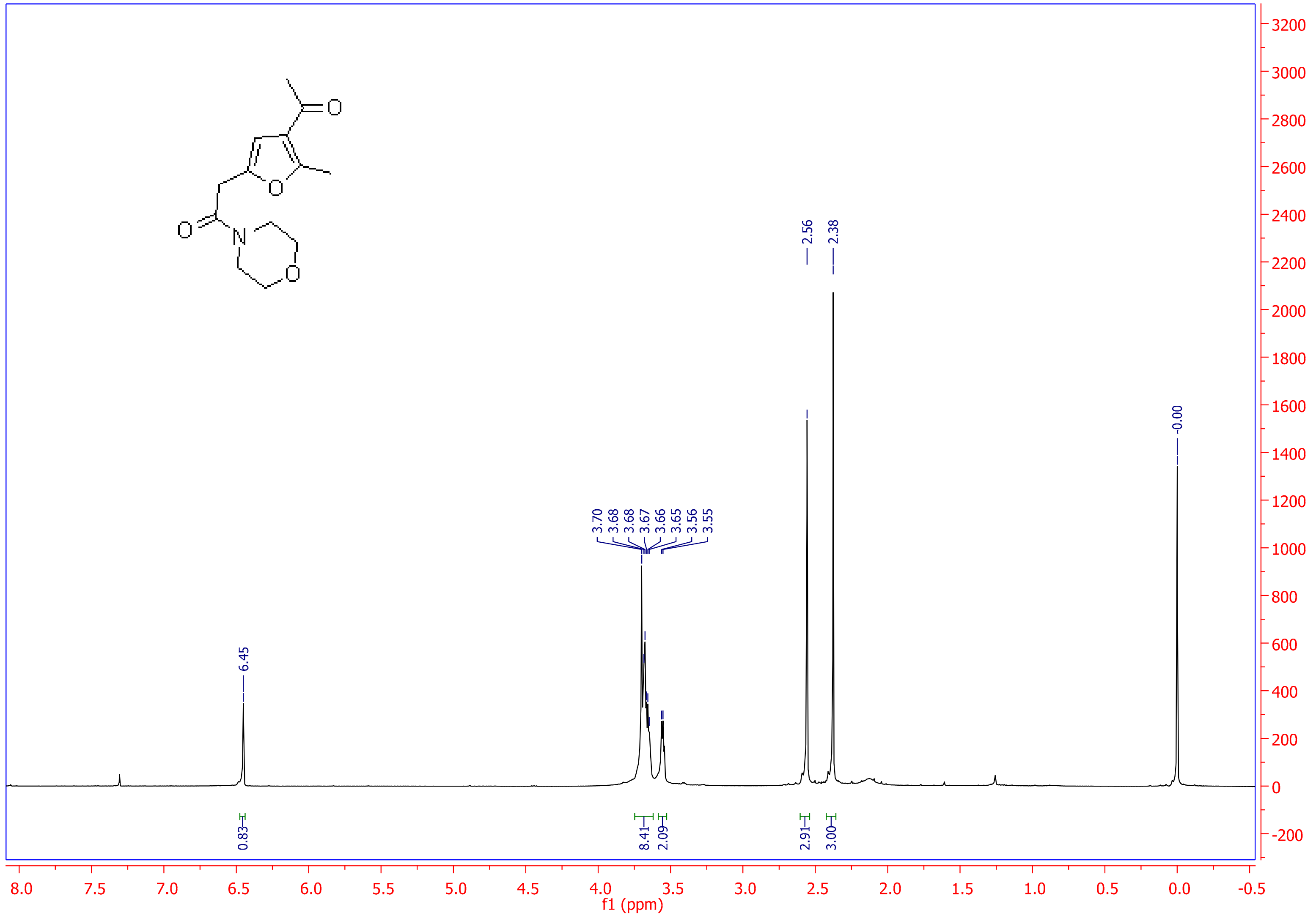

2-(4-Acetyl-5-methylfuran-2-yl)-1-morpholinoethan-1-one (3af)

^{13}C NMR (125 MHz $CDCl_3$)

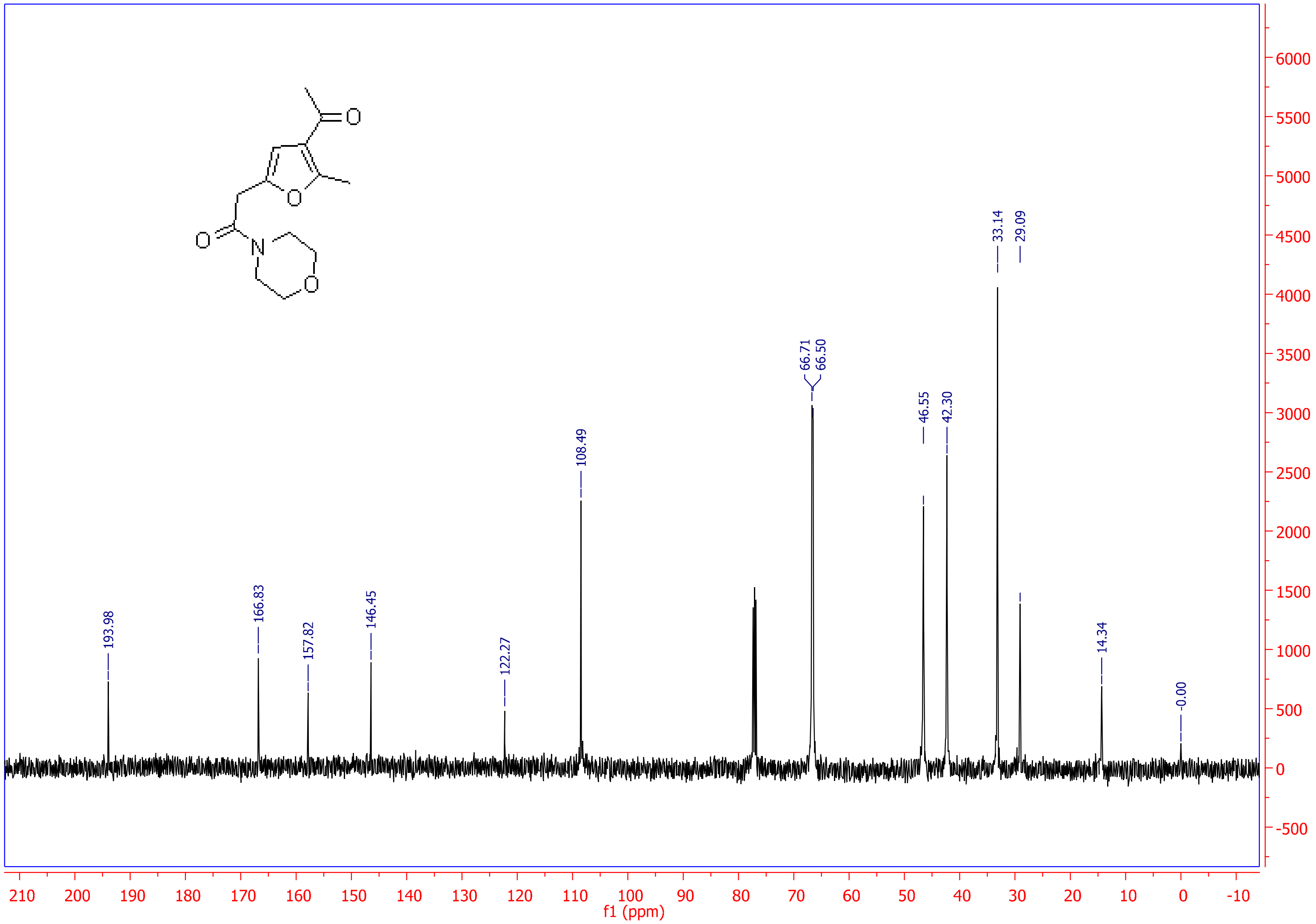

***N,N*-Diethyl-2-(5-ethyl-4-propionylfuran-2-yl)acetamide (3ba)**

^{1}H NMR (500 MHz $CDCl_3$)

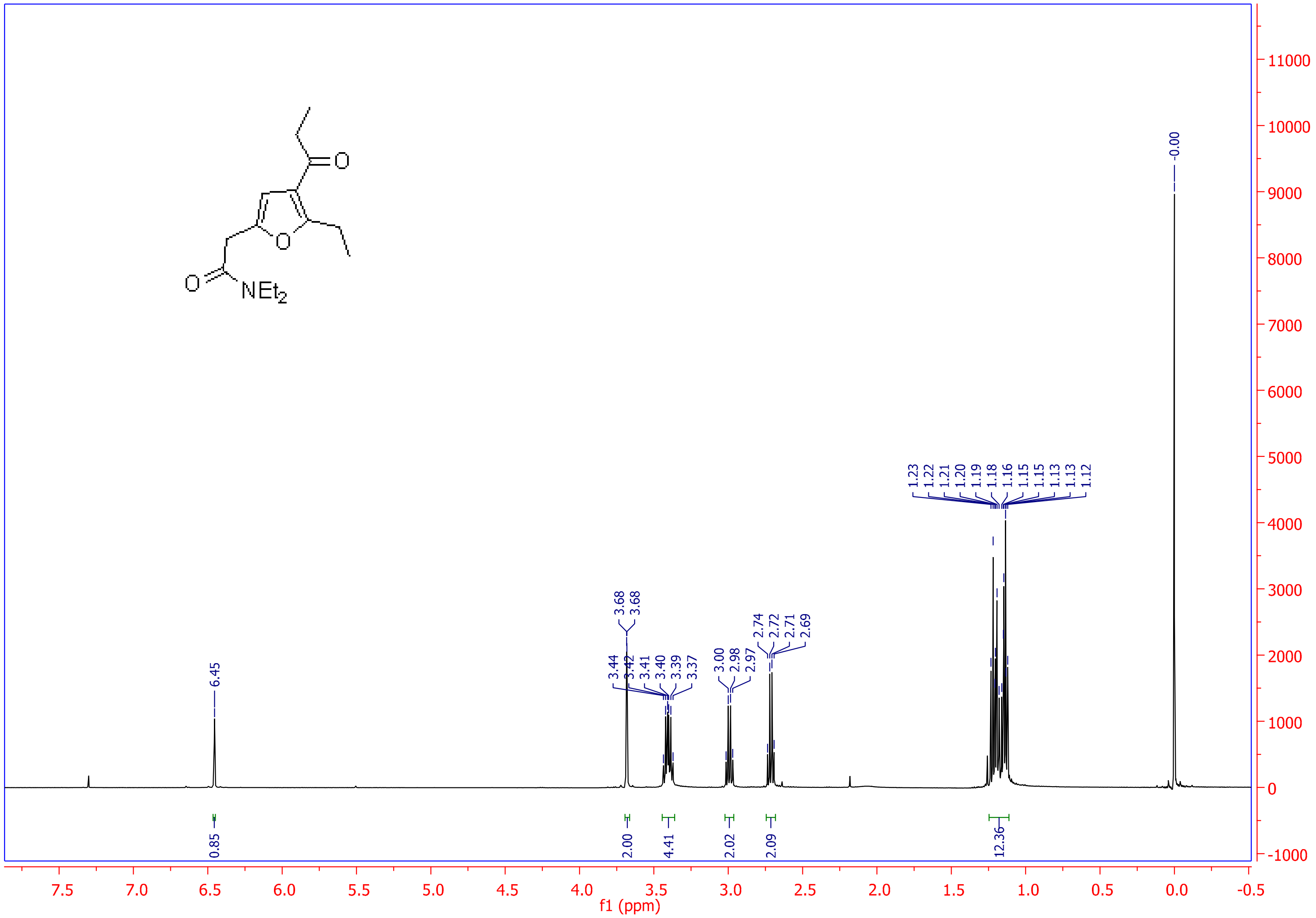

_N,N_-Diethyl-2-(5-ethyl-4-propionylfuran-2-yl)acetamide (3ba)

^{13}C NMR (125 MHz $CDCl_3$)

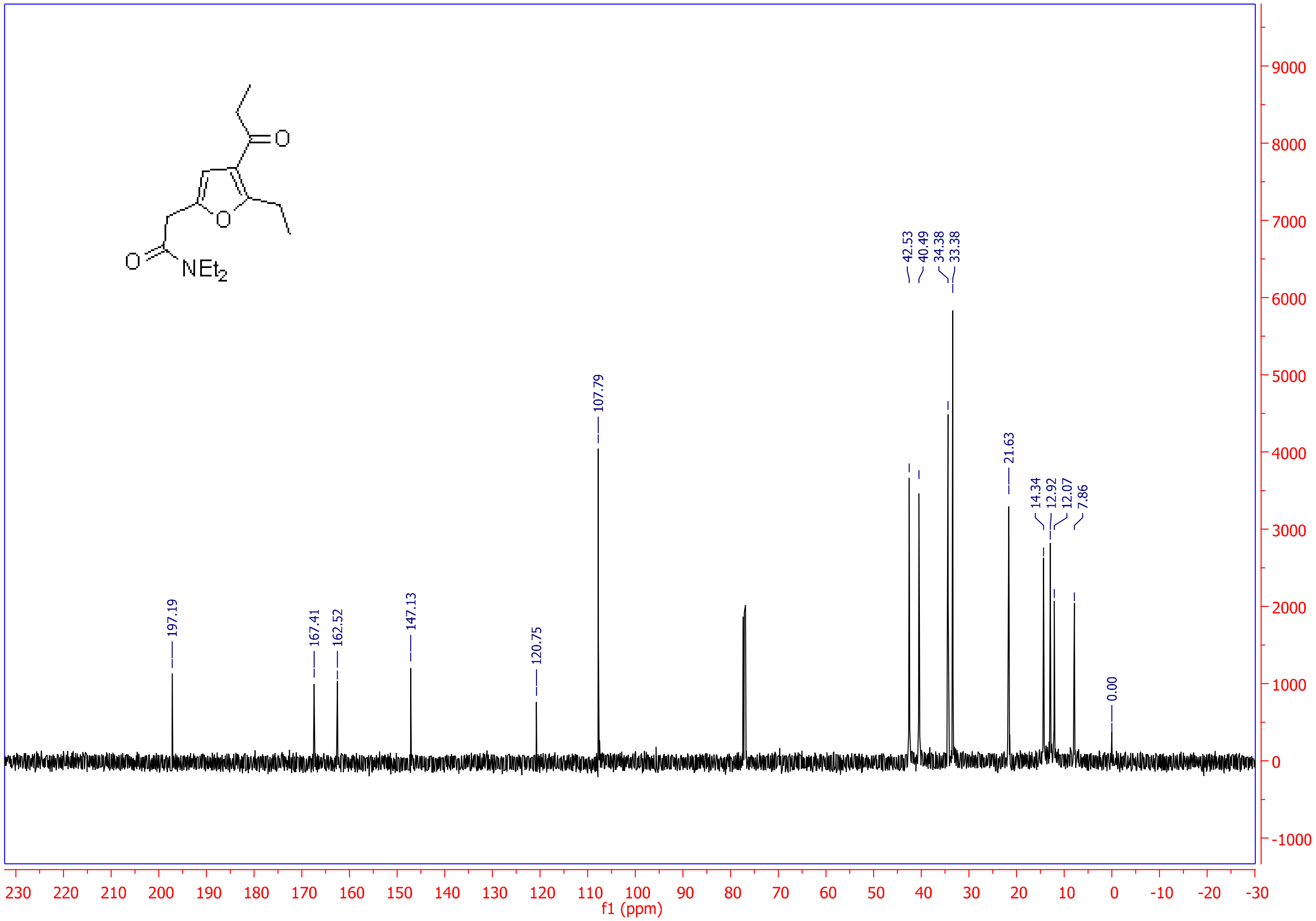

***N,N*-Diethyl-2-(4-oxo-4,5,6,7-tetrahydrobenzofuran-2-yl)acetamide (3ca)**

^{1}H NMR (500 MHz $CDCl_3$)

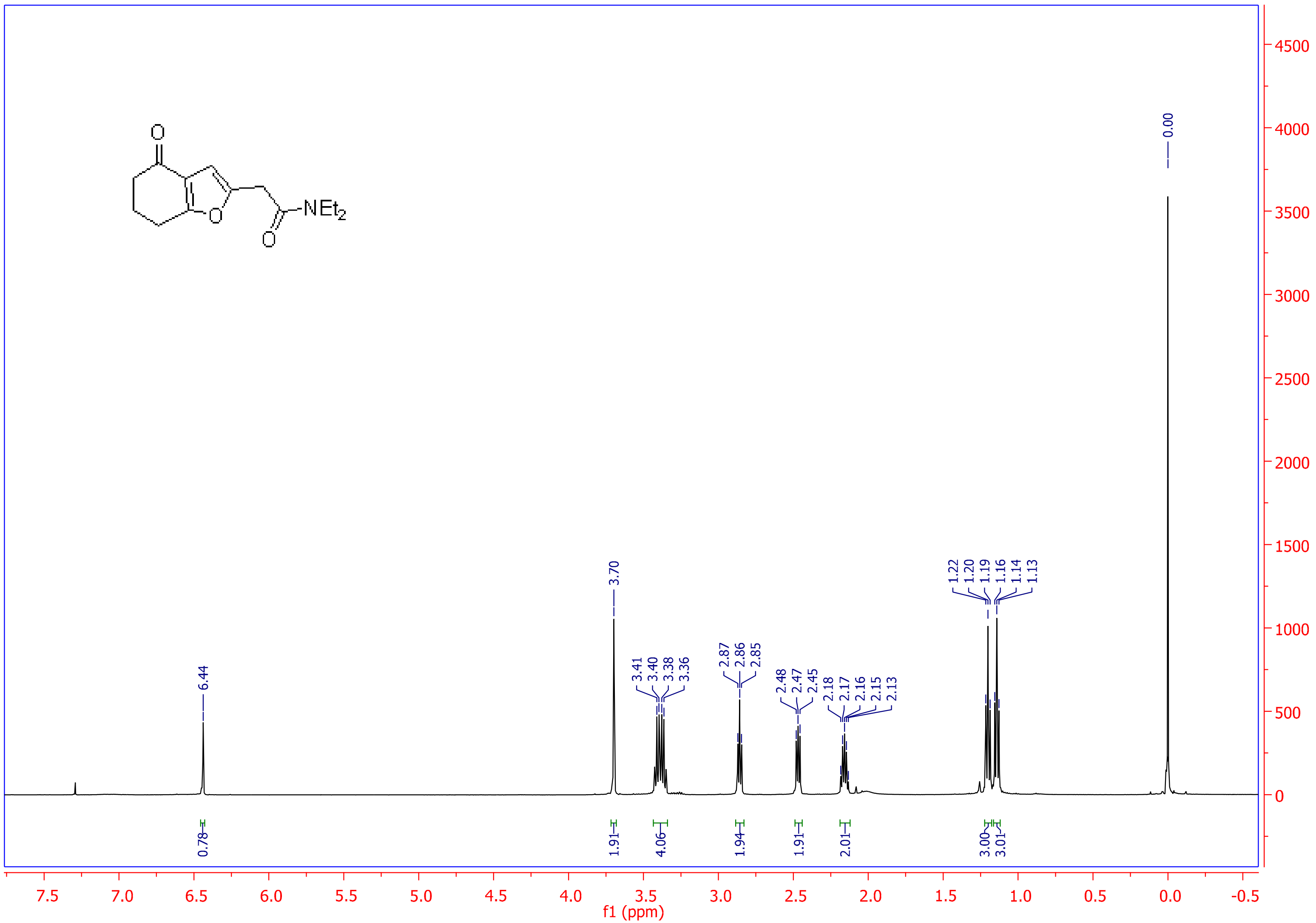

_N,N_-Diethyl-2-(4-oxo-4,5,6,7-tetrahydrobenzofuran-2-yl)acetamide (3ca)

^{13}C NMR (125 MHz $CDCl_3$)

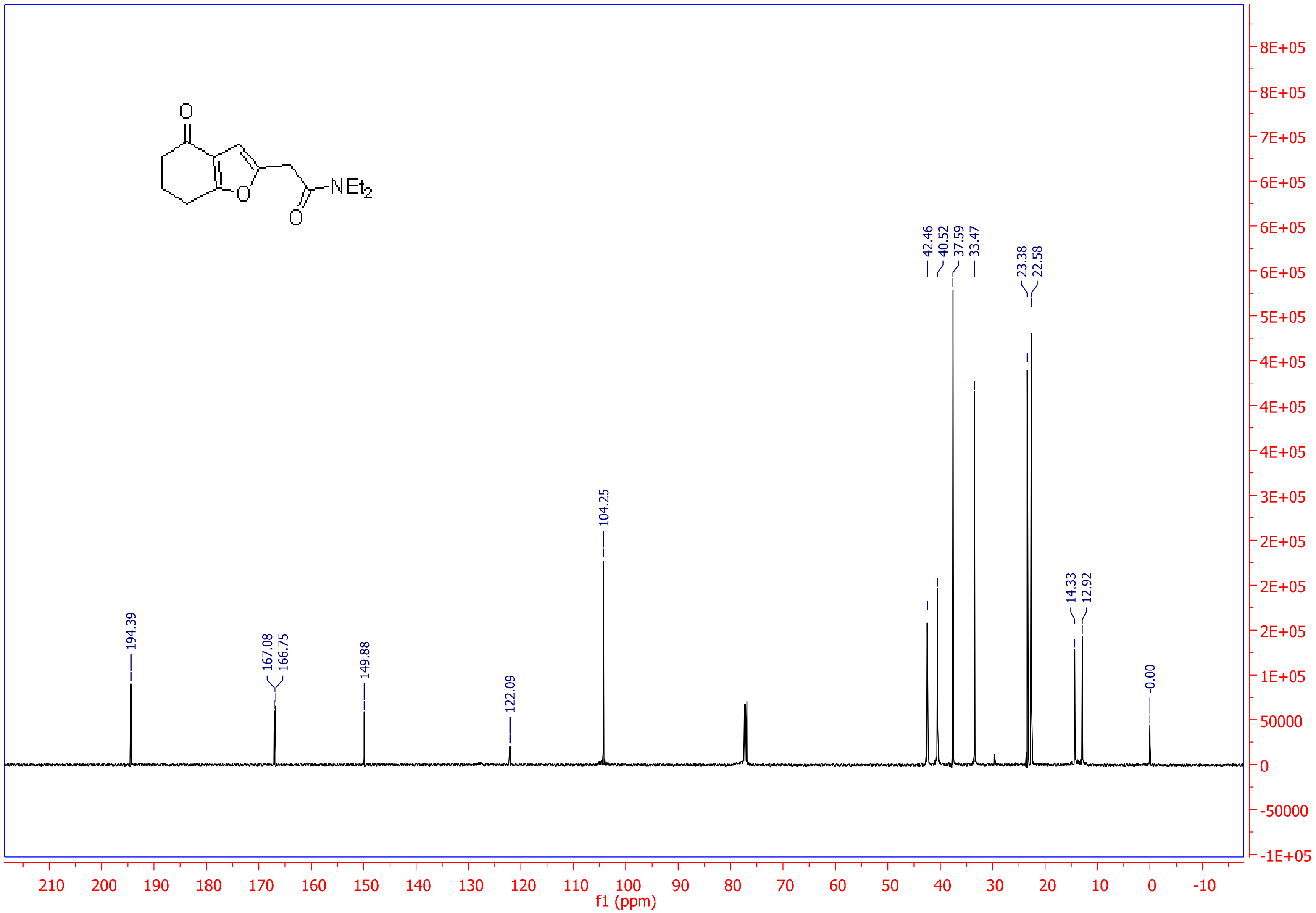

2-(4-Acetyl-3,5-dimethylfuran-2-yl)-1-morpholinoethan-1-one (3df)

^{1}H NMR (500 MHz $CDCl_3$)

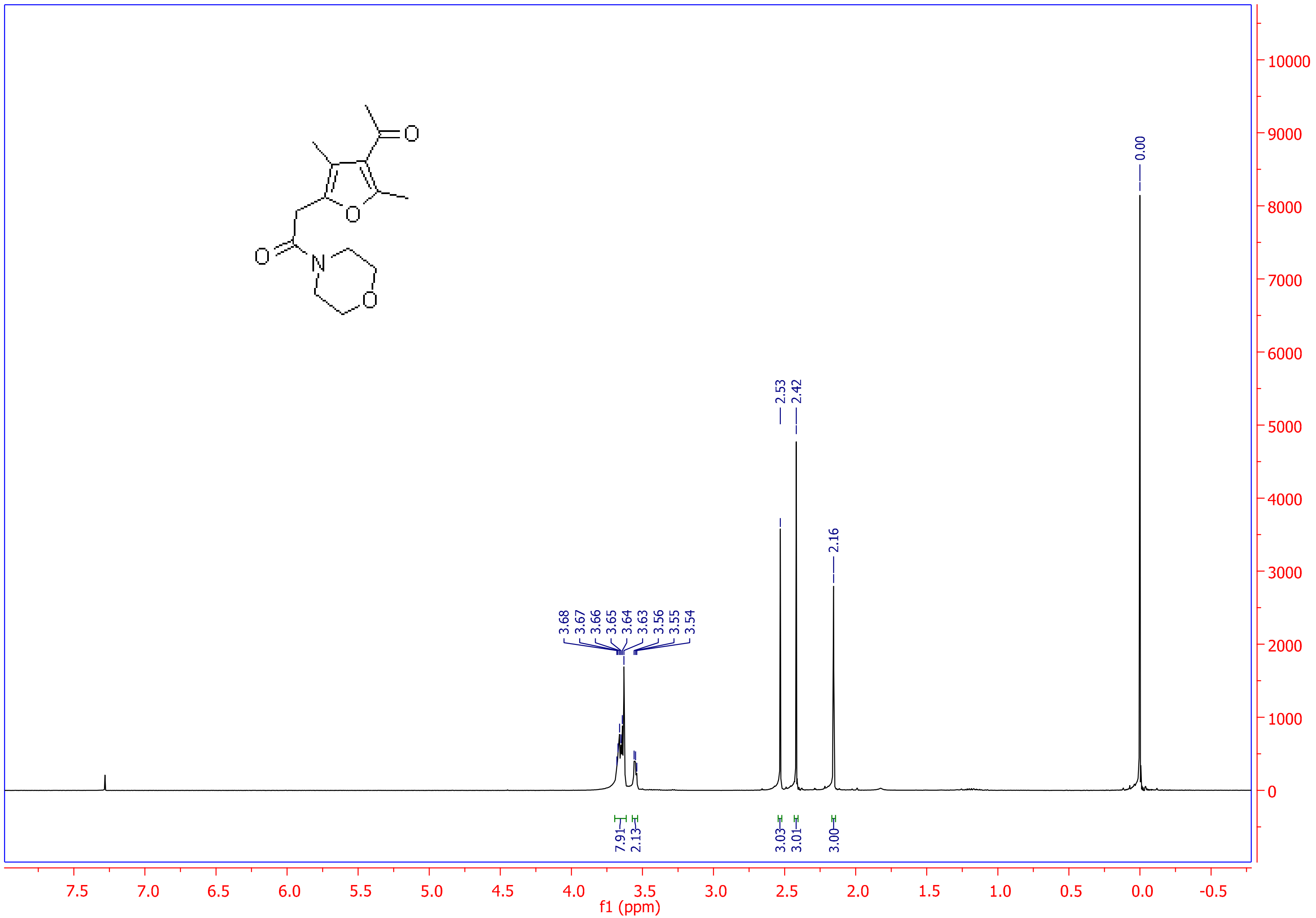

2-(4-Acetyl-3,5-dimethylfuran-2-yl)-1-morpholinoethan-1-one (3df)

^{13}C NMR (125 MHz $CDCl_3$)

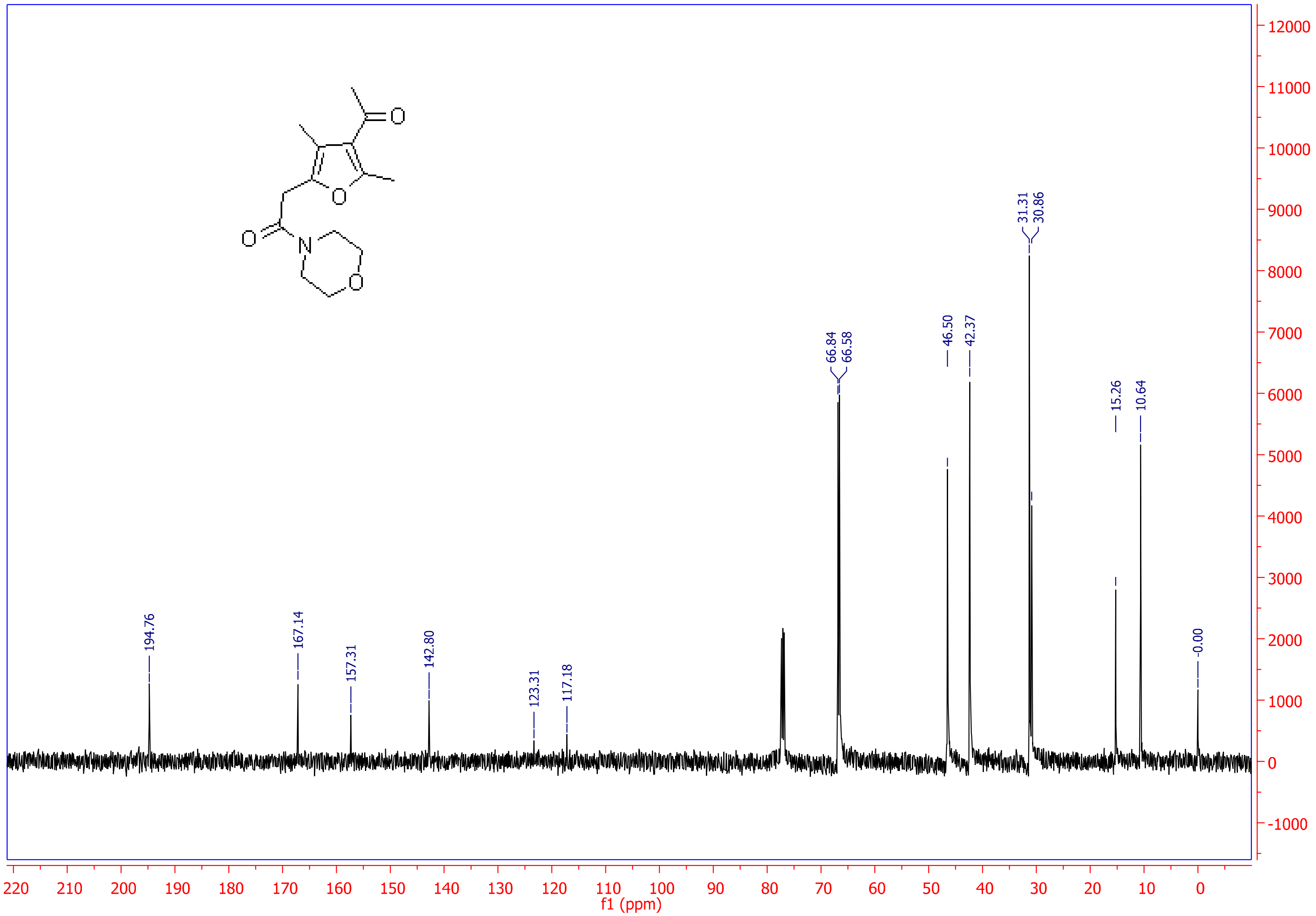

2-(4-Benzoyl-5-phenylfuran-2-yl)-*N*,*N*-diethylacetamide (3ea)

^{1}H NMR (500 MHz $CDCl_3$)

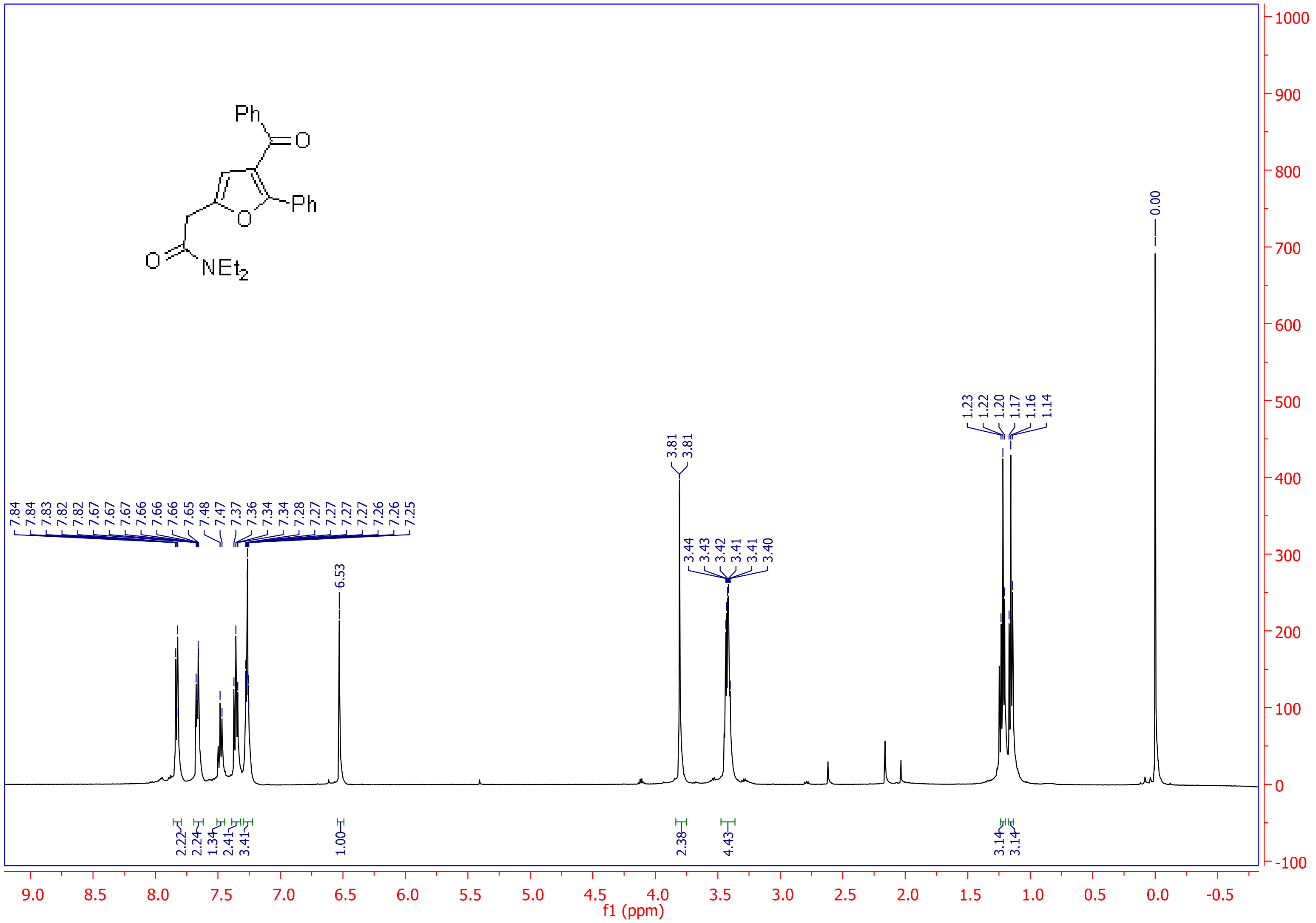

2-(4-Benzoyl-5-phenylfuran-2-yl)-*N*,*N*-diethylacetamide (3ea)

^{13}C NMR (125 MHz $CDCl_3$)

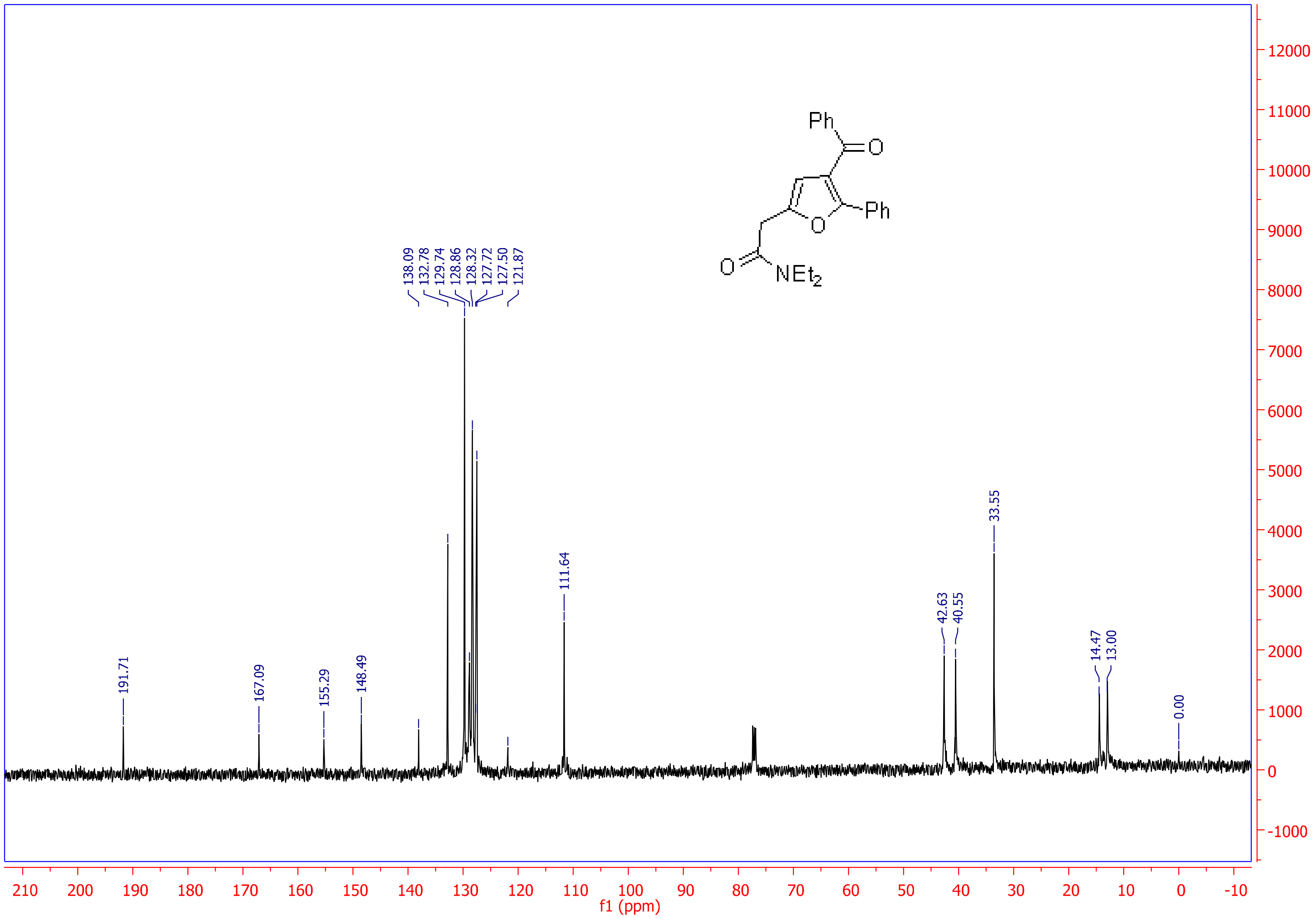

2-(4-Benzoyl-3,5-diphenylfuran-2-yl)-*N,N*-diethylacetamide (3fa)

^{1}H NMR (500 MHz $CDCl_3$)

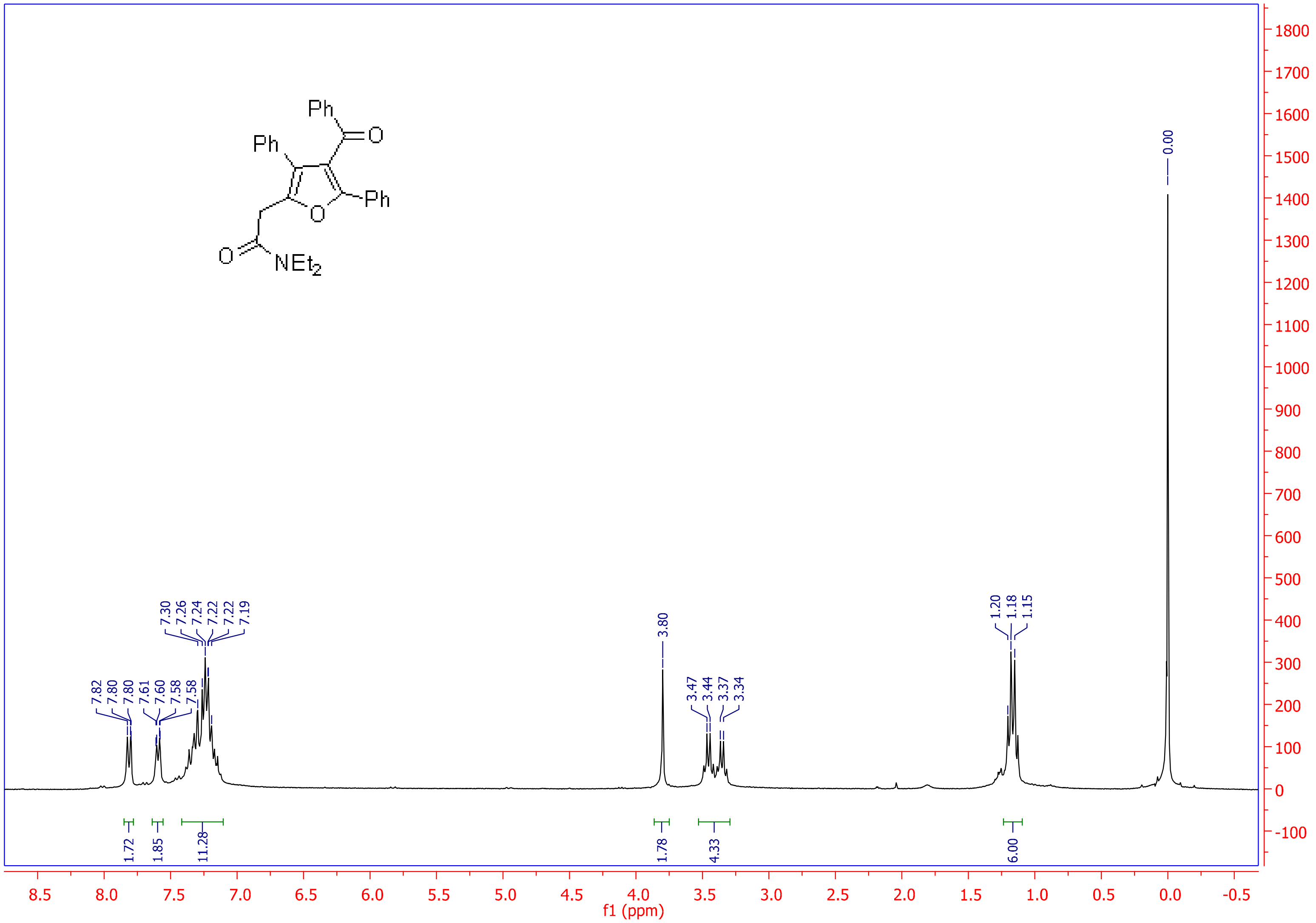

2-(4-Benzoyl-3,5-diphenylfuran-2-yl)-*N,N*-diethylacetamide (3fa)

^{13}C NMR (125 MHz $CDCl_3$)

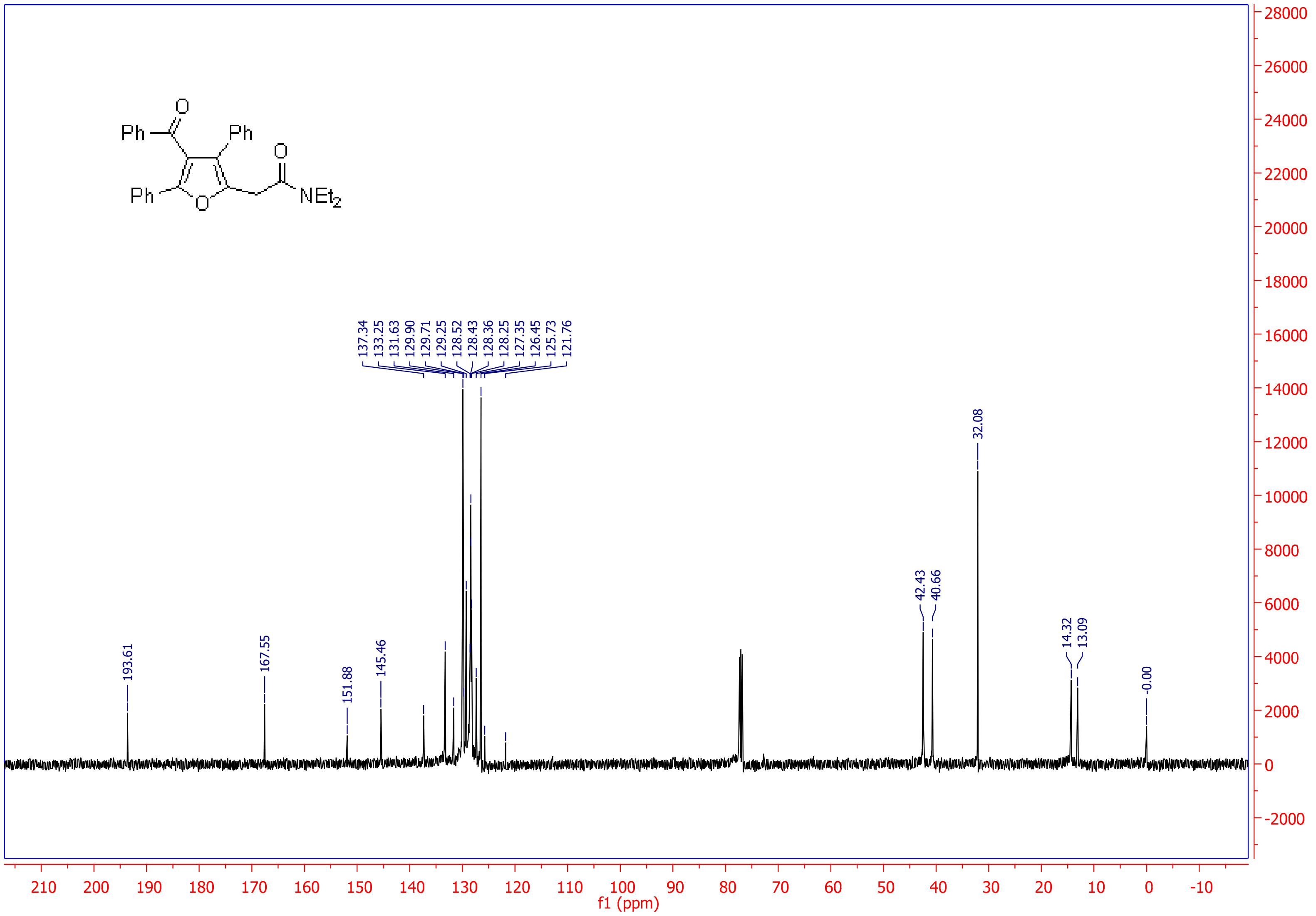

2-(4-Benzoyl-5-methylfuran-2-yl)-1-morpholinoethan-1-one (3gf).

^{1}H NMR (500 MHz CDCl$_3$)

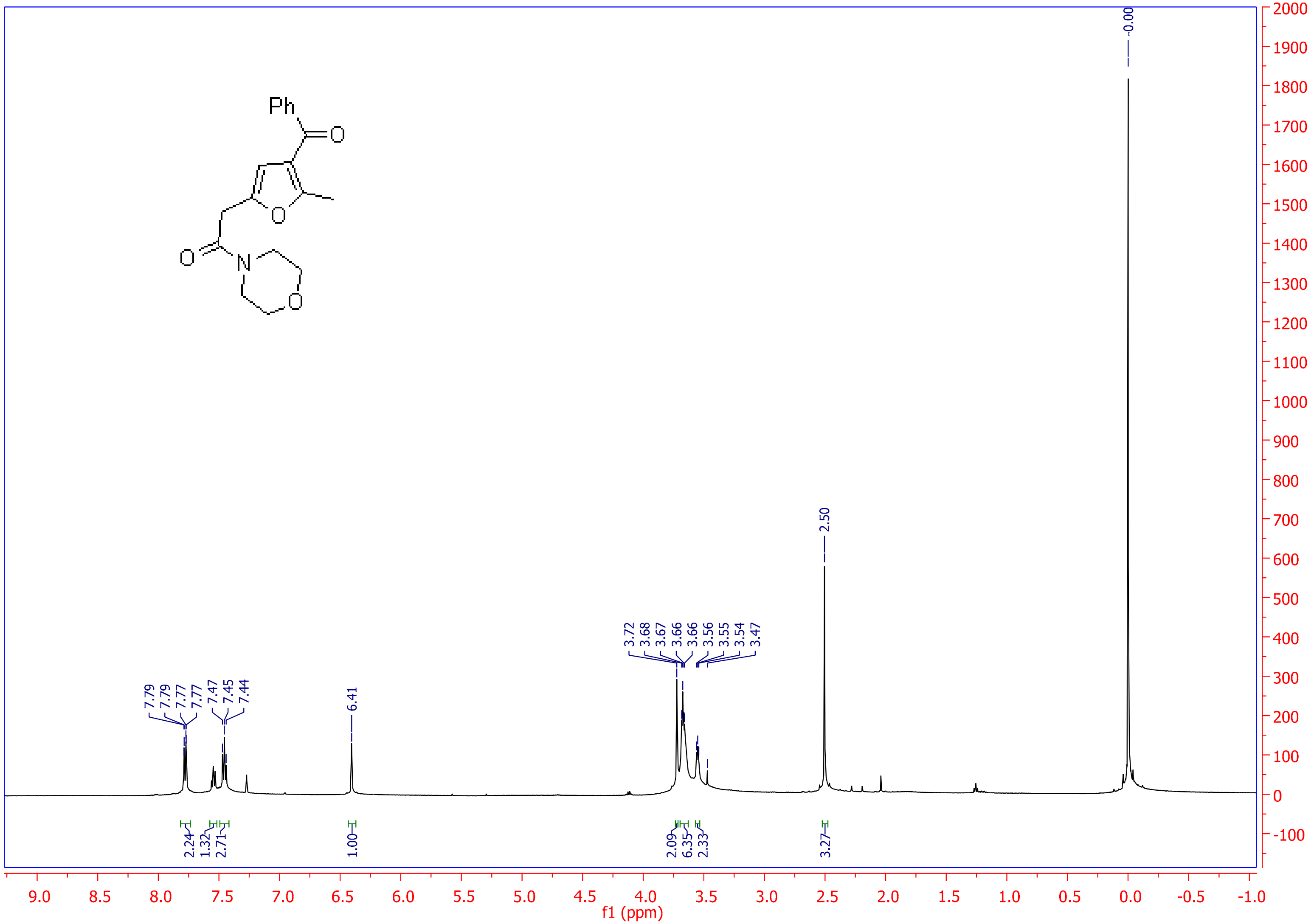

2-(4-Benzoyl-5-methylfuran-2-yl)-1-morpholinoethan-1-one (3gf).

^{13}C NMR (125 MHz $CDCl_3$)

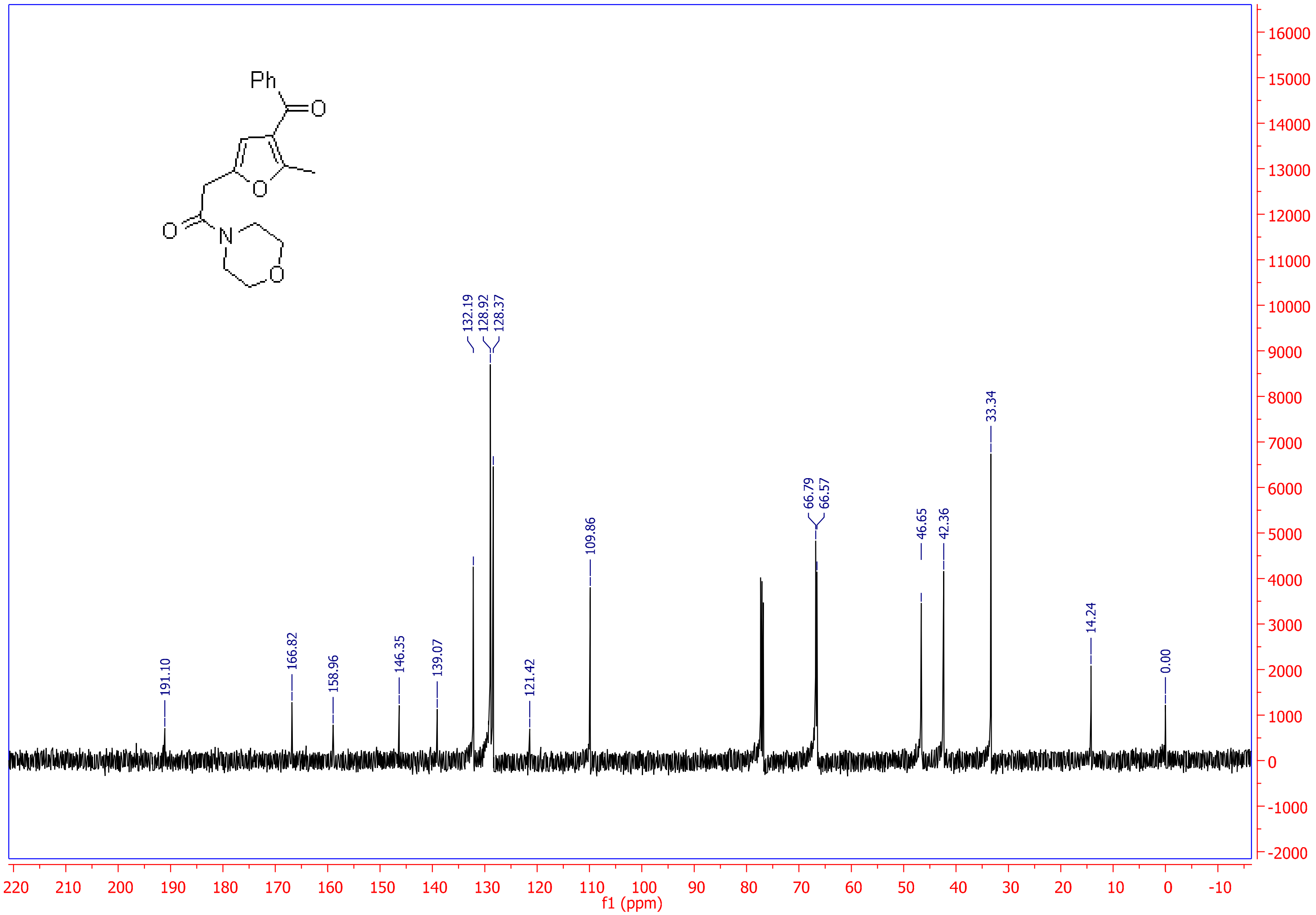

2-(4-Acetyl-5-phenylfuran-2-yl)-1-morpholinoethan-1-one (3gf’)

^{1}H NMR (500 MHz $CDCl_3$)

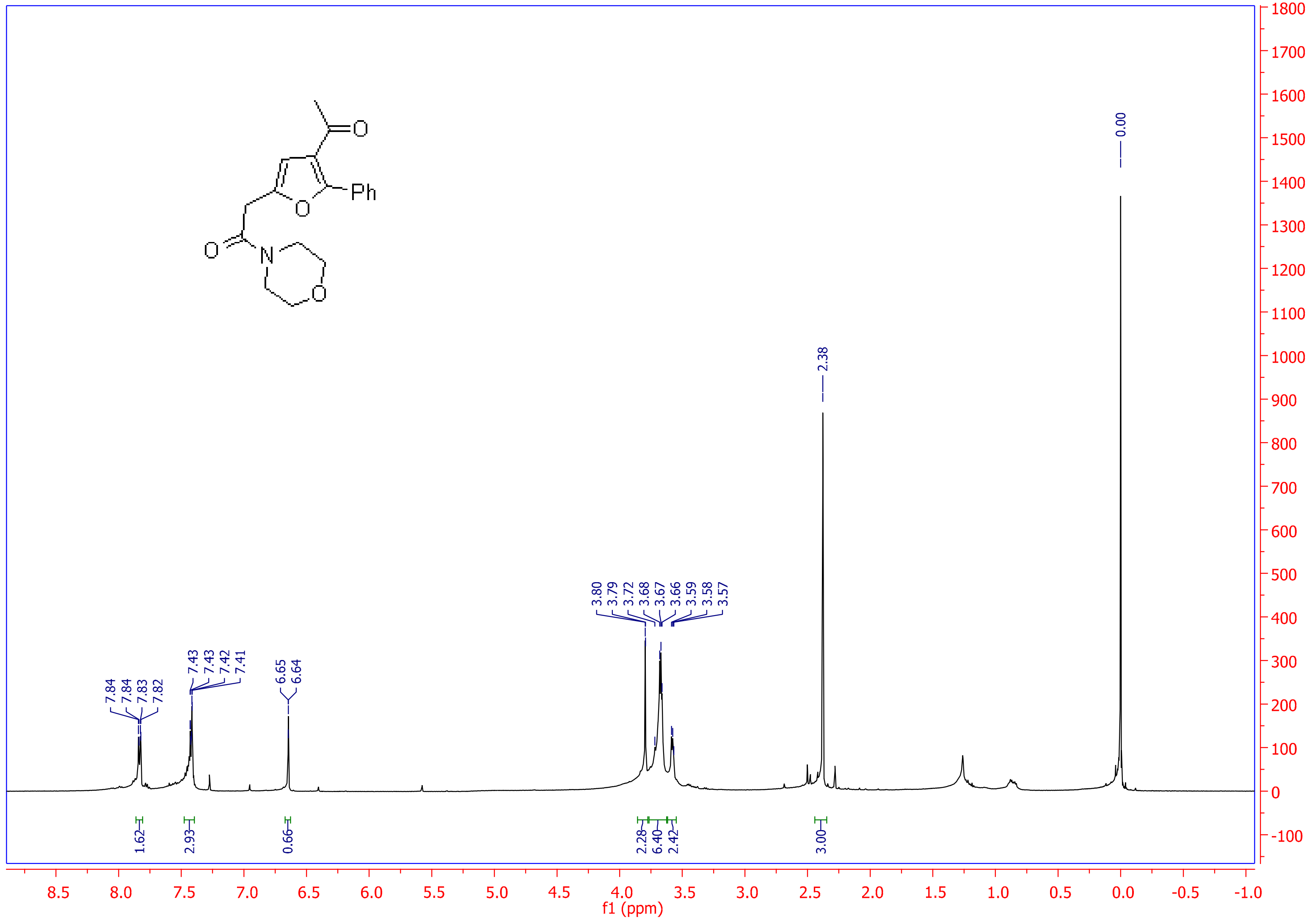

2-(4-Acetyl-5-phenylfuran-2-yl)-1-morpholinoethan-1-one (3gf').

^{13}C NMR (125 MHz CDCl$_3$)

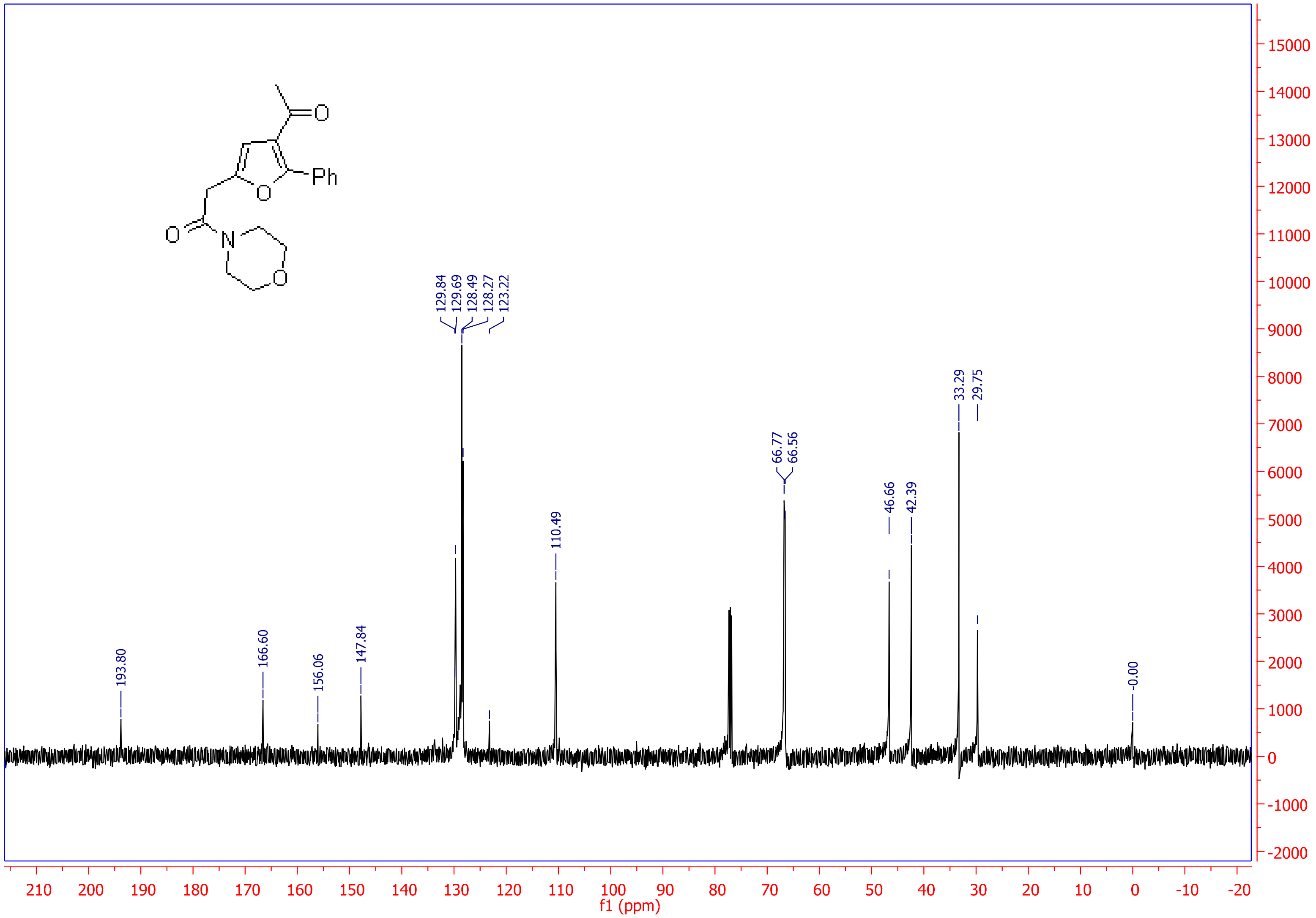

Methyl 5-(2-(diethylamino)-2-oxoethyl)-2-methylfuran-3-carboxylate (3ha).

^{1}H NMR (500 MHz $CDCl_3$)

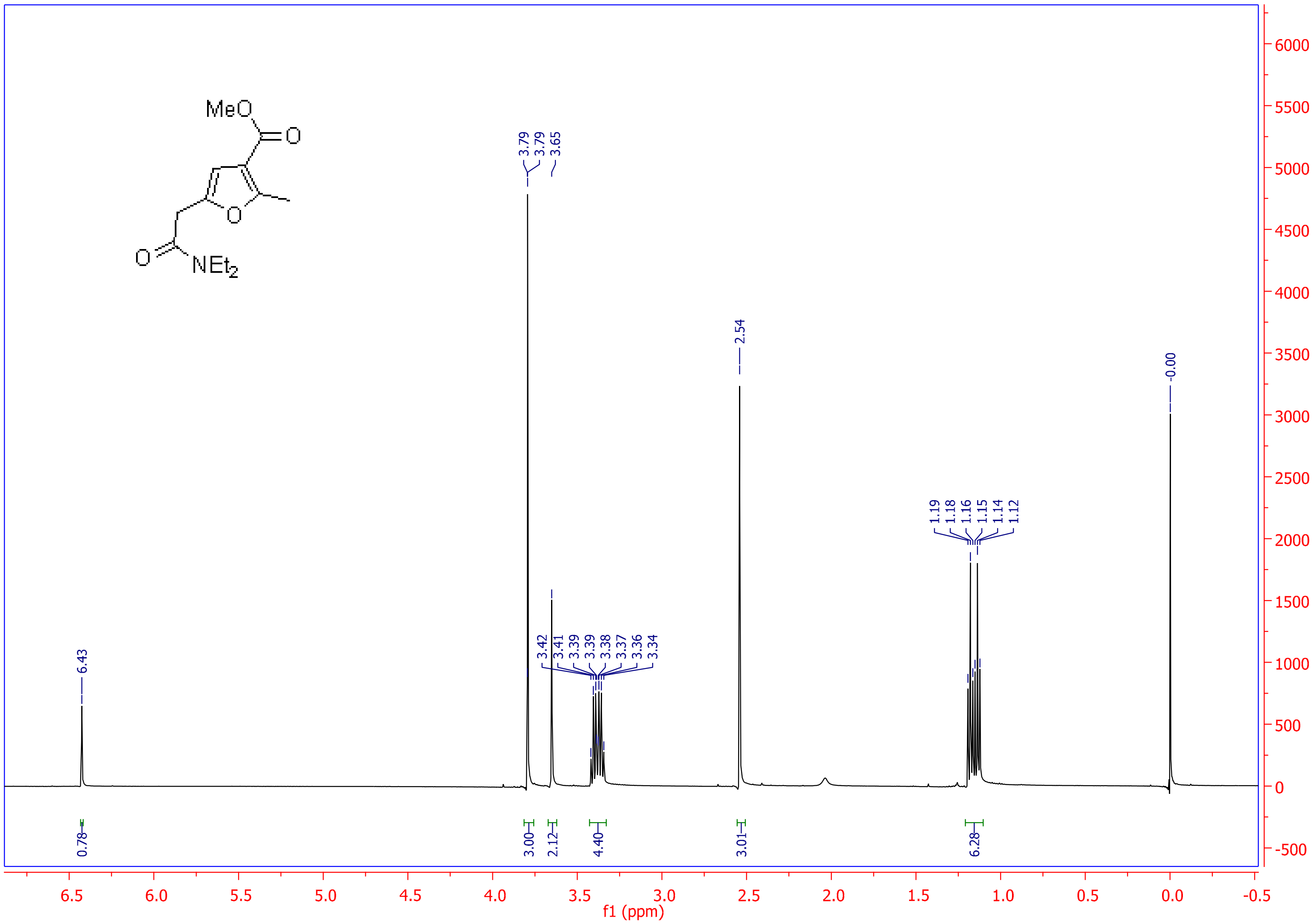

Methyl 5-(2-(diethylamino)-2-oxoethyl)-2-methylfuran-3-carboxylate (3ha).

^{13}C NMR (125 MHz CDCl$_3$)

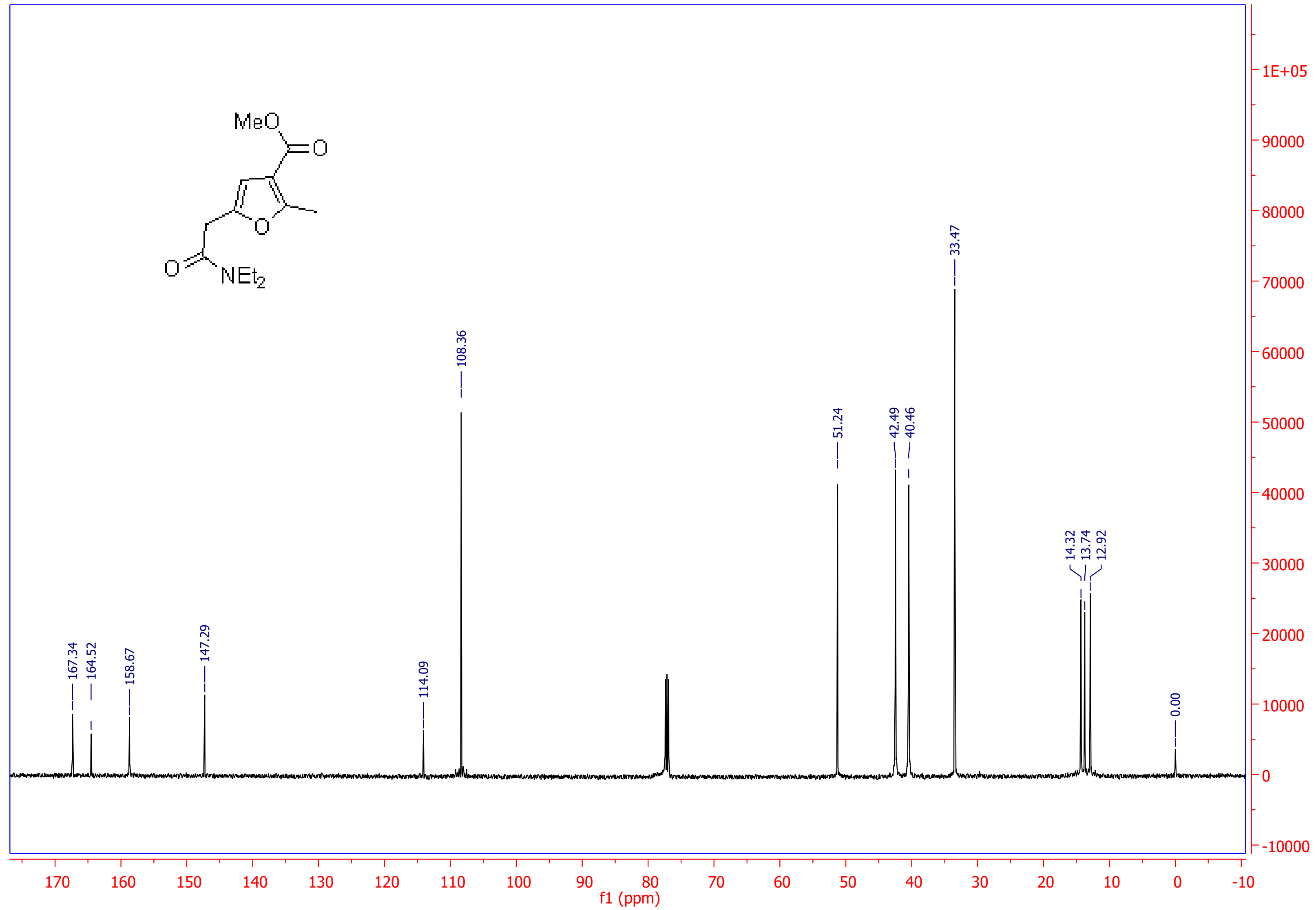

Ethyl 5-(2-(diethylamino)-2-oxoethyl)-2-methylfuran-3-carboxylate (3ia)

^{1}H NMR (500 MHz CDCl$_3$)

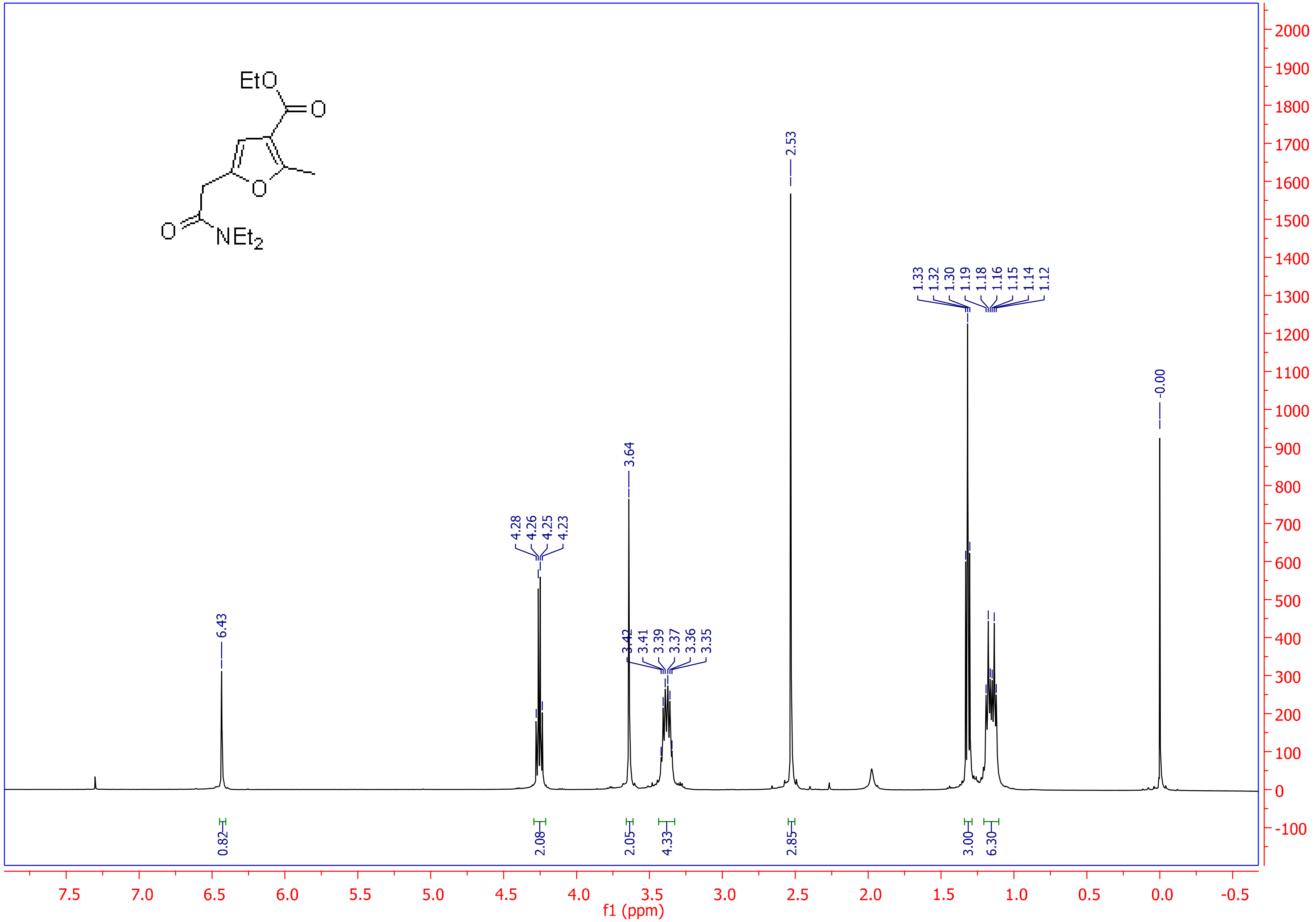

Ethyl 5-(2-(diethylamino)-2-oxoethyl)-2-methylfuran-3-carboxylate (3ia)

^{13}C NMR (125 MHz $CDCl_3$)

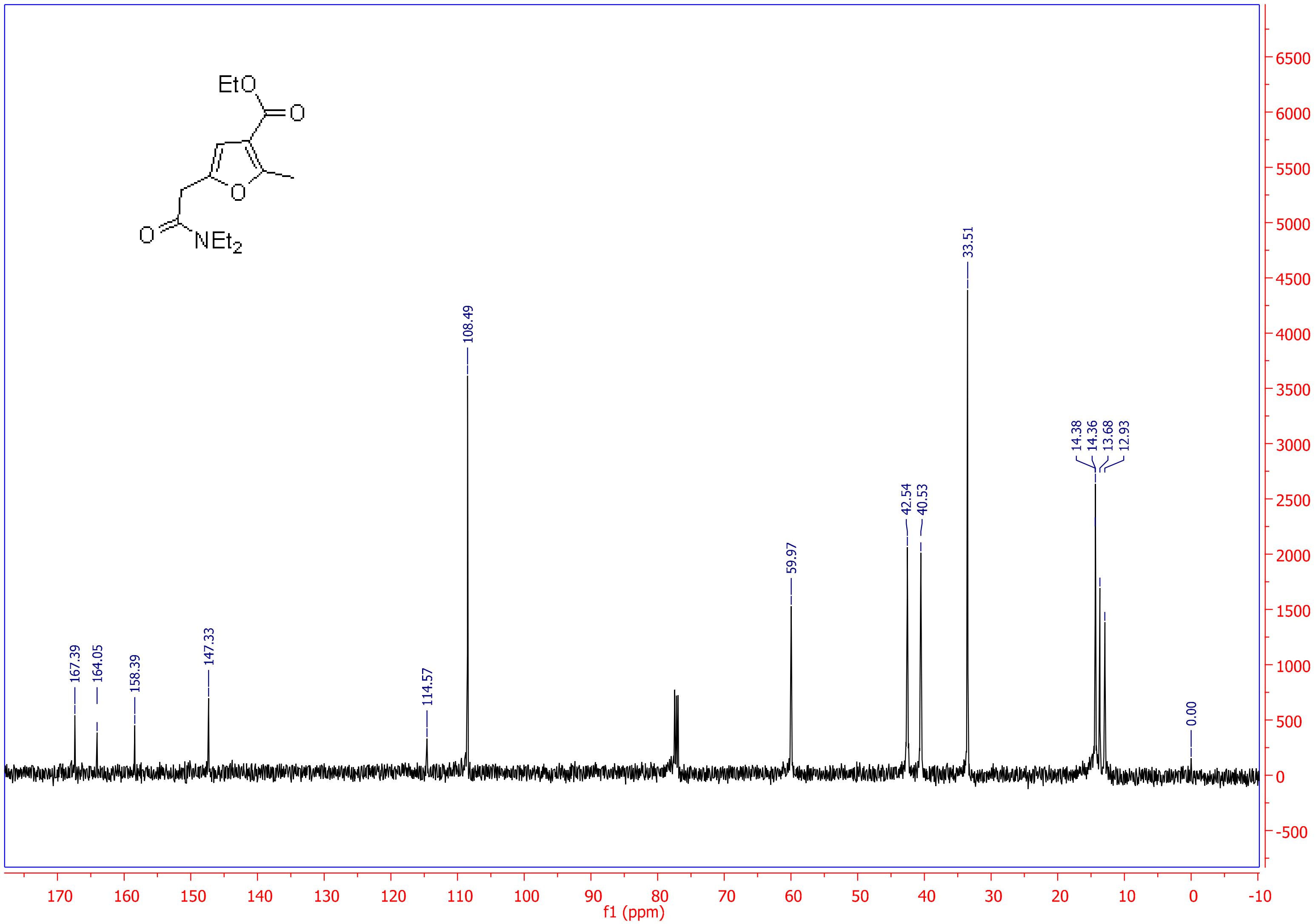

Ethyl 5-(2-(diethylamino)-2-oxoethyl)-2-propylfuran-3-carboxylate (3ja).

^{1}H NMR (500 MHz CDCl$_3$)

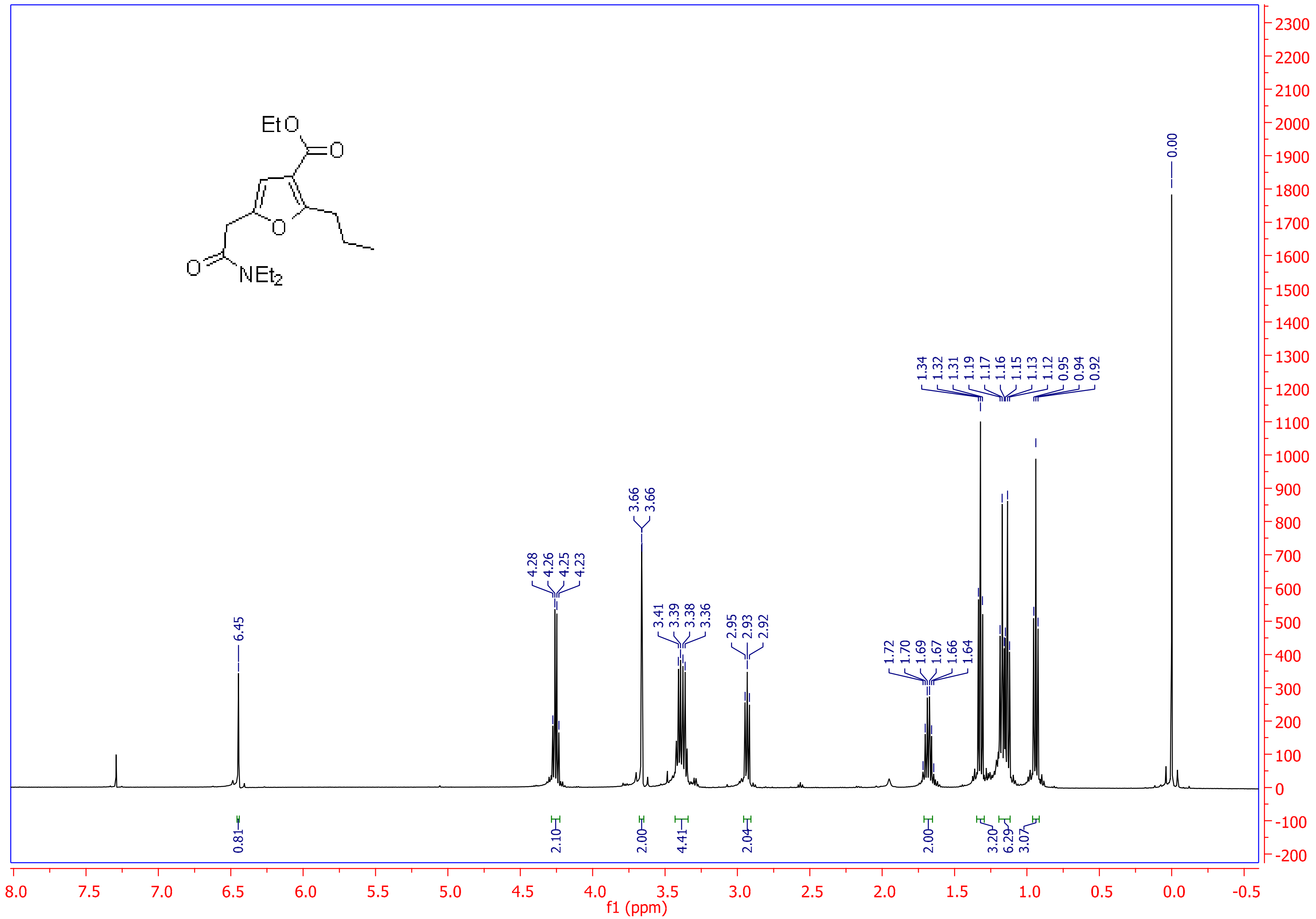

Ethyl 5-(2-(diethylamino)-2-oxoethyl)-2-propylfuran-3-carboxylate (3ja).

^{13}C NMR (125 MHz $CDCl_3$)

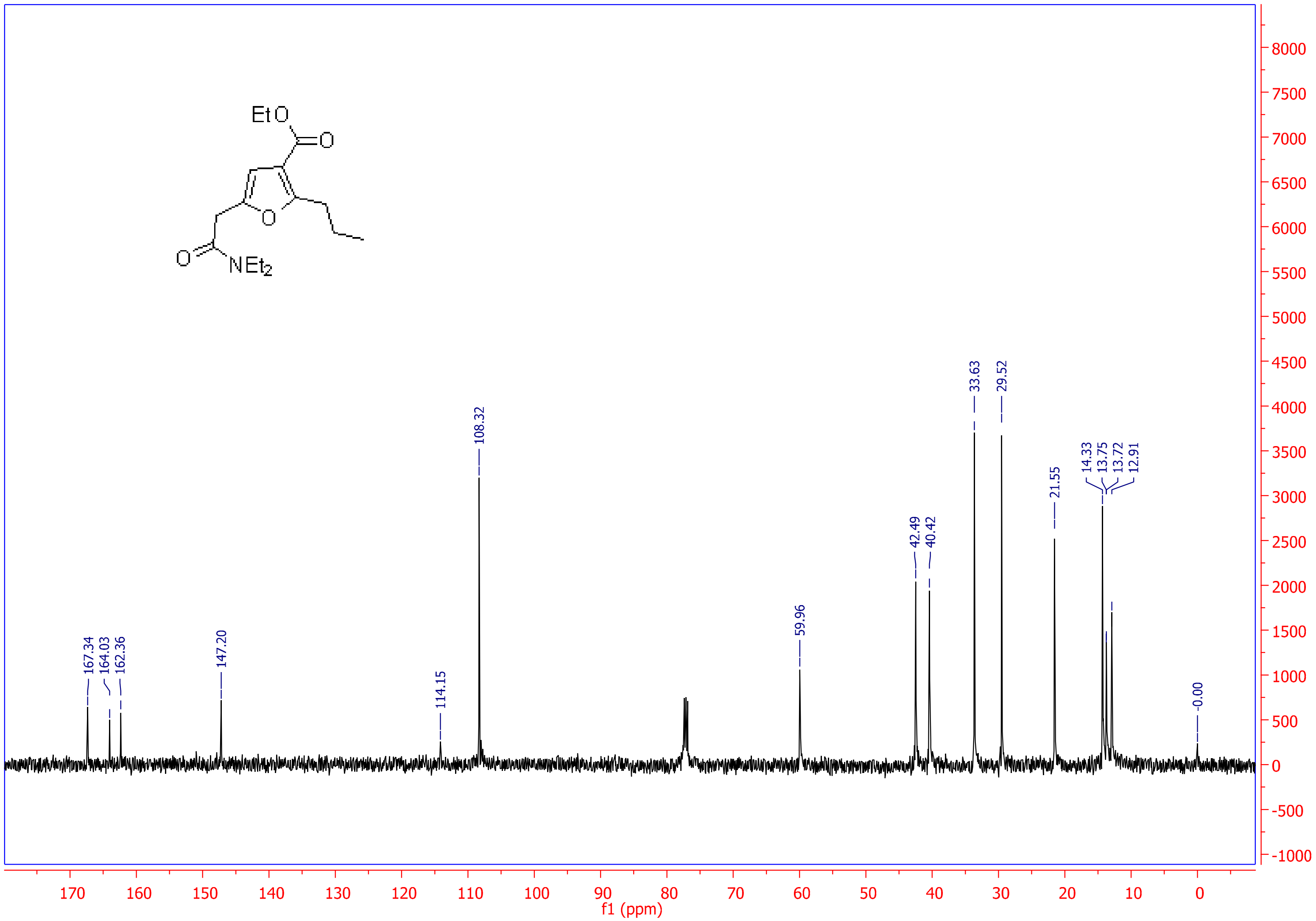

Ethyl 5-(2-(diethylamino)-2-oxoethyl)-2-isopropylfuran-3-carboxylate (3ka).

^{1}H NMR (500 MHz CDCl$_3$)

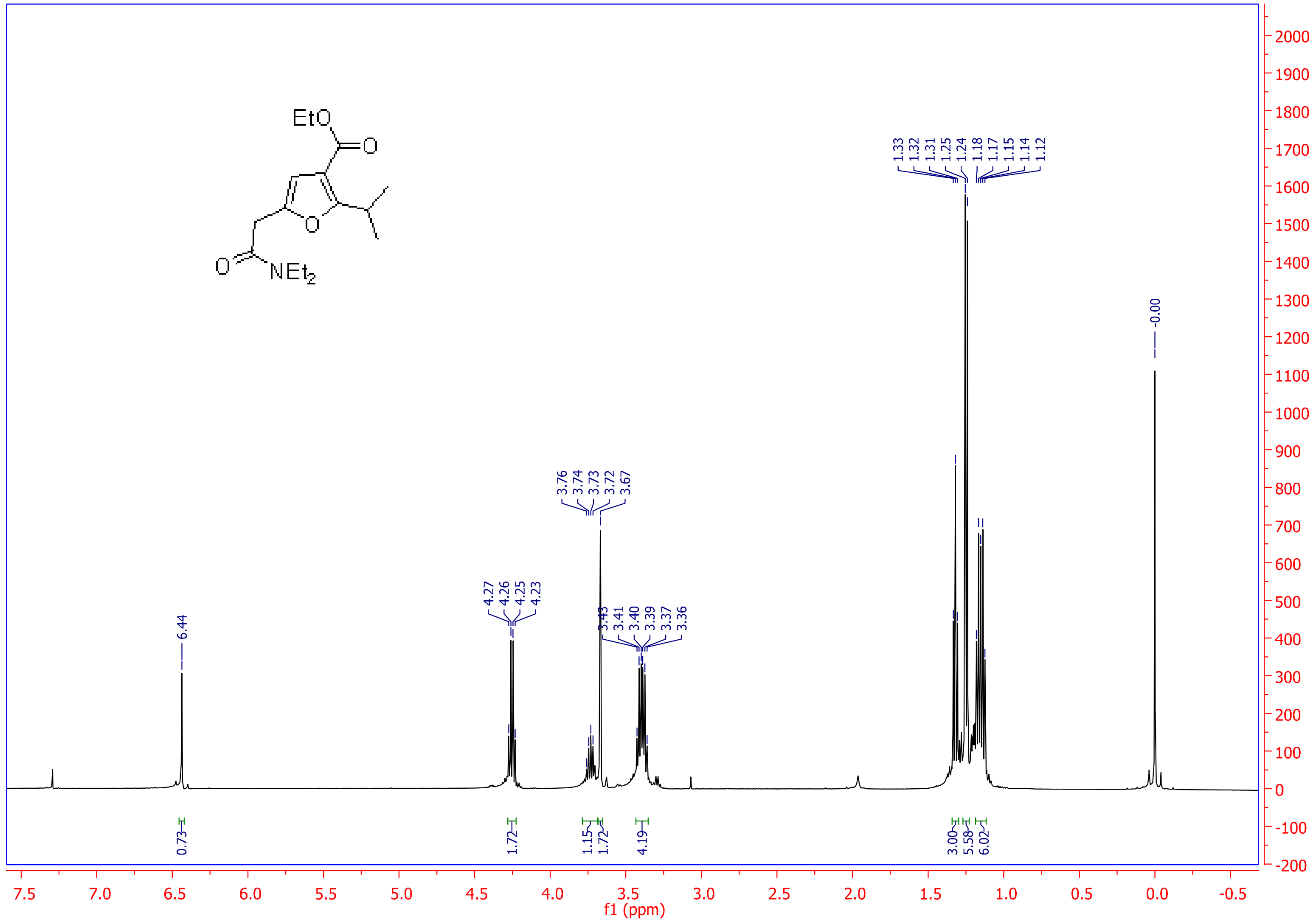

Ethyl 5-(2-(diethylamino)-2-oxoethyl)-2-isopropylfuran-3-carboxylate (3ka).

^{13}C NMR (125 MHz CDCl$_3$)

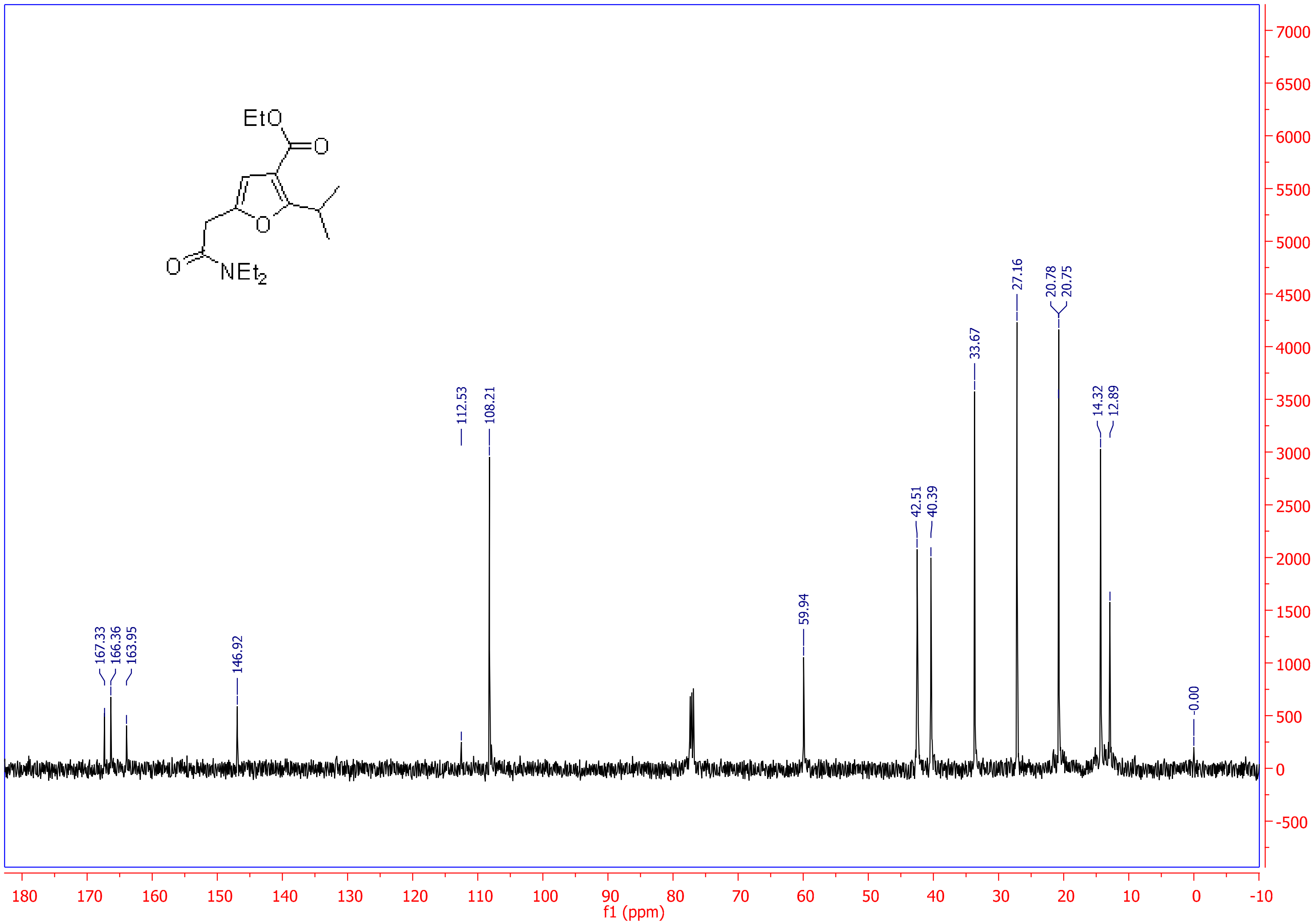

Benzyl 5-(2-(diethylamino)-2-oxoethyl)-2-methylfuran-3-carboxylate (3la).

^{1}H NMR (500 MHz $CDCl_3$)

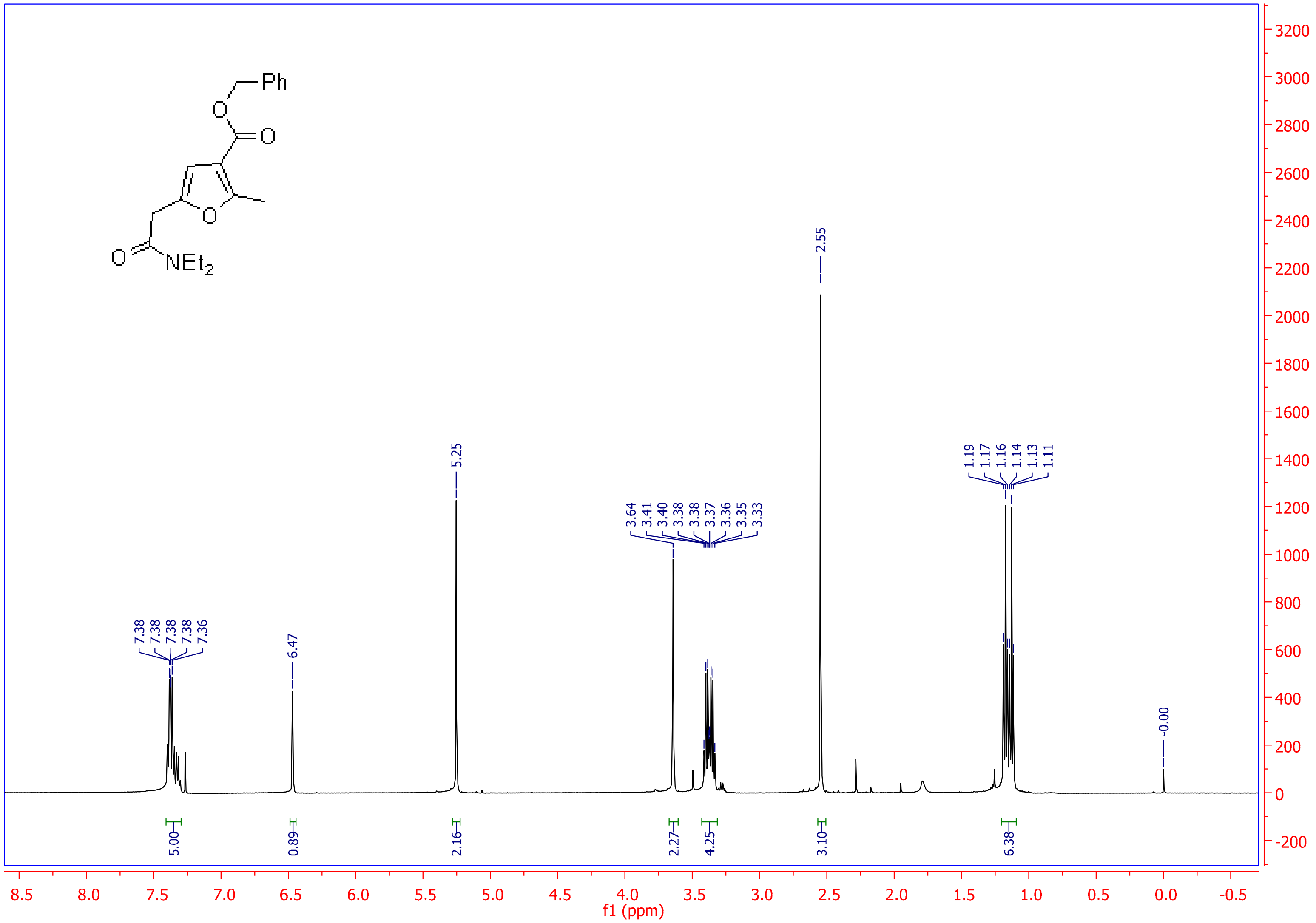

Benzyl 5-(2-(diethylamino)-2-oxoethyl)-2-methylfuran-3-carboxylate (3la).

^{13}C NMR (125 MHz $CDCl_3$)

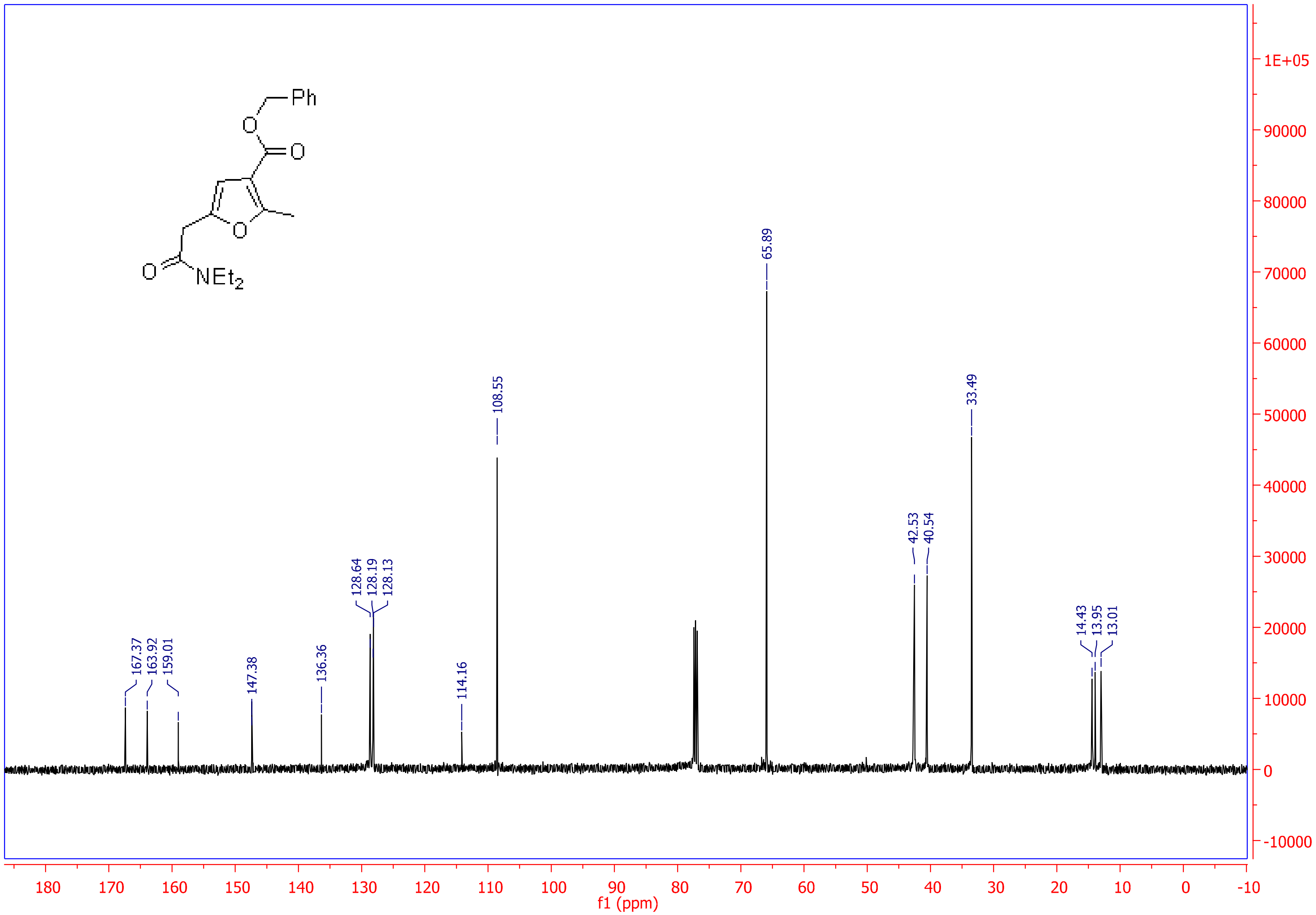

5-(2-(Diethylamino)-2-oxoethyl)-*N*,*N*-diethyl-2-methylfuran-3-carboxamide (3ma)

^{1}H NMR (500 MHz CDCl$_3$)

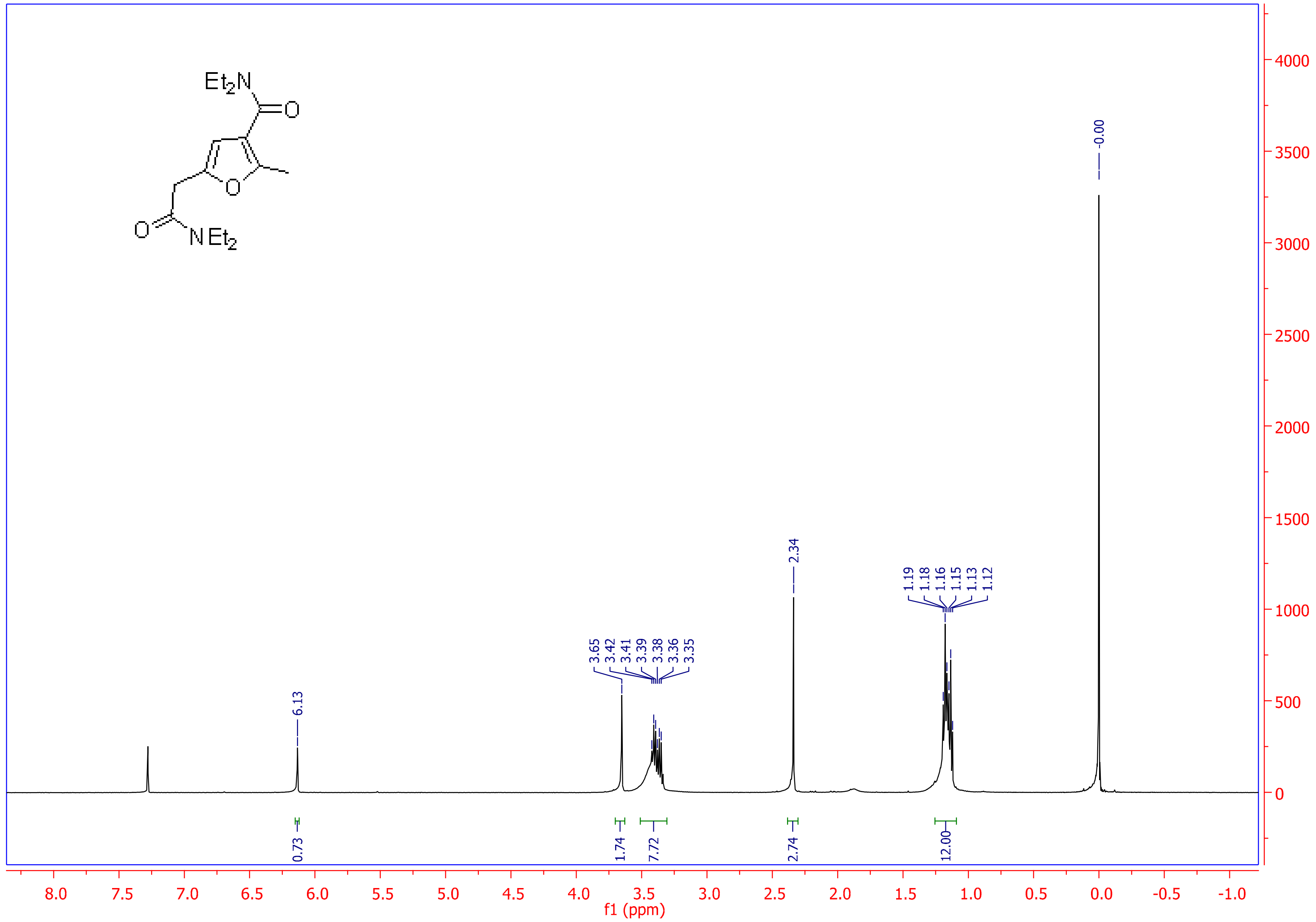

5-(2-(Diethylamino)-2-oxoethyl)-*N*,*N*-diethyl-2-methylfuran-3-carboxamide (3ma)

^{13}C NMR (125 MHz CDCl$_3$)

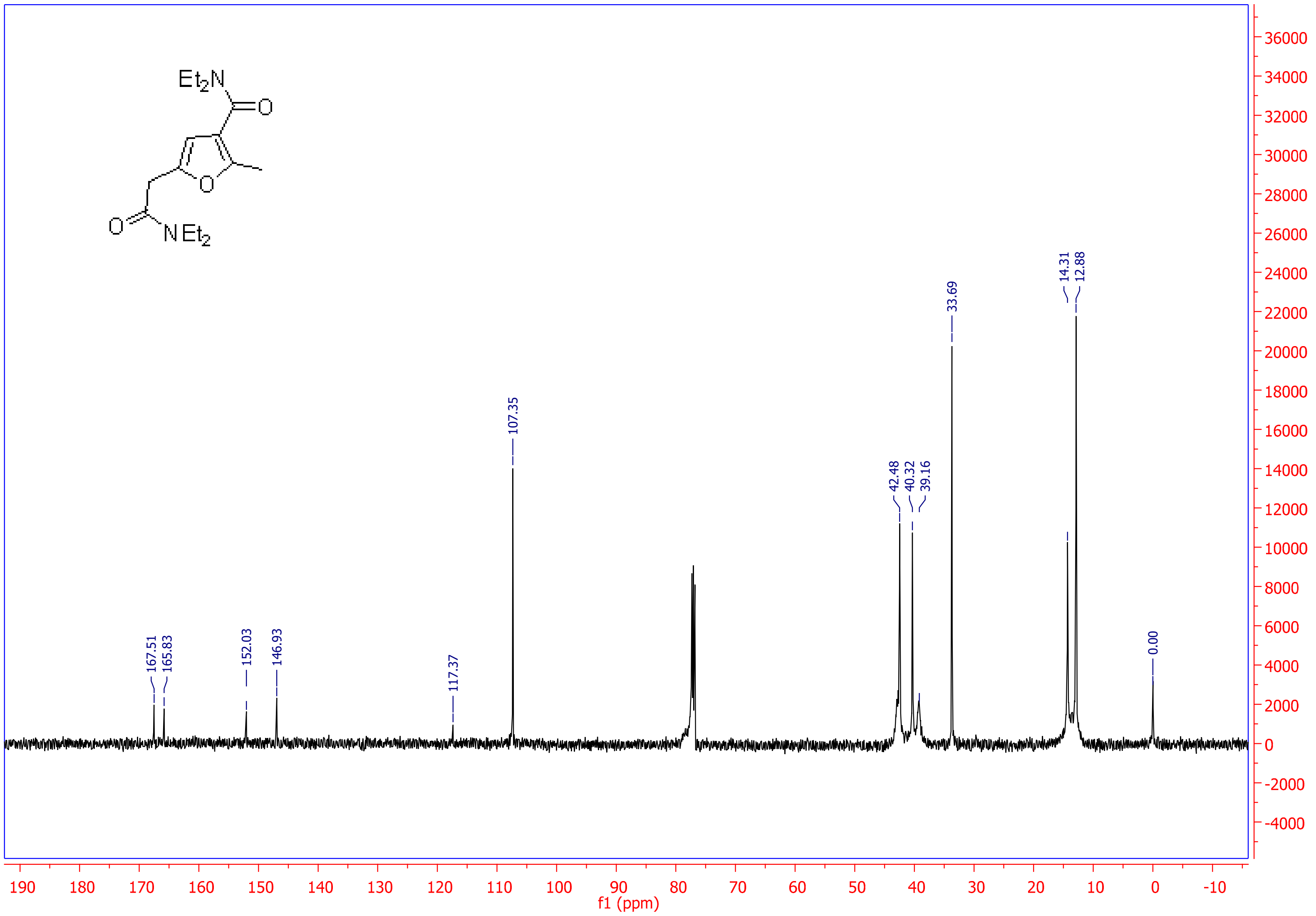

***N,N*-Dibutyl-2-(5-phenyl-4-tosylfuran-2-yl)acetamide (3nc)**

^{1}H NMR (500 MHz $CDCl_3$)

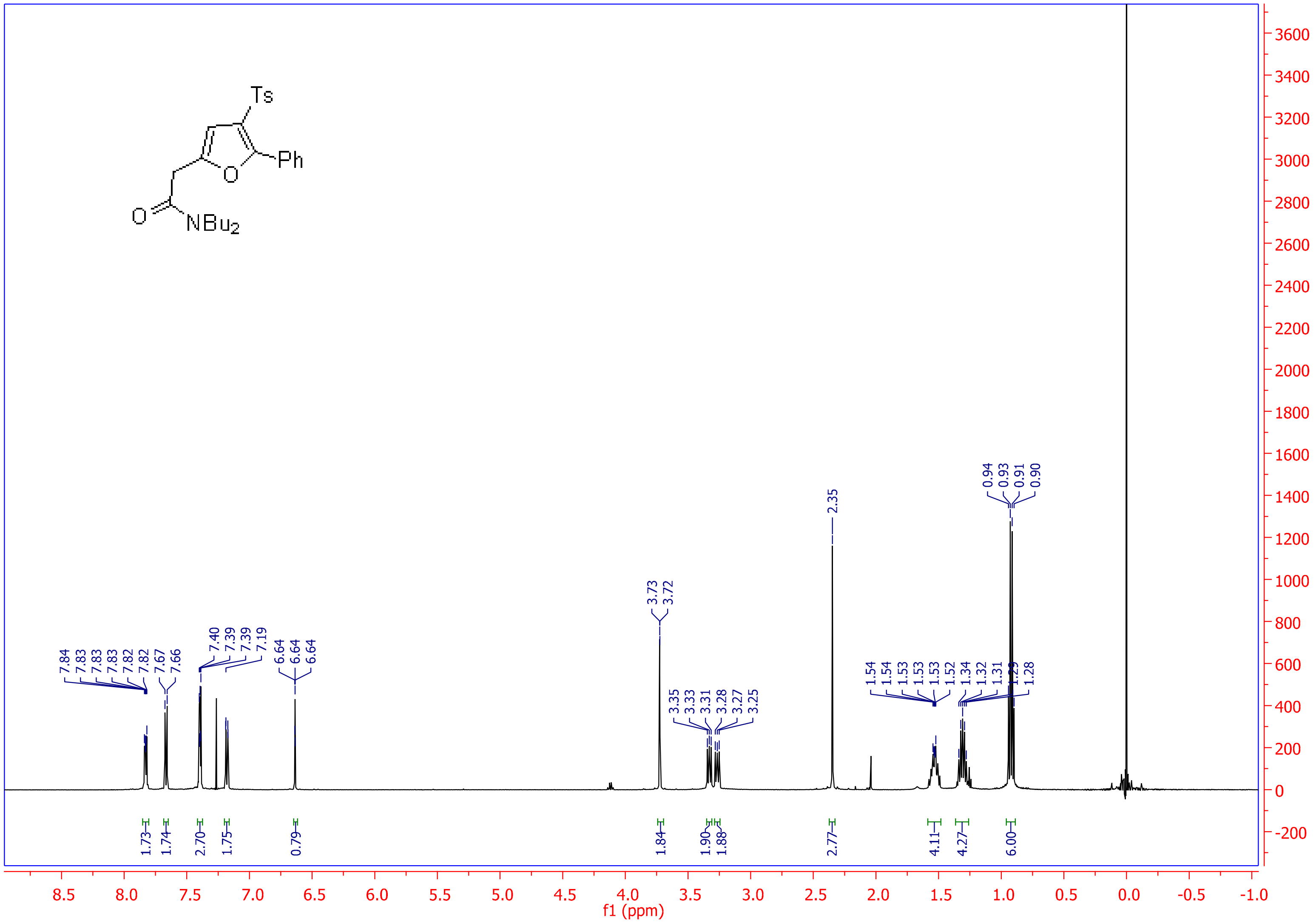

***N,N*-Dibutyl-2-(5-phenyl-4-tosylfuran-2-yl)acetamide (3nc)**

^{13}C NMR (125 MHz CDCl$_3$)

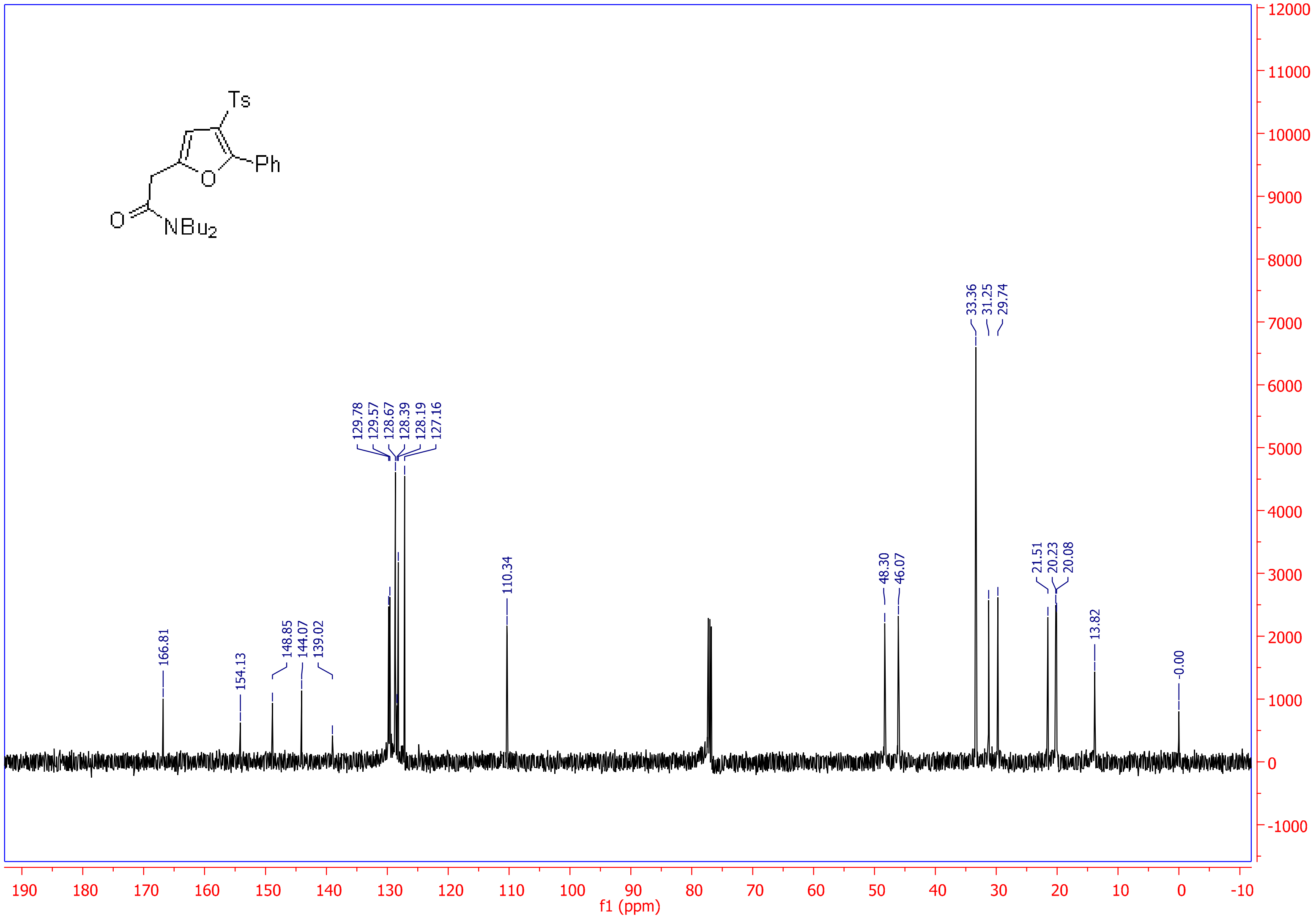

References

[33] Misztalewska, I.; Wilczewska, A. Z.; Wojtasik, O. K.; Markiewicz, H.; Kuchlewski, P.; Majcher, A. M. New Acetylacetone-Polymer Modified Nanoparticles as Magnetically Separable Complexing Agents. *RSC Adv.* **2015**, *5*, 100281-100289.

[34] Chang, M.-Y., Cheng, Y.-C.; Lu, W.-J. Bi(OTf)$_3$-Mediated Cycloisomerization of γ-Alkynyl Arylketones: Application to the Synthesis of Substituted Furans. *Org. Lett.* **2015**, *17*, 1264-1267.

[35] Schneider, L. M.; Schmiedel, V. M.; Pecchioli, T.; Lentz, D.; Merten, C.; Christmann, M. Asymmetric Synthesis of Carbocyclic Propellanes. *Org. Lett.* **2017**, *19*, 2310-2313.

[36] Gree, R.; Park, H.; Paquette, L. A. Regio- and Stereoselective 1,2 Wagner-Meerwein Shifts During Trifluoroacetic acid Catalyzed Isomerization of Unsymmetrically Substituted Tricyclo[3.2.0.02,4]heptanes. *J. Am. Chem. Soc*. **1980**, *102*, 4397-4403.

[37] Sanz, R.; Miguel, D.; A. Martínez; Álvarez-Gutiérrez, J. M., Rodriguez, F. Brønsted Acid Catalyzed Propargylation of 1,3-Dicarbonyl Derivatives. Synthesis of Tetrasubstituted Furans. *Org. Lett.* **2007**, *9*, 727-730.

[38] Ruengsangtongkul, S.; Chaisan, N.; Thongsornkleeb, C.; Tummatorn, J.; Ruchirawat, S. Rate Enhancement in CAN-Promoted Pd(PPh$_3$)$_2$Cl$_2$-Catalyzed Oxidative Cyclization: Synthesis of 2-Ketofuran-4-carboxylate Esters. *Org. Lett*. **2019**, *21*, 2514-2517.

[39] Li, W.; Wang, J.; Hu, X.; Shen, K.; Wang, W.; Chu, Y.; Lin, L.; Liu, X.; Feng, X. Catalytic Asymmetric Roskamp Reaction of α-Alkyl-α-diazoesters with Aromatic Aldehydes: Highly Enantioselective Synthesis of α-Alkyl-β-keto Esters. *J. Am. Chem. Soc.*, **2010**, 132, 8532-8533.

[40] Barabe, F.; Levesque, P.; Korobkov, I.; Barriault, L. Synthesis of Fused Carbocycles via a Selective 6-*Endo* Dig Gold(I)-Catalyzed Carbocyclization. *Org. Lett.* **2011**, *13*, 5580-5583.

[41] Katrun, P.; Songsichan, T.; Soorukram, D.; Pohmakotr, M.; Reutrakul, V.; Kuhakarn, C. *o*-Iodoxybenzoic Acid (IBX)–Iodine Mediated One-Pot Deacylative Sulfonylation of 1,3-Dicarbonyl Compounds: A Synthesis of β-Carbonyl Sulfones. *Synthesis* **2017**, *49*, 1109-1121.
